# Amodiaquine ameliorates stress-induced premature cellular senescence via promoting SIRT1-mediated HR repair

**DOI:** 10.1038/s41420-024-02201-1

**Published:** 2024-10-11

**Authors:** Jie Du, Fuqiang Chen, Chenghong Du, Wenna Zhao, Zihan Chen, Zhenhua Ding, Meijuan Zhou

**Affiliations:** 1 Jiangmen Central Hospital, Affiliated Jiangmen Hospital of Sun Yat-sen University, Jiangmen, Guangdong China; 2 Department of Radiation Medicine, Guangdong Provincial Key Laboratory of Tropical Disease Research, School of Public Health, Southern Medical University, Guangzhou, Guangdong China

**Keywords:** Senescence, High-throughput screening

## Abstract

DNA damage is considered to be a potentially unifying driver of ageing, and the stalling of DNA damage repair accelerates the cellular senescence. However, augmenting DNA repair has remained a great challenge due to the intricate repair mechanisms specific for multiple types of lesions. Herein, we miniaturized our modified detecting system for homologous recombination (HR) into a 96-well-based platform and performed a high-throughput chemical screen for FDA-approved drugs. We uncovered that amodiaquine could significantly augment HR repair at the noncytotoxic concentration. Further experiments demonstrated that amodiaquine remarkably suppressed stress-induced premature cellular senescence (SIPS), as evidenced by senescence-associated beta-galactosidase (SA-β-gal) staining or senescence‐related markers p21^WAF1^ and p16^ink4a^, and the expression of several cytokines. Mechanistic studies revealed that the stimulation of HR repair by amodiaquine might be mostly attributable to the promotion of SIRT1 at the transcriptional level. Additionally, SIRT1 depletion abolished the amodiaquine‐mediated effects on DNA repair and cellular senescence, indicating that amodiaquine delayed the onset of SIPS via a SIRT1-dependent pathway. Taken together, this experimental approach paved the way for the identification of compounds that augment HR activity, which could help to underscore the therapeutic potential of targeting DNA repair for treating aging-related diseases.

## Introduction

Cellular senescence occurs in response to endogenous and exogenous stressful stimuli by terminating the cell cycle circulation. In addition to replicative senescence as a result of telomere shortening [1], another type of senescence named stress-induced premature senescence (SIPS) has been shown to be involved in various pathological conditions such as cancer and fibrosis [2, 3]. Importantly, SIPS could be induced by multiple triggers, including oncogene mutation or activation, mitochondrial dysfunction, viral infection, and different agents leading to the accumulation of DNA damage such as genotoxic drugs or ionizing radiation [4–6].

Among the multifaceted causes associated with aging, DNA damage impinges most, if not all, aspects of aging phenotype [7]. DNA double-strand breaks (DSBs) are the most harmful type of DNA lesions, which could lead to chromosome instability and senescence if not adequately repaired [8]. Eukaryotes have evolved two main mechanisms responsible for DSB repair: non-homologous end joining (NHEJ) and homologous recombination (HR) [9]. NHEJ directly processes and ligates the broken DNA ends together in an imprecise manner and requires several factors such as Ku70/80, DNA-PK, and Ligase IV. On the other hand, HR serves as an accurate repair pathway dependent upon the guidance of the intact sequence information from the sister chromatid [10]. For HR repair, it commences with the end resection regulated by MRE11, NBS1, and RAD50 complex. Once the ends are resected, Rad51 forms nucleoprotein filaments and facilitates strand invasion into the sister chromatid, a process restricted to the S and G2 phases of the cell cycle [10, 11].

The declined DSB repair by both pathways leads to cascading accumulation of DNA damage and mutations, which eventually exacerbate the age-related dysfunction [12]. Actually, most progeroid (premature ageing-like) syndromes are caused by mutations of genes involved in the maintenance of genome stability. The deficiency of ATM or NBS, both of which serve as central roles in DSB repair progress, result in the progeroid conditions ataxia-telangiectasia syndrome and Nijimegen breakage syndrome, respectively [13–16]. Currently, it was proposed that specifically reducing Rad51-mediated HR could lead to premature aging and decreased lifespan in vivo [17]. Thus, it might be a potential strategy to delay the onset of SIPS by augmenting DSB repair. High-throughput screening has been executed to identify a variety of small molecule compounds involved in the regulation of DSB repair. For instance, RS-1 emerges as a potent enhancer of CRISPR-based genome editing by promoting HR, while farrerol improves HR repair by promoting Rad51 recruitment [18, 19]. However, the suppressive effects of these compounds on senescence via augmenting DSB repair are still elusive.

With this idea in mind, we developed a cell-based screening for an FDA-approved drug library and identified three hit compounds exhibiting remarkable augmentation of HR activity in vitro. Then we focused on amodiaquine (AQ) and found that in addition to promotion of HR repair, it delayed the onset of SIPS and reduced the expression of the senescence-associated secretory phenotype (SASP) factors. These effects seemed to be attributed to the stimulation of SIRT1 transcription. Interestingly, SIRT1 depletion abolished AQ‐mediated effects on DNA repair and cellular senescence. Further mechanistic studies indicated that AQ stimulated the recruitment of Rad51 and accelerated the efficiency of DNA repair without changing the expression levels of NHEJ- and HR-related factors. Our work might provide a new strategy for drug repurposing, which could help develop potential strategies to ameliorate stress-induced premature cellular senescence.

## Results

### Identification of small molecules that alter HR repair

We previously established a novel method for quantitative assessment of HR and NHEJ activities via CRISPR/Cas9-induced oligodeoxynucleotide-mediated DSB repair [20]. And omipalisib was identified as a potent NHEJ inhibitor for improving the therapeutic efficiency of radio- or chemotherapy [21]. Unlike NHEJ, HR activity is more difficult to estimate since its function is believed to restrict only during the S and G2 phases of the cell cycle when the homologous template is available. Recent studies revealed that Fanconi anemia (FA) pathway plays a key role in Cas9-induced single-strand template repair (SSTR), and knockdown of Rad51 abolished dsDNA donor HDR but had no effect on SSTR [22]. Therefore, we chose dsDNA donor as the template for detecting HR activity in the present study. Moreover, we optimized the screening protocol using the sgRNA targeting the gene loci of Adeno-Associated Virus Integration Site 1 (*AAVS1*) instead of hypoxanthine-guanine phosphoribosyltransferase (*HPRT*), by which the higher basal HR activity could be achieved (Fig. S1A, B).

To confirm the feasibility of the screening workflow, we first carried out this assay to examine the suppressive effects of Rad51 inhibitor RI-1 on HR pathway [23]. As shown in Fig. S1C, the dose-dependent inhibitory effects on HR activities were unequivocally demonstrated. NU7441 is a selective inhibitor of the NHEJ pathway protein DNA-dependent protein kinase that has been shown to increase the rate of HR following Cas9-mediated DNA cleavage [24]. Since NHEJ inhibition could shift the balance of DSB repair toward HR, NU7441 significantly improved the efficiency of Cas9-mediated HR in a concentration-dependent manner (Fig. S1D).

**Fig. 1:**
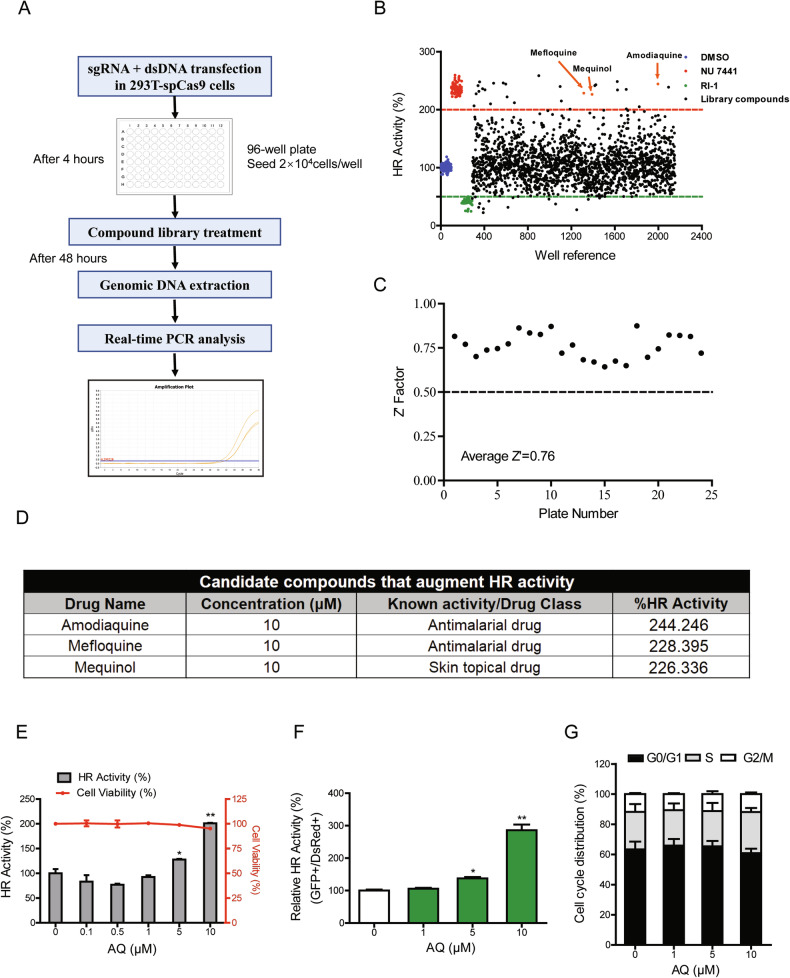
High-throughput screening of compounds that modulate HR activity. **A** Schematic depicting the screening procedures. **B** Scatter plots of the screening results of 1865 compounds. **C** Z′ factors were calculated from the mean and standard deviations of positive NU7441 control and negative vehicle control samples for each screening plate. **D** The detailed information of three candidates was shown. **E** The cytotoxic and HR-promoting effects of AQ were displayed in a dose-dependent manner. **F** Measurement of HR efficiency by using the DR-GFP reporter assay. HR efficiency was calculated as the ratio of GFP^+^/DsRed^+^ cells by flow cytometry. **G** Flow cytometry assay with PI staining showed the cell cycle distribution of HFF1 cells after 48 h of AQ exposure. Significance markers: *p < 0.05; **p < 0.01 compared to control; *n* = 3.

Next, we performed a high-throughput screen of an FDA-approved drug library, which includes 1865 approved drugs (Fig. 1A). The pilot screening results were depicted as scatter plots in Fig. 1B, and all the data were listed in supplementary Table S1. Each plate contained the negative control consisted of cells treated with 2% DMSO, as well as the positive control compounds that are known to augment or decrease HR activity, Nu7441 and RI-1, respectively. The threshold of potential hits was chosen as 4SD for the selection of top hits in the primary screening. Additionally, the Z′-factor for individual plate were measured to monitor the screening quality, which yielded an average value of 0.76 (95% confidence interval, 0.71–0.82), suggesting excellent screening quality (Fig. 1C). Following primary screening, three compounds were identified that increased HR repair, including AQ, mefloquine, and mequinol (Fig. 1D).

### Validation demonstrating that amodiaquine augments HR repair

To affirm the screening results, we investigated the dose-dependent effects of positive hits on HR repair. Meanwhile, the cytotoxic effects of the three compounds were evaluated by CCK-8 cell viability assay (Figs. 1E and S1E). Among these, AQ exhibited the most robust facilitation effect on HR activity at a noncytotoxic concentration, while having no impact on NHEJ (Figs. 1E and S2A). This investigation was further corroborated by using the HR and NHEJ reporter assay, in which successful HR/NHEJ repair of I-SceI nuclease-induced DSBs would restore the expression of the intact GFP gene (Figs. 1F and S2A). Propidium iodide (PI) staining demonstrated that AQ did not impact cell cycle distribution (Fig. 1G), indicating the augmentation of HR repair mediated by AQ was not due to cell cycle arrest. Moreover, there were no significant changes in apoptosis rates, underscoring the low toxicity of AQ (Fig. S2B).

**Fig. 2:**
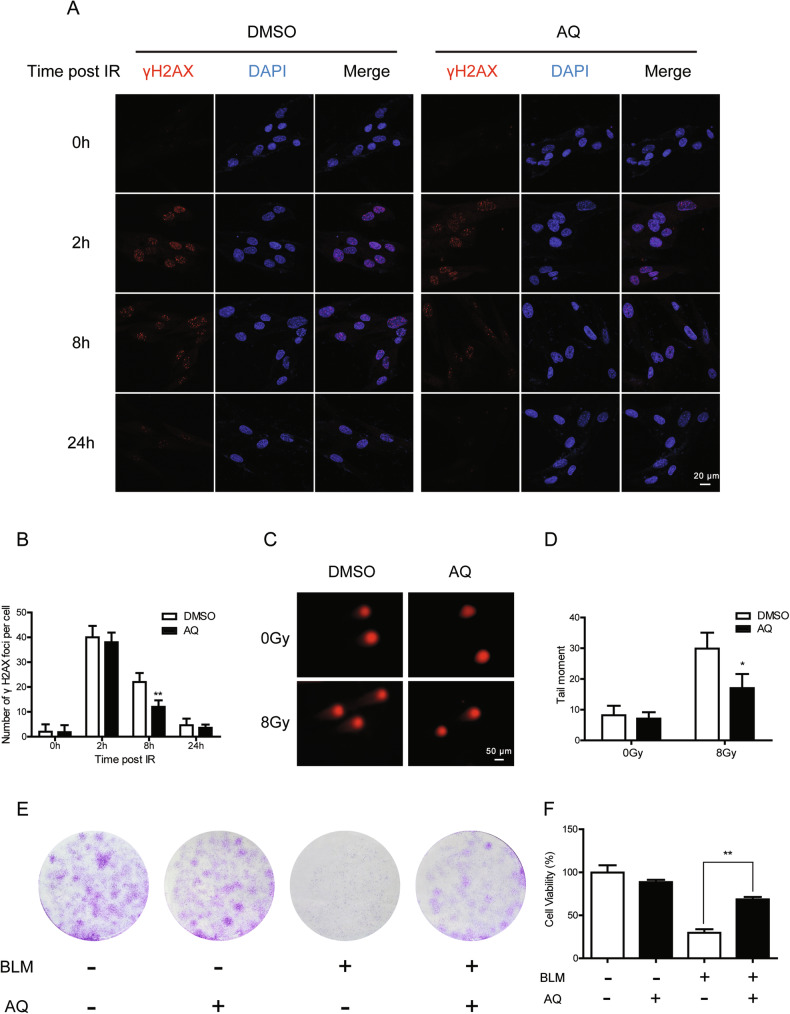
The effects of amodiaquine on DSB repair and cell survival. **A** HFF1 cells were irradiated with 4 Gy of X-ray after a 24-h period of AQ treatment. Cells were immunostained for gH2AX foci (red) at indicated time points. **B** The number of γH2AX foci per cell. **C**, **D** HFF1 cells were incubated with 10 μM AQ for 24 h, followed by exposure to 8 Gy of X-rays. Neutral comet assay was performed 8 h post irradiation. The representative images (**C**) and quantification of the tail moment (**D**) were shown. **E** The clonogenic survival assay of HFF1 cells treated with AQ followed by bleomycin (10 μM) treatment. **F** Cell viability of Beas-2B cells treated with AQ followed by bleomycin treatment. Significance markers: *p < 0.05; **p < 0.01 compared to control; *n* = 3.

Next, we sought to determine whether AQ could promote the DNA repair of the IR-induced DSBs. Human foreskin fibroblast cells (HFF1) were pretreated with 10 μM AQ 24 h prior to X-ray exposure and further incubated for 24 h. Cells were fixed at different time points post-irradiation and γ-H2AX foci was immunostained and quantified. As shown in Fig. 2A, B, an approximately twofold reduction in the number of γH2AX foci was observed in AQ-treated cells 8 h post IR, indicating that AQ accelerated the repair of X‐ray‐induced DSBs. Similarly, neutral comet assays revealed that AQ promoted the repair of damaged DNA, as evidenced by the decreased DNA content in the comet tails (Fig. 2C, D). As a genotoxic agent, bleomycin exerts its cytotoxicity via generating DSB lesions [25]. Compared with that of bleomycin treatment alone, the combination of AQ and bleomycin led to a remarkable increase in cell colony formation of HFF1 cells (Fig. 2E). Additionally, in the presence of AQ, the cell viability of Beas-2B cells was notably enhanced (Fig. 2F). These results confirmed that the cytotoxic activity of bleomycin could be alleviated by AQ through augmenting DSB repair. While testing toxicity in cancer cells, AQ exhibited robust inhibitory effect on A549 and HCT116 cells in a dose-dependent manner (Fig. S2C), which might hold potential application of AQ in radioprotection or cancer therapy.

### Amodiaquine suppresses stress-induced cellular senescence

**Fig. 3:**
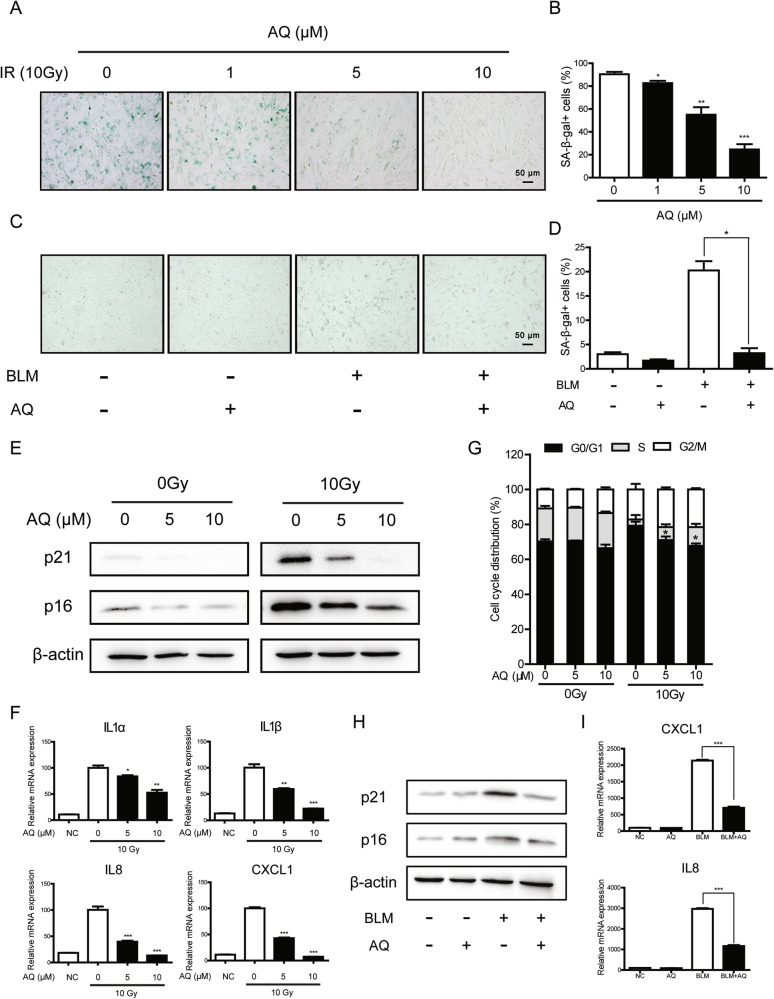
Amodiaquine suppresses stress-induced cellular senescence. **A**–**D** HFF1 cells were pretreated with the indicated doses of AQ for 24 h prior to X-ray exposure and further incubated for 7 days (**A**, **B**); Beas-2B cells were pretreated with AQ for 24 h prior to 10 μM bleomycin exposure and further incubated for 3 days (**C**, **D**). The representative images (**A**, **C**) and quantitative analyses (**B**, **D**) of SA-β-Gal staining were shown. **E**, **F**, **H**, **I** HFF1 cells were irradiated with 10 Gy of X-ray after a 24-h period of AQ treatment and further incubated for 5 days (**E**, **F**); Beas-2B cells were exposed to 10 μM bleomycin after a 24-h period of AQ treatment and further incubated for 3 days (**H**, **I**). The protein expression of p16 and p21 was analysed by Western blotting (**E**, **H**) and the relative mRNA expression of several SASP factors (IL1α, IL1β, IL8, and CXCL1) was analysed by qRT‐PCR (**F**, **I**). **G** HFF1 cells were pretreated with the indicated doses of AQ for 24 h prior to X-ray exposure and further incubated for 5 days. Cell cycle distributions were examined by flow cytometry. Significance markers: *p < 0.05; **p < 0.01; ***p < 0.001 compared to control; *n* = 3.

Recent evidence has highlighted that promoting DNA repair could delay ageing and age-associated pathologies [26, 27]. Therefore, we hypothesized that AQ may mitigate stress-induced premature cellular senescence induced by radiation or genotoxic drugs. HFF1 cells were pretreated with 10 μM AQ 24 h prior to X-ray exposure and further incubated for 7 days. As shown in Fig. S2F, the intensity of positive SA‐β‐Gal staining increased following exposure to radiation in the DMSO-treated samples. However, we observed remarkable reduction in β-gal-positive HFF1 cells after treatment with AQ in a dose-dependent manner (Figs. 3A, B and S2F). And it seemed to exhibit no significant effect on the proliferation and apoptosis of HFF1 cells (Fig. S2D, E). Similarly, bleomycin-induced senescence of Beas-2B cells was attenuated in the presence of AQ (Fig. 3C, D). Consistent with these results, AQ-treated cells showed lower expression levels of senescence‐related markers p21^CIP1/WAF1^ and p16^INK4A^ after 10 Gy X-ray or bleomycin administration (Fig. 3E, H). Furthermore, PI staining showed that HFF1 cells treated with AQ displayed a lower level of G1 phase arrest 5 days after 10 Gy X-ray administration (Fig. 3G).

SASP is one of the hallmarks of cellular senescence, which is composed of various cytokines, chemokines, growth factors, and proteases. Additionally, several cytokines, including IL1α, IL1β, IL8, and CXCL1, were assessed to evaluate the SASP upon DNA damage. Quantitative real-time PCR indicated that AQ reduced the expression of SASP genes in senescent cells (Fig. 3F, I). Taken together, these results provided additional support that the identified candidate AQ could delay the onset of SIPS and alleviate the SASP.

### Amodiaquine affects HR repair by upregulating SIRT1 expression at the transcriptional level

To further elucidate the regulatory mechanisms of AQ on DSB repair, we first examined the expression levels of critical proteins involved in NHEJ and HR repair. We did not observe any significant alternations in the expression of NHEJ factors (DNA-PKcs, Ligase IV, Ku80, and Ku70) or HR factors (BRCA2, BRCA1, CtIP, Mre11, RAD50, NBS1, Rad54, Rad51, and RPA2) upon AQ treatment (Fig. S3A, B). Then we hypothesized that AQ might activate upstream factors participating in DSB repair.

**Fig. 4:**
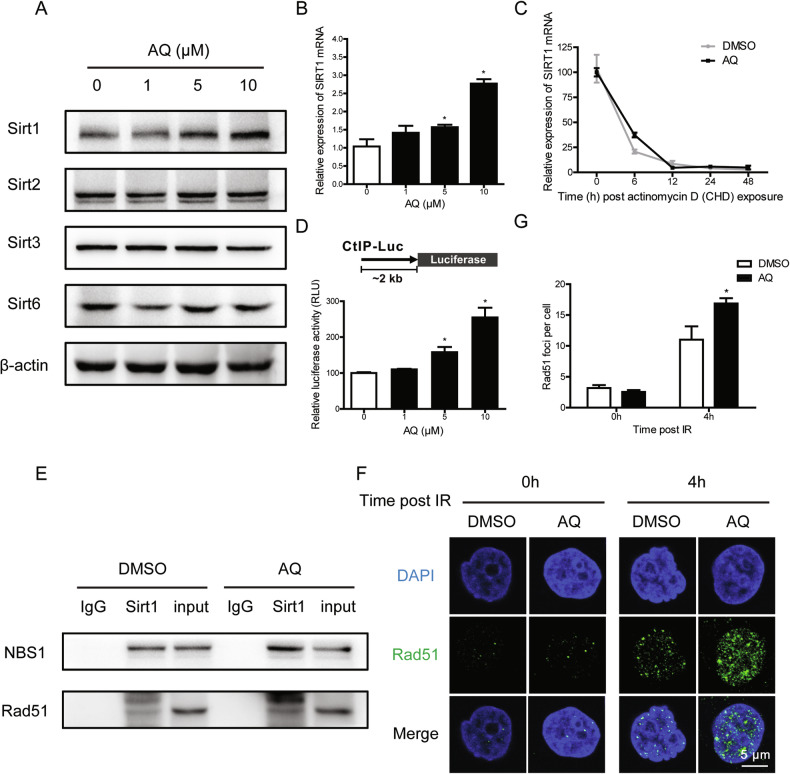
Amodiaquine enhances SIRT1 expression at the transcriptional level. **A**, **B** HFF1 cells were treated with 0, 1, 5, and 10 μM AQ for 48 h. The expression levels of SIRT1, SIRT2, SIRT3, and SIRT6 were determined by Western blotting (**A**); and SIRT1 mRNA were measured by qRT-PCR (**B**). **C** HFF1 cells were treated with 5 μg/ml CHD alone or in combination with AQ for 0, 6, 12, 24 and 48 h. Subsequently, the relative mRNA of SIRT1 was evaluated by qRT-PCR. **D** HFF1 cells were pretreated with AQ 24 h prior to transfection with the luciferase reporter plasmids. The relative luciferase activity normalized to Renilla luciferase activity is presented. **E** Cell lysates were immunoprecipitated with anti-SIRT1 antibody after treatment with AQ for 48 h, followed by immunoblotting with anti-NBS1 and anti-Rad51 antibodies. **F** HFF1 cells were irradiated with 4 Gy of X-ray after a 24-h period of AQ treatment. Cells were immunostained for Rad51 foci (green) at 4 h post irradiation. **G** The number of Rad51 foci per cell. Significance markers: *p < 0.05 compared to control; *n* = 3.

Considering that SIRTUIN family has been deemed to be involved in the regulation of both cellular senescence and DNA damage response through various mechanisms [28–30], we proceeded to investigate the potential effects of AQ on the expression of SIRT1, SIRT2, SIRT3, and SIRT6. Surprisingly, AQ treatment noteworthily increased SIRT1 expression in a dose-dependent manner (Fig. 4A), and we did not observe any significant alterations in the expression of other HDAC classes upon AQ exposure (Fig. S3C). Quantitative PCR analysis revealed that the upregulation of SIRT1 protein levels induced by AQ occurred at the transcriptional level (Fig. 4B), and it seemed irrelevant to the degradation rates of SIRT1 mRNA (Fig. 4C). Furthermore, luciferase assays demonstrated that AQ enhanced SIRT1 promoter activity, subsequently leading to an elevation in SIRT1 mRNA levels (Fig. 4D).

Considering that SIRT1 has been proven to support HR repair through regulating HR repair machinery proteins, including NBS1 and Rad51 [31], we further measured this association after treatment of AQ. Expectedly, immunoprecipitation showed that more SIRT1-NBS1-Rad51 complex was formed due to elevated expression levels of SIRT1 in the AQ-treated group (Fig. 4E). Interestingly, AQ treatment promoted the recruitment of Rad51 upon DNA damage (Fig. 4F, G), which could be partially counteracted by SIRT1 knockdown (Fig. S3D, E). The detailed molecular mechanisms of AQ-mediated HR promotion via SIRT1 and Rad51 need to be further studied.

### SIRT1 depletion abolishes the Amodiaquine‐mediated effects on DNA repair and cellular senescence

**Fig. 5:**
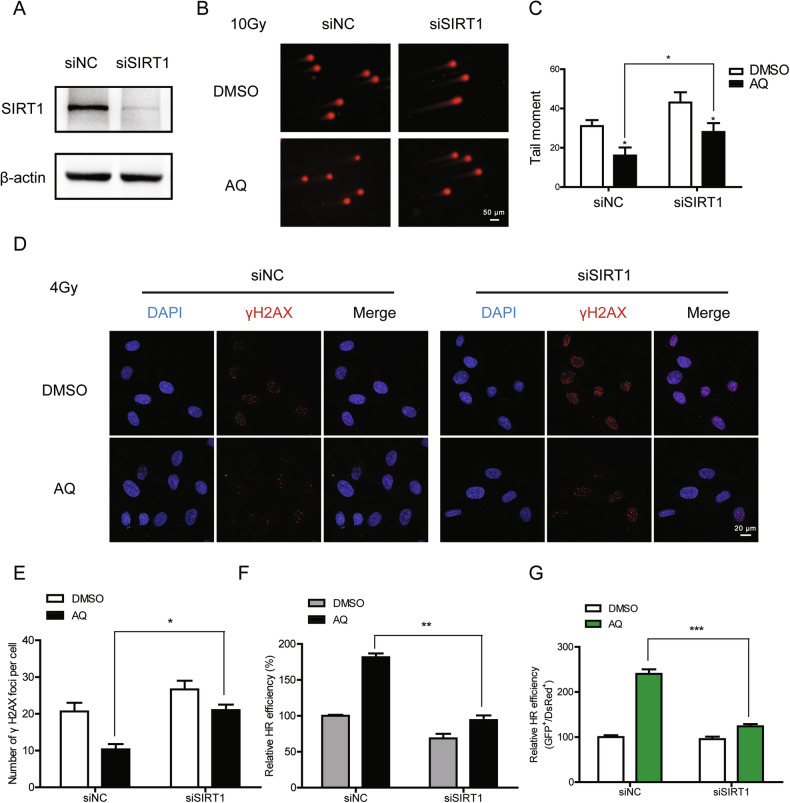
SIRT1 depletion abolishes the amodiaquine‐mediated effects on DNA repair. **A** Western blotting of the SIRT1 expression in HFF1 cells transfected with control or Rad51 siRNA. **B**, **C** The SIRT1-depleted cells were pretreated with AQ for 24 h prior to 8 Gy of X-ray and further incubated for 8 h, then the cells were collected for neutral comet assay. The comet assay images (**B**) and quantification of tail moment (**C**) were shown. **D**, **E** The SIRT1-depleted cells were pretreated with AQ for 24 h prior to 4 Gy of X-ray. Immunofluorescent staining for γH2AX foci (red) were performed 8 h post irradiation. The representative images (**D**) and the number of γH2AX foci per cell (**E**) were presented. The SIRT1-depleted cells were treated with indicated conditions and subjected to HR efficiency evaluation by using our detecting system (**F**) and DR-GFP reporter assay (**G**). Significance markers: *p < 0.05; **p < 0.01; ***p < 0.001 compared to control; *n* = 3.

Since SIRT1 showed robust enhancement following AQ treatment, we hypothesized that depletion of SIRT1 may nullify the promotion of DSB repair by AQ. As depicted in Fig. 5A-C, AQ-mediated reduction in the tail moment was abolished after silencing of SIRT1. Consistently, SIRT1 depletion impeded the resolving of γ-H2AX foci of AQ-treated cells (Fig. 5D, E), indicating the pivotal role of SIRT1 in the AQ-mediated promotion of DSB repair. Basing on our HR detecting system and DR-GFP reporter assay, we also found that depleting SIRT1 completely diminished AQ-mediated stimulation of HR repair (Fig. 5F, G). These results were further supported by the treatment of EX527, which serves as a selective SIRT1 inhibitor (Fig. S4A-D).

**Fig. 6:**
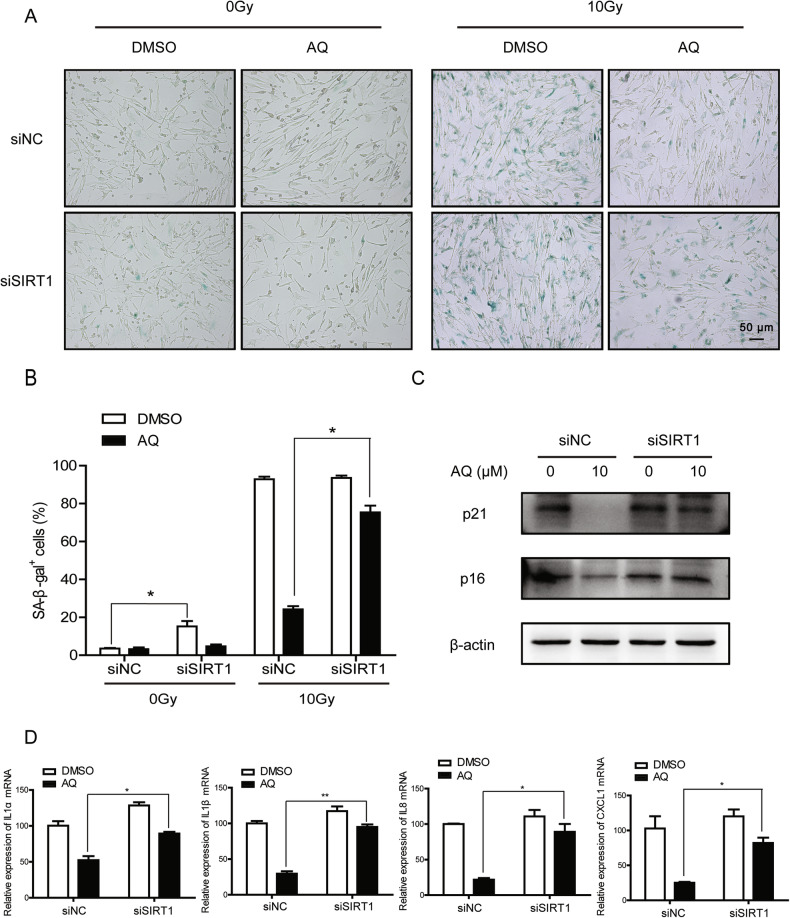
SIRT1 depletion abolishes the amodiaquine‐mediated suppression on SIPS. **A**, **B** The SIRT1-depleted cells were pretreated with AQ for 24 h prior to 0 Gy or 10 Gy of X-ray exposure and further incubated for 7 days. The representative images (**A**) and quantitative analyses (**B**) of SA-β-Gal staining were shown. **C**, **D** The SIRT1-depleted cells were pretreated with AQ for 24 h prior to X-ray exposure and further incubated for 5 days. The protein expression of p16 and p21 was analysed by Western blotting (**C**), and the relative mRNA expression of SASP factors (IL1α, IL1β, IL8, and CXCL1) was analysed by qRT‐PCR (**D**). Significance markers: *p < 0.05; **p < 0.01 compared to control; *n* = 3.

For decades, accumulating evidence has disclosed that SIRT1 could regulate aging and aging-related diseases through multiple signaling pathways including oxidative stress response, DNA damage repair, autophagy, and metabolic dysfunction [32]. To ensure that AQ activates SIRT1 to attenuate premature cellular senescence in HFF1 cells, we used siRNA to silence SIRT1 to investigate the suppressive effect of AQ on SIPS and SASP. The intensity of positive SA‐β‐Gal staining increased mildly after SIRT1 knockdown in HFF1 cells, and further rescue experiments demonstrated that both SIRT1 depletion and enzyme activity blocking could reverse AQ-mediated inhibition of SIPS, as illustrated by SA-β-Gal staining (Figs. 6A, B and S4E, F). And the expression levels of cellular senescence biomarkers p16^INK4A^ and p21^CIP1/WAF1^ in SIRT1-depleted cells were not ameliorated despite AQ treatment during irradiation (Fig. 6C). Consistently, AQ-induced reduction of SASP factor expression was partially rescued by SIRT1 knockdown (Fig. 6D), indicating that AQ inhibits IR-induced cellular senescence through a SIRT1-dependent pathway. Similar results were observed in cells treated with EX527 (Fig. S4G).

Taken together, our findings show that AQ augments HR-mediated DNA repair by promoting SIRT1 transcription, thereby suppressing the onset of SIPS and the expression of SASP factors.

## Discussion

Over the past decades, great attention has been paid to age-related disease drug discovery, since interventions targeting senescent cells could ameliorate a variety of human conditions [33]. To name a few, natural compounds such as quercetin, resveratrol, and spermidine have been reported to prolong the lifespan of models such as yeasts, flies, and mice [34]. Some other agents such as senolytics, metformin, and NAD^+^ supplements also demonstrated their effectiveness in delaying, preventing, or alleviating chronic diseases and disabilities of aging in preclinical models [26, 35, 36]. Importantly, accumulating evidence suggested that genomic stability could be improved during the implementation of the above interventions, which indicated the central role of DNA damage in the aging process.

By means of various analysis approaches, several studies suggested that the efficiency of DNA damage repair declined with age in various organs and tissues of mice and humans [37–39]. Reduction of DNA repair efficiency and fidelity leads to more mutations, and further exacerbates the age-related functional decline, which contribute to the vicious cycle of aging and genomic instability [40, 41]. In the case of that, activated DNA repair has great potential to alleviate ageing and age-associated pathologies. In this study, we optimized our previously established detecting platform and performed a chemical screen to identify compounds that alter HR. As a result of this screening, AQ, an FDA-approved antimalarial drug, showed the most potent stimulative effects on DSB repair. In addition to its known actions, AQ was found to augment HR activity at a noncytotoxic concentration but have no significant effect on NHEJ. As expected, AQ suppressed radiation-induced senescence phenotypes such as increased SA β-gal staining and SASP in HFF1 fibroblast. Our latest study revealed that the decrease of Rad51 plays a pivotal role in bleomycin-induced AECs senescence and lung injury [42]. Interestingly, AQ notably suppressed bleomycin-induced premature cellular senescence in Beas-2B cells, which could further extend its potential application in bleomycin-induced pulmonary fibrosis in vivo experiment.

Recently, great attention has been paid to the repurposing of FDA-approved compounds to identify new potential drugs [43, 44]. Although AQ are well-established members of the 4-aminoquinolines family of antimalarial drugs, multiple mechanisms of antitumor function of AQ have been reported, including autophagy inhibition, induction of autophagic-lysosomal blockade, and suppression of ribosome biogenesis [44, 45]. Notably, AQ has been reported to stimulate the transcriptional function of nuclear receptor-related 1 protein (Nurr1) through physical interaction with its ligand binding domain, which could further improve behavioral deficits and cognitive function of Parkinson’s disease and Alzheimer’s disease in the animal model, both of which are associated with aging [46, 47]. As a result of our screening for compounds that alter HR repair, we initially explored its potential regulatory action on DSB repair pathways. Mechanistic studies indicated that AQ treatment did not display an obvious effect on the expression of HR and NHEJ pathway-associated factors, and the promotion of HR by AQ was not attributed to arrest cells in the HR-dominant S/G2 phase. Mounting evidence indicated that SIRT1 could promote HR activity through interacting or deacetylating HR repair machinery proteins, including BRG1, NBS1, and Rad51 [28, 31]. Significantly, we observed a remarkable elevation in SIRT1 expression following exposure to AQ, accompanied by increased formation of SIRT1-NBS1-Rad51 complex and stimulated recruitment of Rad51, which may contribute to the promotion of HR by AQ. Nonetheless, the detailed mechanisms of physical interaction between AQ and SIRT1 need to be further confirmed.

Sirtuin, also referred to as class III HDAC (histone deacetylases), has been deemed to be involved in the regulation of cellular senescence and organismal lifespan [30, 48]. Despite not being a typical enzyme involved in DNA repair, it serves as an essential factor in the maintenance of genome stability and DNA damage repair [49, 50]. SIRT6 loss leads to genomic instability and aging-like phenotype, while impaired DNA damage response and tumorigenesis could be observed in SIRT1 mutant mice [51, 52]. Our results demonstrated that SIRT1 depletion abolished the AQ‐mediated effects on DNA Repair and cellular senescence, indicating that AQ delayed the onset of SIPS via a SIRT1-dependent pathway. Although numerous studies have pointed out age-related reduction in the expression of DNA repair factors or their activities, directly supplementing downstream DNA repair enzymes is controversial, on account of toxicity and unavailability [40, 53]. Interventions in upstream regulators such as sirtuins might be a more suitable approach to ameliorate premature ageing phenotypes associated with stalled DNA repair. For example, overexpression of SIRT6 instead of Rad51 or NBS1 rescues HR repair in ageing cells [40], and restoration of the NAD^+^/SIRT1 pathway improves healthspan in Ataxia-telangiectasia models via mitophagy and DNA repair [26]. Lycorine, a natural compound of Amaryllidaceae family, augments the DSB repair via promoting the transcription of SIRT1 and SIRT6, thereby restraining stress-induced senescence [27]. Taken together, augmenting DSB repair through reinforcing sirtuins might be a promising approach to alleviate cellular senescence.

In conclusion, we established a cell-based screening platform for small molecules that altered HR repair and identified three candidates through screening an FDA-approved drug library. Then we focused on the effects of AQ on cellular senescence and revealed that AQ promoted HR repair via enhancing SIRT1 expression at the transcriptional level, resulting in the suppression of SIPS and associated SASP. This proof-of-concept study contributes to the identification of novel molecules that augment HR repair, which may help to develop potential strategies to alleviate ageing and aging-related diseases.

## Materials and methods

### Cell culture and reagents

Human fibroblast HFF1 cells, human lung epithelial cell line Beas-2B, HEK293T cells, human non-small-cell lung cancer cell line A549, and colorectal carcinoma cell line HCT116 were obtained from the Cell Bank of the Chinese Academy of Sciences. HEK293T-spCas9 cells were purchased from OBiO Technology (Shanghai, China). Cells were cultured in Dulbecco’s modified Eagle’s medium supplemented with 15% fetal bovine serum (HFF1) or 10% fetal bovine serum (HEK293T and HEK293T-spCas9), 100 mg/ml streptomycin and 100 U/ml penicillin at 37 °C in a humidified 5% CO_2_ incubator.

The siRNA duplexes against SIRT1 (5′-GCCTGATGTTCCAGAGAGA-3′) were synthesized by RiboBio (Guangzhou, China). AQ, NU7441, RI-1, bleomycin, EX527, actinomycin D (CHD), and an FDA-approved drug library containing 1865 compounds were purchased from Selleck Chemicals (Houston, TX, USA). All compounds were dissolved in dimethyl sulfoxide (Sigma, St. Louis, MO, USA) to obtain a stock solution, stored at −20 °C, and diluted to the desired concentration in fresh medium immediately before use.

### T7 endonuclease 1 (T7E1) assay

Three different sgRNA (sgRNA1: GTCCCCTCCACCCCACAGT, sgRNA2: GGGCCACTAGGGACAGGAT, and sgRNA3: GGGGCCACTAGGGACAGGAT) targeting the AAVS1 locus were synthesized by GENEWIZ company (Suzhou, China). T7 Endonuclease 1 (T7E1) assay was performed to assess the efficiency of CRISPR/Cas9 mutagenesis. Briefly, three distinct sgRNA was transfected into HEK293T-spCas9 cells respectively. The genomic DNA were collected 48 h after transfection and CRISPR/Cas9-targeted site was amplified by PCR. Next, the PCR amplicons were digested by 5 units of T7E1 according to the manufacturer’s protocol (ViewSolid Biotech) and immediately subjected to 2% agarose gel electrophoresis.

Compared with the control group, sgRNA1 exhibited efficient cleavage activities as determined by the T7E1 assay. And sgRNA1 was used in the following experiments.

### Quantitative measurement of HR and NHEJ activity

The schematic diagram of the quantitative method for measuring HR and NHEJ activities has been previously described [20]. In the present study, HEK293T-spCas9 cells were transfected with sgRNA targeting *AAVS1* and dsDNA (HR) or dsODN (NHEJ) by Lipofectamine 2000 Reagent (Invitrogen, Carlsbad, CA, USA). Following the induction of site-specific DSBs, exogenously introduced dsDNA or dsODN harboring a unique marker sequence is embedded in the DSB sites via HR or NHEJ pathway. Next, by means of PCR analysis using a specific primer for the marker sequence (M: GAGTTGTCATATGTTAATAACGGTAT) and the primers that flank the DSB sites (F: ACCTTATATTCCCAGGGCCG, R: ATGGGGGTGTGTCACCAGAT), the activities of HR/NHEJ repair could be quantitatively measured by detecting the relative amount of integrated marker sequence in the genomic DNA.

### High-throughput screening process

Four hours after transfection, HEK293T-spCas9 cells were seeded into 96-well plates and treated with the drug library in a final concentration of 10 μM for 48 h. Genomic DNA extraction was performed by using Chelex-100 ion exchange resin (Sigma), then further delivered to quantitative PCR analysis as previously described. The screening quality was evaluated by Z′-factor, defined as 1 – [3(*σ*_c+_ + *σ*_c−_)/|*μ*_c+_ − μ_c−_|], where σ_c+_ and σ_c−_ are the standard deviations of the values of the NU7441 and DMSO-treated samples, and μ_c+_ and μ_c-_ are the means of the values of the NU7441 and DMSO-treated samples.

### Cell viability and colony formation assay

After treated with indicated conditions, cells were incubated with 10 μL CCK-8 (Dojindo, Japan) solution at 37 °C for 2 h. The absorbance was measured at 450 nm using a spectrophotometer.

HFF1 cells were counted and plated into 6-well plate in triplicate. The next day, cells were incubated with AQ for 24 h, then bleomycin administration was performed. Twenty-four hours later, the cells were replaced with fresh medium and further cultured for 7–14 days and stained with 1% crystal violet. Colonies consisting of more than 50 cells were regarded as a single colony.

### HR and NHEJ reporter plasmid assay

The HR and NHEJ reporter plasmid assays were conducted as previously described [54]. In brief, plasmids linearized with I-SceI were co-transfected alongside pCMV-DsRed plasmids into HEK293T cells using Lipofectamine 2000 Reagent (Invitrogen) for 72 h. Fluorescently labeled cells were monitored by the BD LSRFortessa X-20 Flow Cytometer (BD Biosciences, San Jose, CA, USA). The activities of HR and NHEJ were quantified by calculating the ratio of GFP-positive cells relative to DsRed-positive cells.

### Flow cytometry analysis

For cell cycle analysis, HFF1 cells were collected and fixed in 70% ethanol overnight at −20 °C. After fixation, cells were stained with PI/ribonuclease staining buffer (BD Biosciences) for 20 min at room temperature in the absence of light. For apoptosis analysis, cells were harvested and stained with Annexin V-FITC apoptosis detection kit (BD Biosciences). All samples were analysed using the BD LSRFortessa X-20 flow cytometer (BD Biosciences).

### Immunofluorescence

HFF1 cells were fixed with 4% paraformaldehyde in the presence of 0.2% Triton X-100 for 20 min at different time points. Primary antibodies against phosphorylated histone H2A variant (γH2AX, #ab22551), or Rad51 (#ab133534, Abcam, Cambridge, UK), was diluted 1:500 to immunostain the cells at 4 °C overnight. After washing, the cells were further incubated with Alexa Fluor 555-conjugated goat anti-mouse IgG or Alexa Fluor 488-conjugated goat anti-rabbit IgG (Invitrogen). Finally, the foci were counted in 100 randomly selected cells for each group and images were obtained using an FV3000 confocal microscope (Olympus, Tokyo, Japan).

### Neutral comet assay

The Comet Assay Kit (Trevigen, Gaithersburg, MD, USA) was utilized to perform neutral comet assays. HFF1 cells were harvested at designated time points after exposure to 8 Gy of X-rays (210 kV, 1.045 Gy/min). Next, 10 μL trypsinized cells were mixed with molten LMA agarose and pipetted onto CometSlides. After incubation with Lysis Solution, the slides were transferred to a horizontal electrophoresis chamber for electrophoresis at 20 V for 25 min. Finally, DNA was stained with ethidium bromide (EB, Sigma) for 20 min in darkness. The images were captured by a fluorescence microscope (Olympus) and analysis was performed using CaspLab software.

### Senescence-associated beta-galactosidase (SA-β-gal) assay

SA-β-Gal staining was performed following the manufacturer’s protocol (Beyotime, Shanghai, China). After fixation with 4% formaldehyde for 10 min at room temperature, the cell samples were washed with PBS and subsequently incubated with freshly prepared SA-β-Gal staining solution overnight at 37 °C overnight. The next day, the cells were rinsed three times with PBS for 5 min each. Imaging was performed using a fluorescence microscope (Olympus, Tokyo, Japan), and the number of SA-β-Gal-positive cells was quantified in three randomly selected microscopic fields.

### Luciferase assay

HFF1 cells were seeded at a density of 5 × 10^5^ cells per well and cultured overnight. The cells were pretreated with 10 μM AQ 24 h prior to transfection with the luciferase reporter plasmids containing the promoter region of SIRT1. The cells were collected and lysed at 48 h post transfection, and luciferase activity was measured using the Dual-Luciferase Reporter Assay System (Promega, Madison, WI, USA) by the Tecan/Spark Luminometer (Austria). The luciferase activities were measured using the Dual-Luciferase Reporter Assay System (Beyotime) and normalized to Renilla luciferase.

### Immunoprecipitation

Anti-SIRT1 antibody (#ab189494) was purchased from Abcam (Cambridge, UK). For immunoprecipitation, HFF1 cells were lysed on ice using RIPA buffer and then centrifuged at 14,000 × *g* for 15 min at 4 °C. The supernatants were subjected to immunoprecipitation using antibody-conjugated Protein A/G Magnetic Beads (Bimake, TX, USA), which were incubated overnight at 4 °C. Immune complexes were extensively washed three times and analysed by western blotting with specific antibodies.

### Western blot

Cells were lysed with ice-cold lysis buffer containing protease and phosphatase inhibitors. Equal amounts of total protein were separated by SDS-PAGE, and electrically transferred to PVDF membranes (Millipore, Bedford, MA, USA). After blocking with 5% BSA, membranes were incubated with the following primary antibodies, all diluted to 1:1000: p21^WAF1^ (#37543), p16^ink4a^ (#18769), pDNA-PKcs (#68716), DNA-PKcs (#4602), Ligase IV (#14649), Ku80 (#2180), Ku70 (#4588), BRCA2 (#10741), BRCA1 (#14823), CtIP (#9201), Mre11 (#4895), Rad50 (#3427), pNBS1 (#3001), NBS1 (#14956), Rad54 (#15016), Rad51 (#8875), RPA2 (#35869), SIRT1 (#9475), SIRT2 (#12650), SIRT3 (#5490), SIRT6 (#12486), Histone Deacetylase (HDAC) Antibody Sampler Kit (#9928) and β-actin (#4967) (Cell Signaling Technology, Danvers, MA, USA). After washing, goat anti-rabbit or goat anti-mouse horseradish peroxidase-conjugated secondary antibodies (Beyotime) were added for incubation at room temperature for 1 h. Immunoblotting signals were detected using an enhanced chemiluminescence method.

### Reverse transcription‑polymerase chain reaction (RT‑PCR)

Total RNA was extracted using TRIzol Reagent (Invitrogen). Reverse transcription was performed using PrimeScript RT Reagent Kit (Takara) to obtain cDNA. RT-PCR amplifications were carried out by using UltraSYBR Mixture (CWBio, Beijing, China). Relative gene expression was measured by the 2^−ΔΔCt^ method and normalized with the endogenous reference gene (GAPDH). The target genes and their primer sequences are listed in Table 1.

**Table 1:** The primer sequences used for qRT-PCR.

Gene		Primer Sequence (5′-3′)
p21	Forward	GCACTCAGAGGAGGCGCCATGTCA
	Reverse	CTGTCCCCGCAGCAGAGCAGGT
p16	Forward	AGCCTTCGGCTGACTGGCTGG
	Reverse	GCGCTGCCCATCATCATGAC
IL1α	Forward	GGTTGAGTTTAAGCCAATCCA
	Reverse	TGCTGACCTAGGCTTGATGA
IL1β	Forward	CTGTCCTGCGTGTTGAAAGA
	Reverse	TTGGGTAATTTTTGGGATCTACA
IL8	Forward	AGACAGCAGAGCACACAAGC
	Reverse	ATGGTTCCTTCCGGTGGT
CXCL1	Forward	TCCTGCATCCCCCATAGTTA
	Reverse	CTTCAGGAACAGCCACCAGT
SIRT1	Forward	CAGATCCTCAAGCGATGTTT
	Reverse	CTGTTCCAGCGTGTCTATGTT
GAPDH	Forward	CATGAGAAGTATGACAACAGCCT
	Reverse	AGTCCTTCCACGATACCAAAGT

### Statistical analysis

All quantitative data are presented as mean ± standard deviation (SD) of at least three independent experiments. Statistical analyses were performed using Student’s t-test with SPSS 20.0 software (SPSS Inc., Chicago, IL). *P*-values < 0.05 were considered significant.

## Supplementary information

Original Data

Supplementary information file

## Data Availability

All data generated or analysed during this study are included in this published article [and its supplementary information files].
