# Supplementary figures and images for: Amodiaquine ameliorates stress-induced premature cellular senescence via promoting SIRT1-mediated HR repair
Source: Cell Death Discov. 2024 Oct 11;10:434. doi: 10.1038/s41420-024-02201-1 (PMC11470136; doi:10.1038/s41420-024-02201-1)

Figure 3E

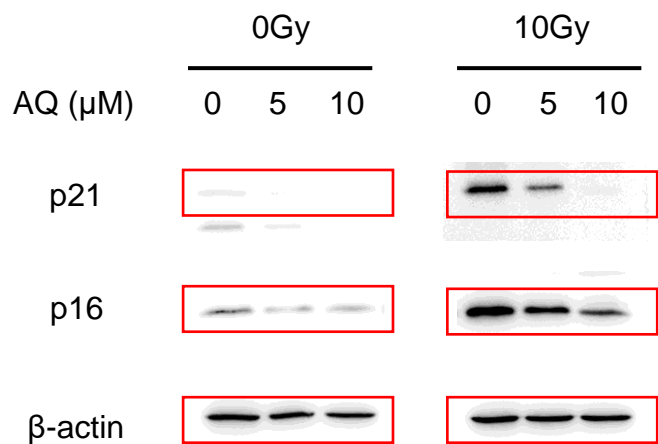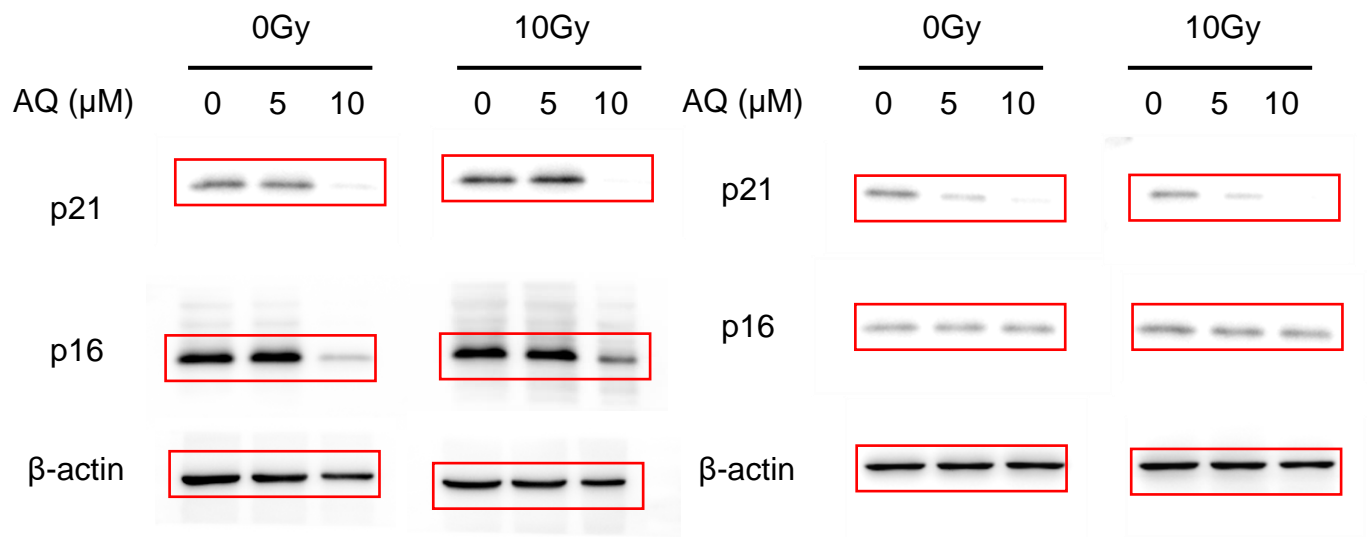

Figure 3H

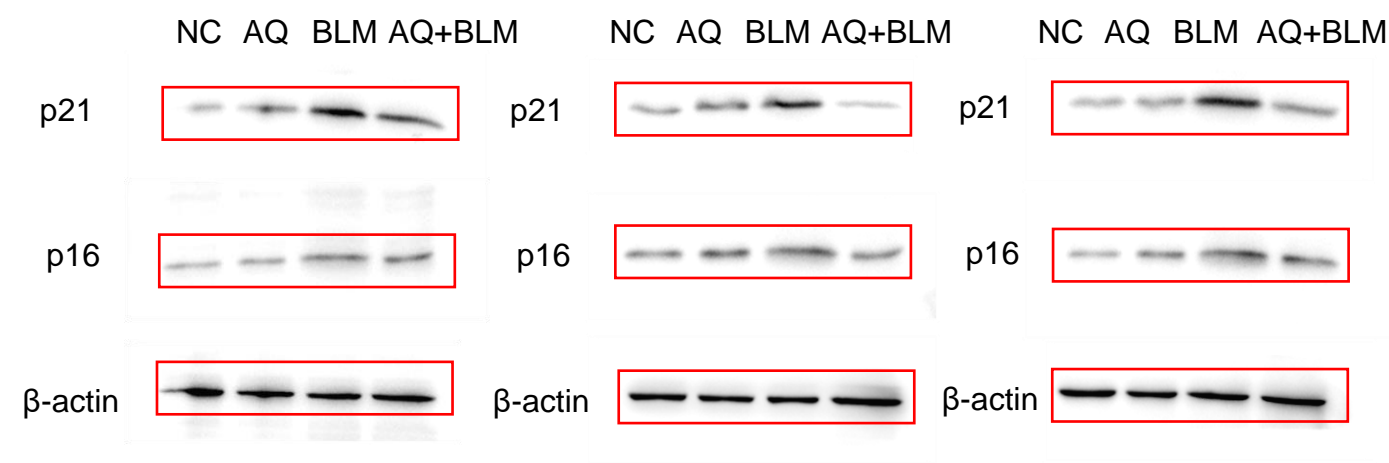

Figure 4A

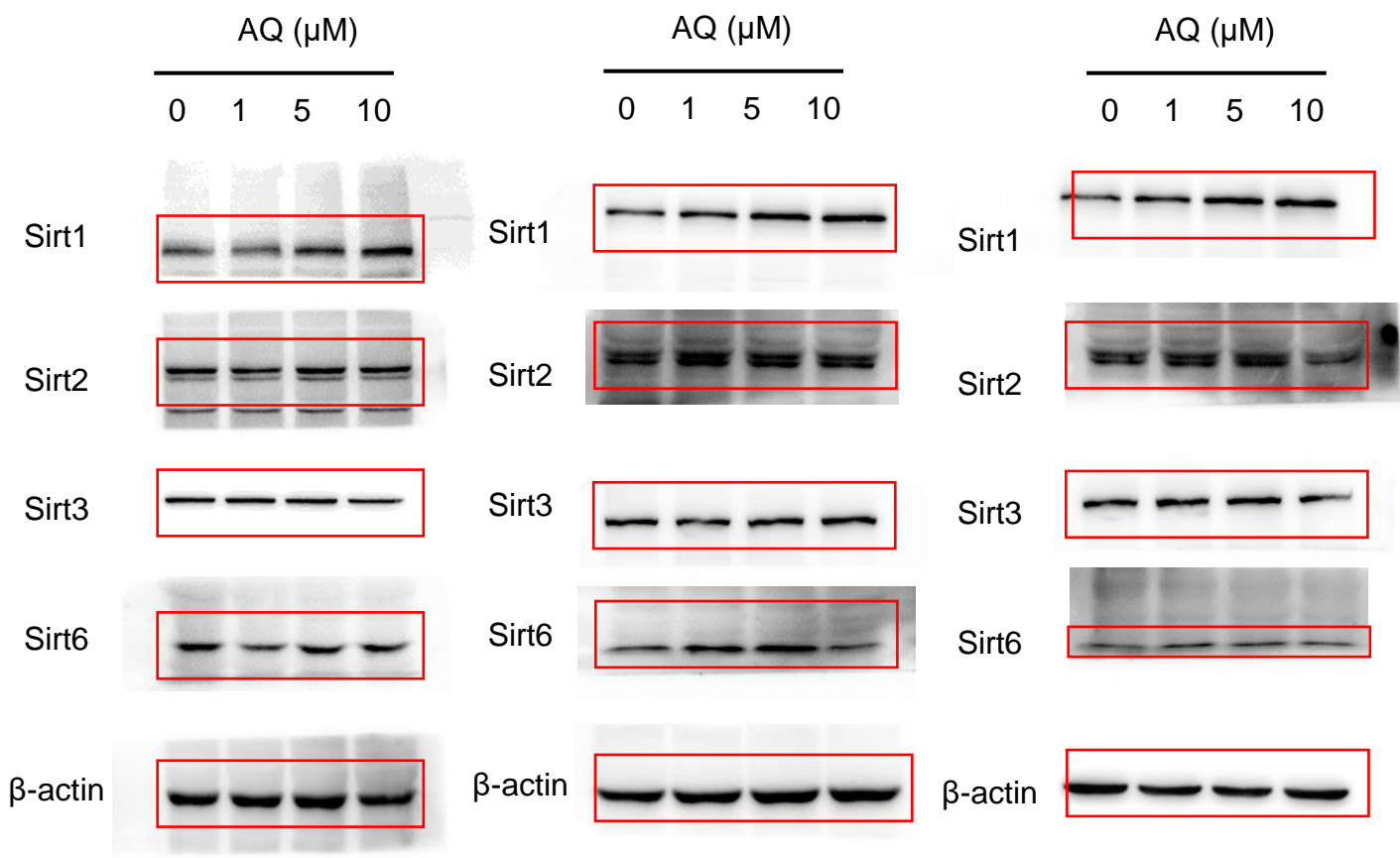

Figure 4E

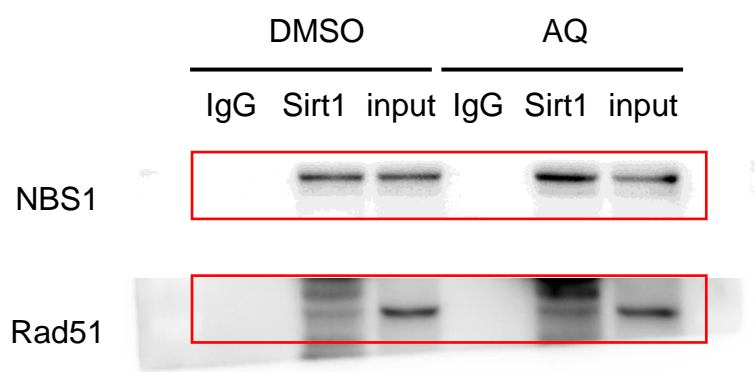

Figure 5A

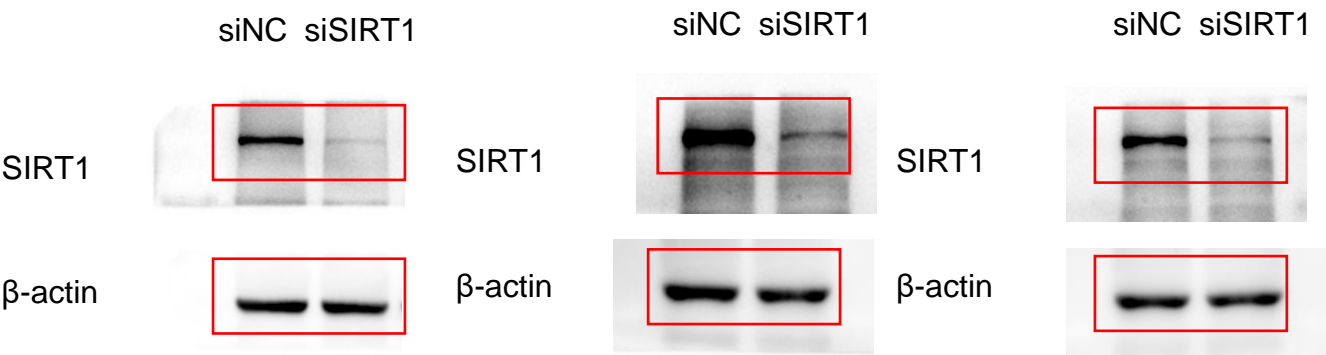

Figure 6C

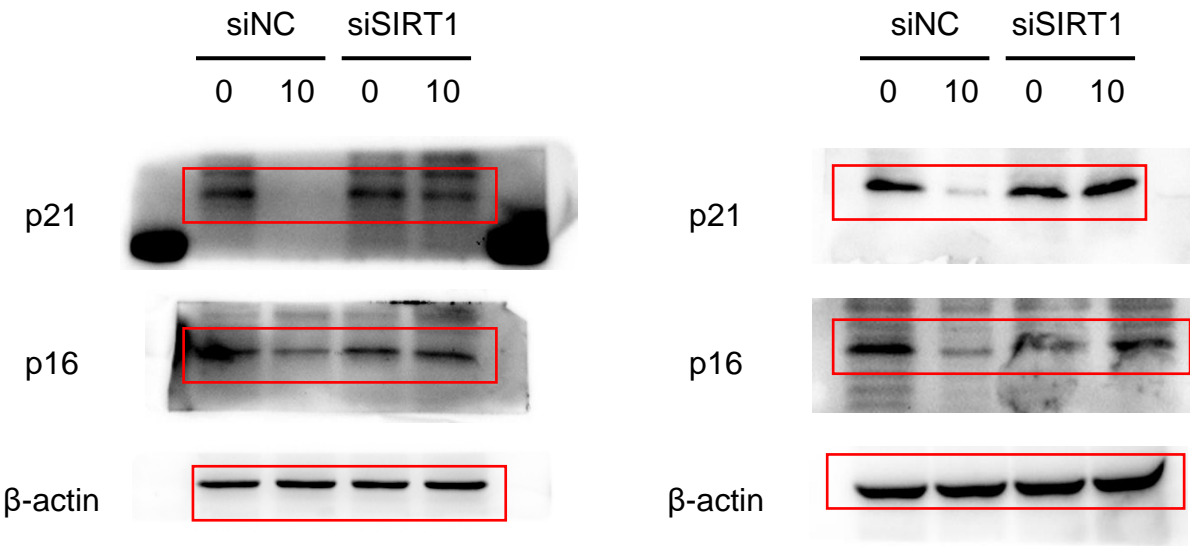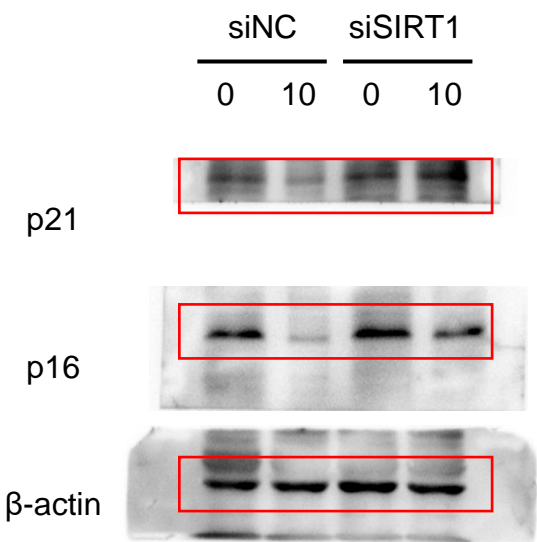

Figure S3A

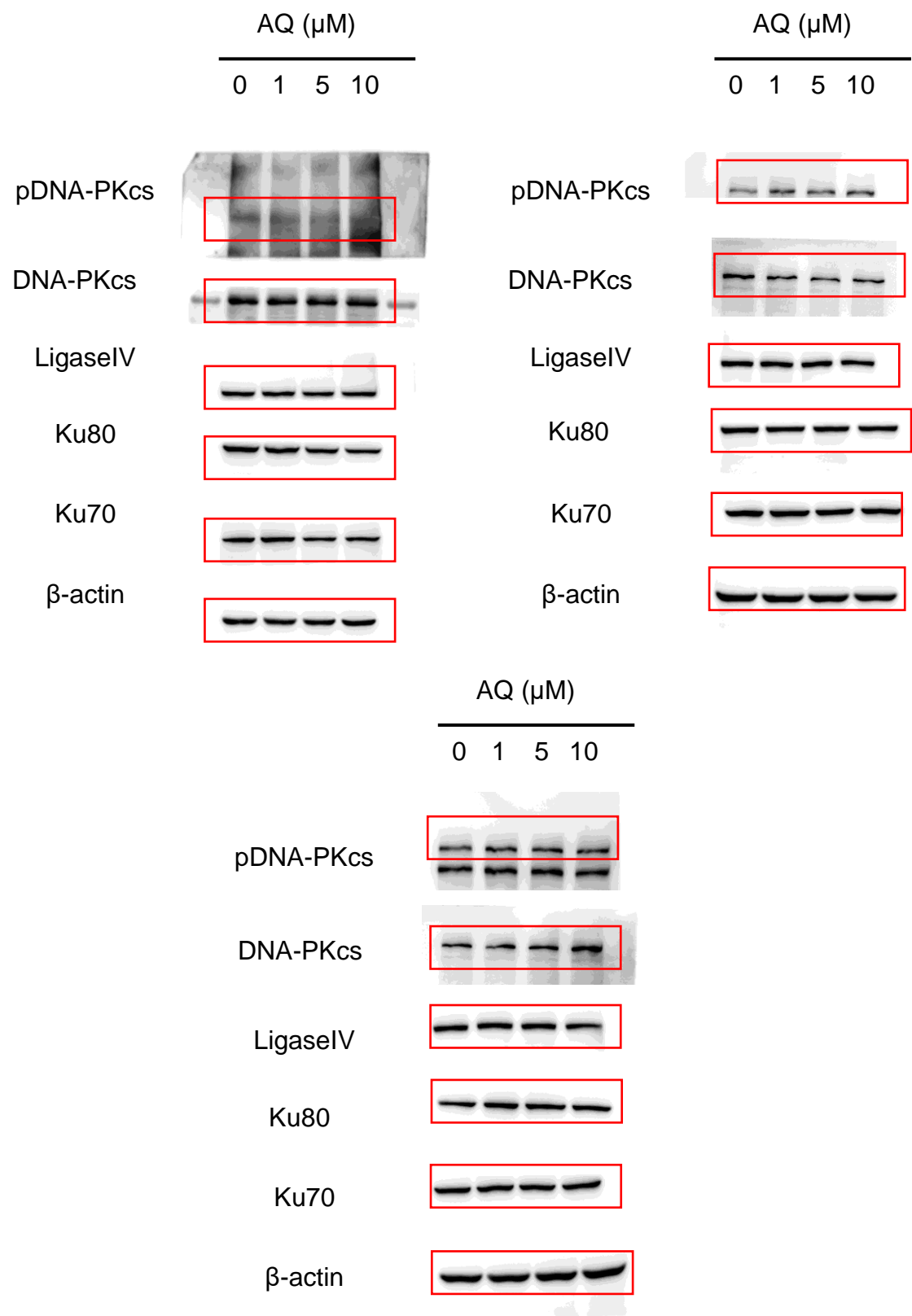

Figure S3B

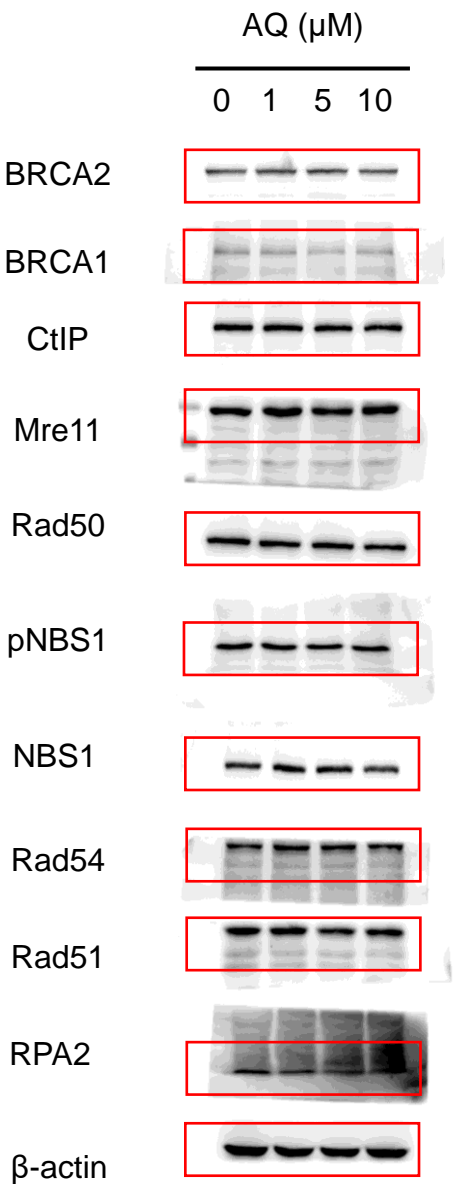

Figure S3B

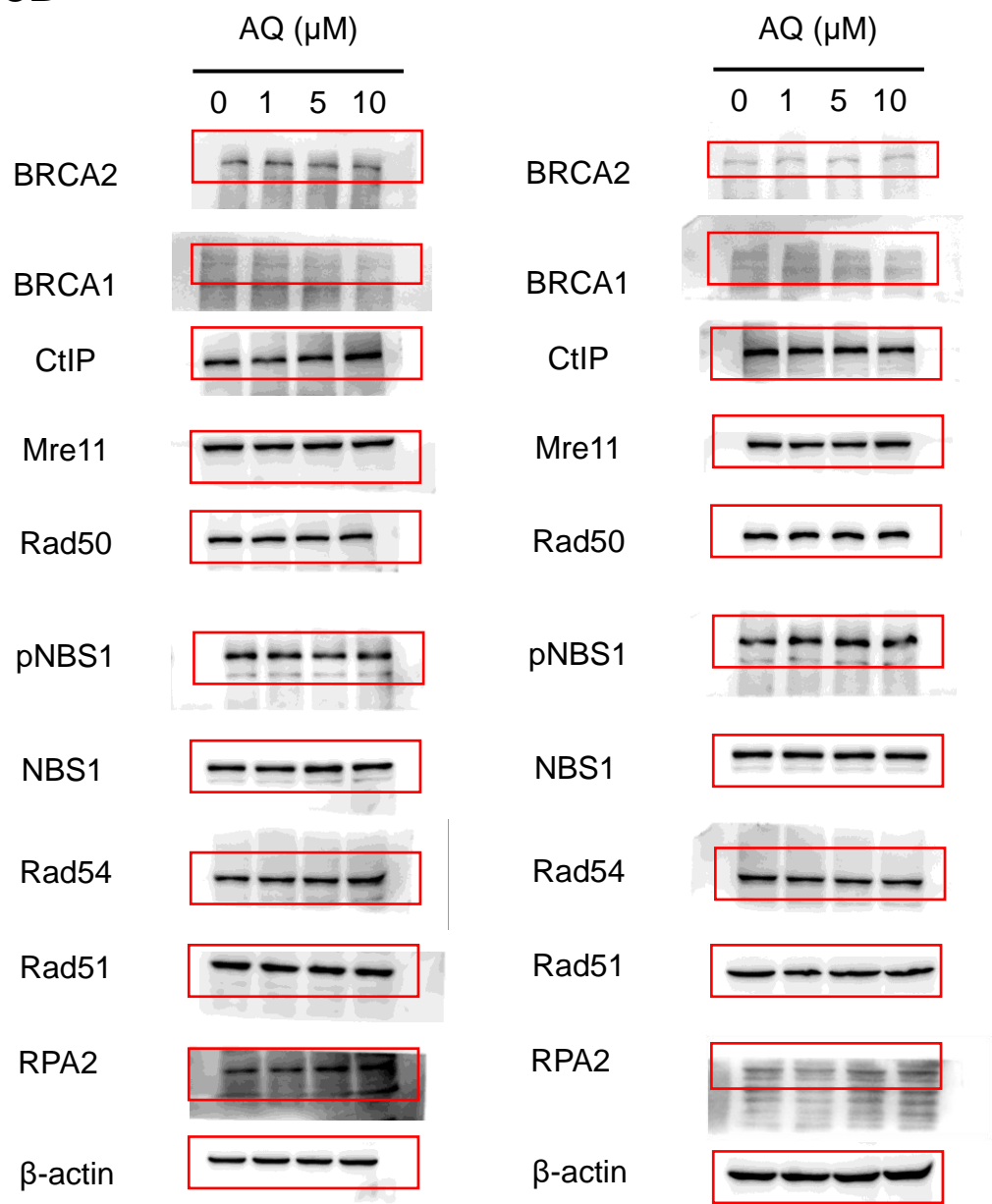

Figure S3C

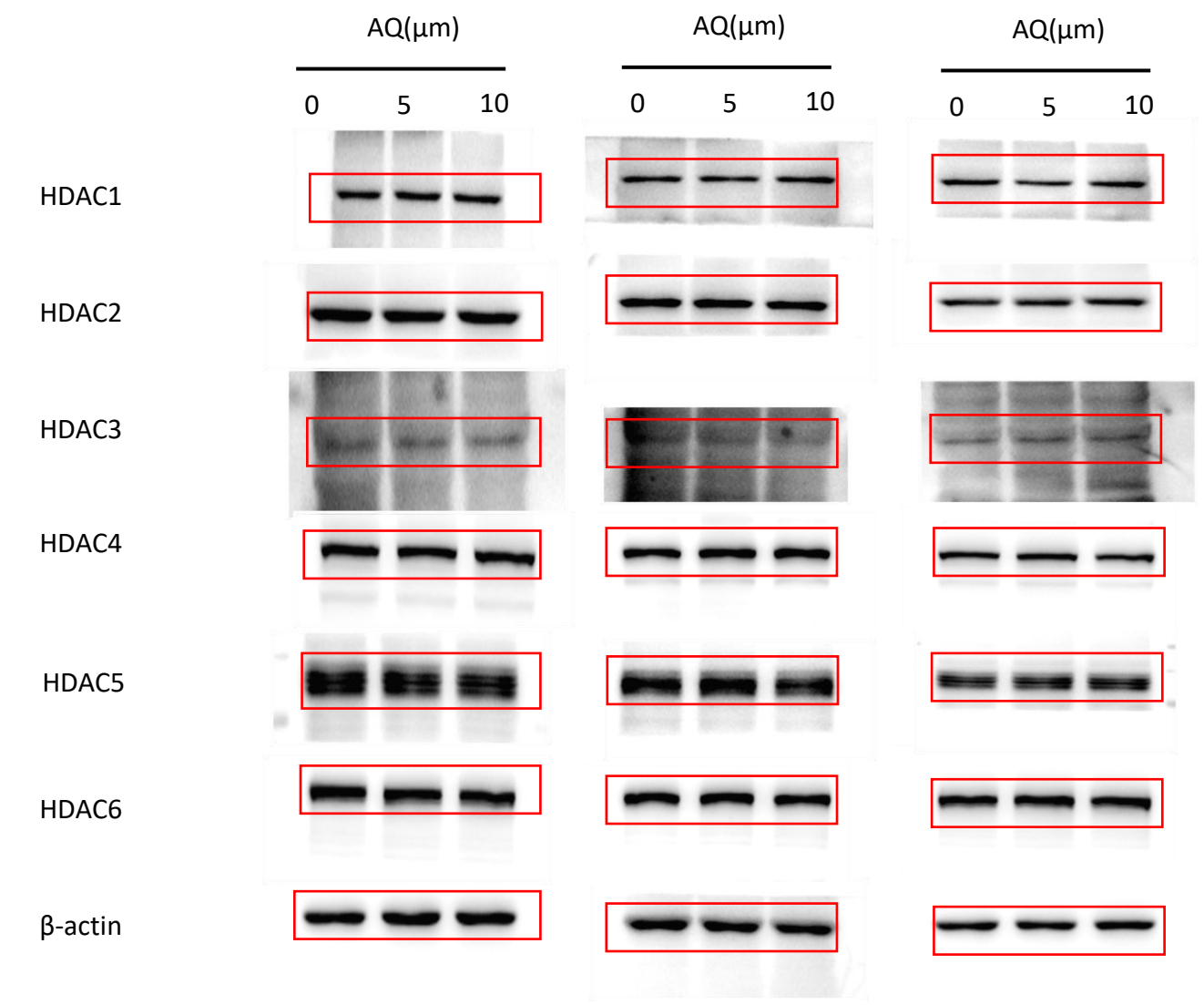

Supplement: Supplementary file 1 — Original Data [file 41420_2024_2201_MOESM1_ESM.pdf]
