# Supplementary material for: Amodiaquine ameliorates stress-induced premature cellular senescence via promoting SIRT1-mediated HR repair
Source: Cell Death Discov. 2024 Oct 11;10:434. doi: 10.1038/s41420-024-02201-1 (PMC11470136; doi:10.1038/s41420-024-02201-1)
Supplement: Supplementary file 2 — Supplementary information file [file 41420_2024_2201_MOESM2_ESM.pdf]

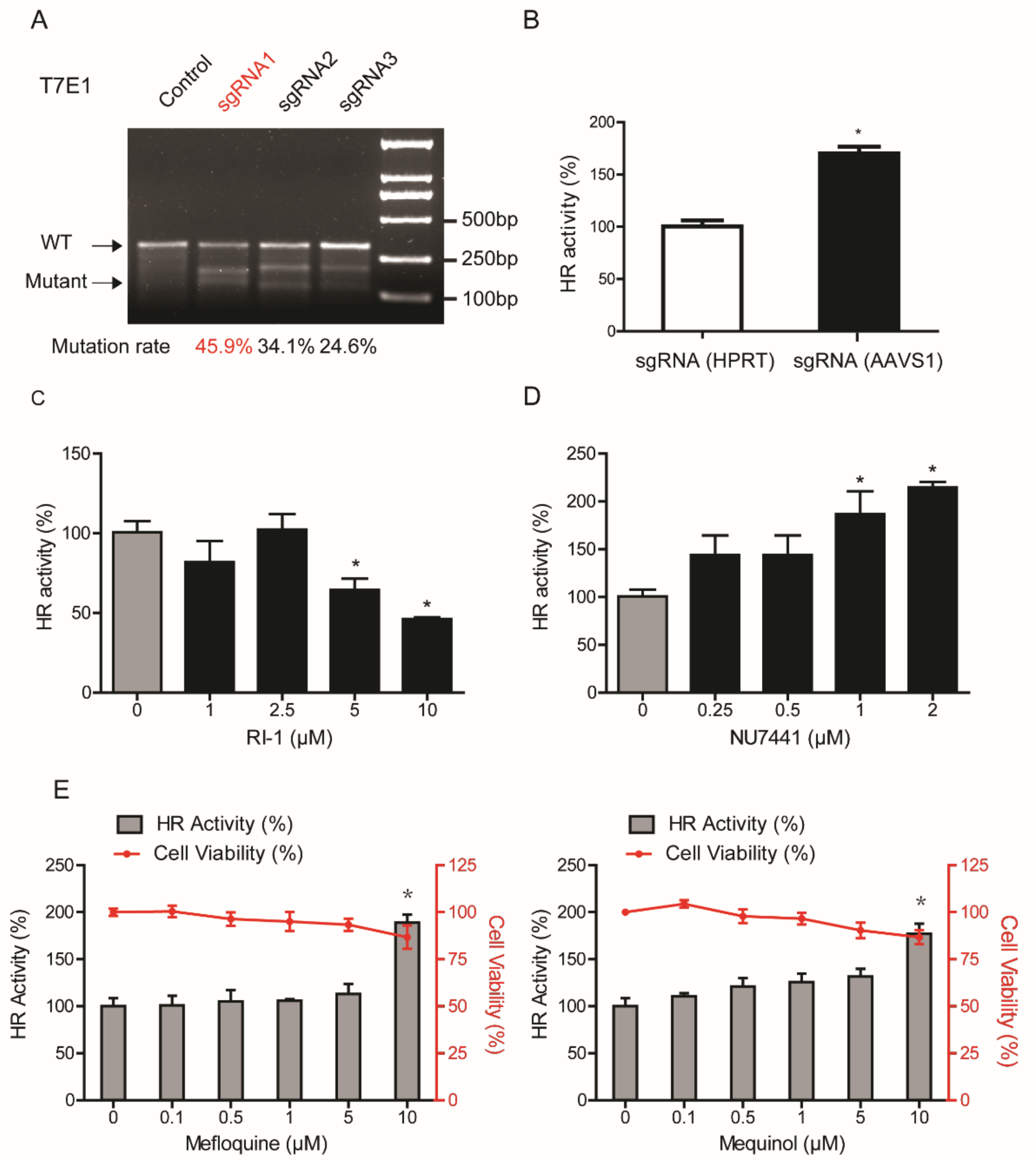

Figure S1

A. HEK293T-spCas9 cells were transfected with sgRNA targeting AAVS1 locus. Genomic DNA was extracted after 48 hours, followed by T7 Endonuclease I (T7E1)

cleavage assay to assess targeted mutations.

B. HEK293T-spCas9 cells were transfected with sgRNA targeting either the HPRT or AAVS1 locus along with dsDNA. 48 hours post-transfection, genomic DNA was extracted and subjected to qRT-PCR analysis to evaluate HR activity.

C, D. HEK293T-spCas9 cells were co-transfected with sgRNA and dsDNA, followed by treatment with different concentrations of RI-1 (C) or NU7441 (D). 48 hours post-transfection, genomic DNA was extracted and subjected to PCR analysis to confirm the feasibility of the screening workflow.

E, F. The cytotoxic and HR-promoting effects of mefloquine (E) and mequinol (F) were displayed in a dose-dependent manner. Significance markers: *, $p < 0.05$ compared to control; n=3.

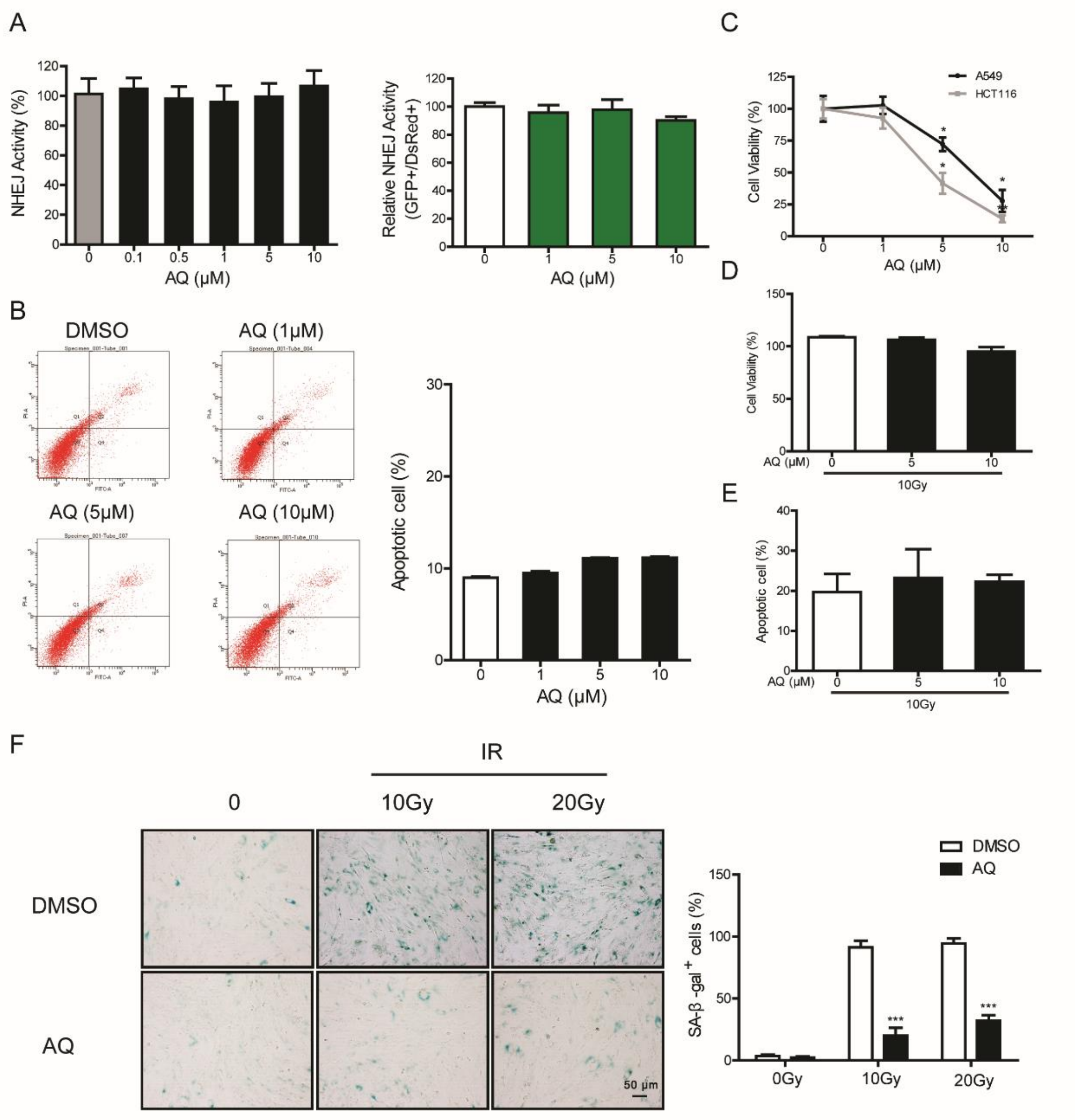

Figure S2

A. NHEJ activity was assessed using our established detecting assay and GFP-based NHEJ reporter assay.

B. HFF1 cells were harvested and evaluated by flow cytometry with Annexin V-FITC and PI staining after AQ treatment for 48 hours.

C. A549 and HCT116 cells were treated with AQ for 48 hours and cell viability was determined by CCK-8 assay.

D-F. HFF1 cells were pretreated with the indicated doses of AQ for 24 hours prior to X-ray exposure and further incubated for 7 days. The cell viability (D) and apoptosis rates (E) were determined, and the representative images and quantitative analyses of SA-β-Gal staining were shown (F). Significance markers: *, $p < 0.05$; **, $p < 0.01$; ***, $p < 0.001$ compared to control; n=3.

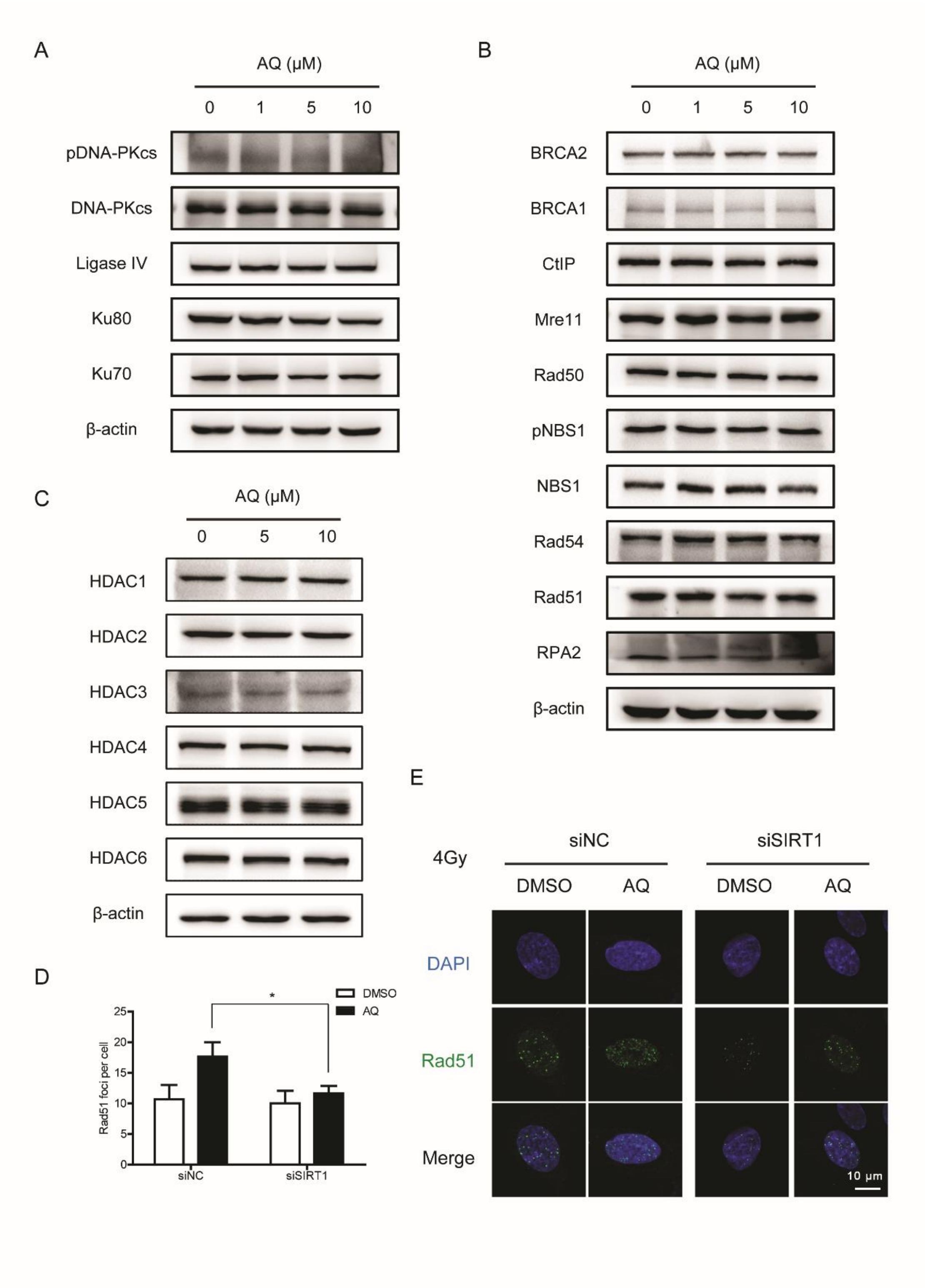

A
AQ (μM)
0
1
5
10
pDNA-PKcs
DNA-PKcs
Ligase IV
Ku80
Ku70
β-actin
B
AQ (μM)
0
1
5
10
BRCA2
BRCA1
CtIP
Mre11
Rad50
pNBS1
NBS1
Rad54
Rad51
RPA2
β-actin
C
AQ (μM)
0
5
10
HDAC1
HDAC2
HDAC3
HDAC4
HDAC5
HDAC6
β-actin
D
DMSO
AQ
*
Rad51 foci per cell
25
20
15
10
5
0
siNC
siSIRT1
E
4Gy
siNC
siSIRT1
DMSO
AQ
DMSO
AQ
DAPI
Rad51
Merge
10 μm

Figure S3

A, B. HFF1 cells were treated with the indicated concentrations of AQ for 48 hours, and the expression levels of NHEJ (A) and HR (B) pathway-associated factors were analyzed by Western blotting.

C. HFF1 cells were treated with AQ for 48 hours at indicated concentrations. The expression levels of HDAC1, HDAC2, HDAC3, HDAC4, HDAC5, and HDAC6 were determined by Western blotting.

D, E. The SIRT1 depleted cells were pretreated with AQ for 24 hours prior to 4 Gy of X-ray. Immunofluorescent staining for Rad51 foci (green) were performed 4 hours post-irradiation. The representative images (E) and the number of Rad51 foci per cell (D) were presented. Significance markers: *, $p < 0.05$ compared to control; n=3.

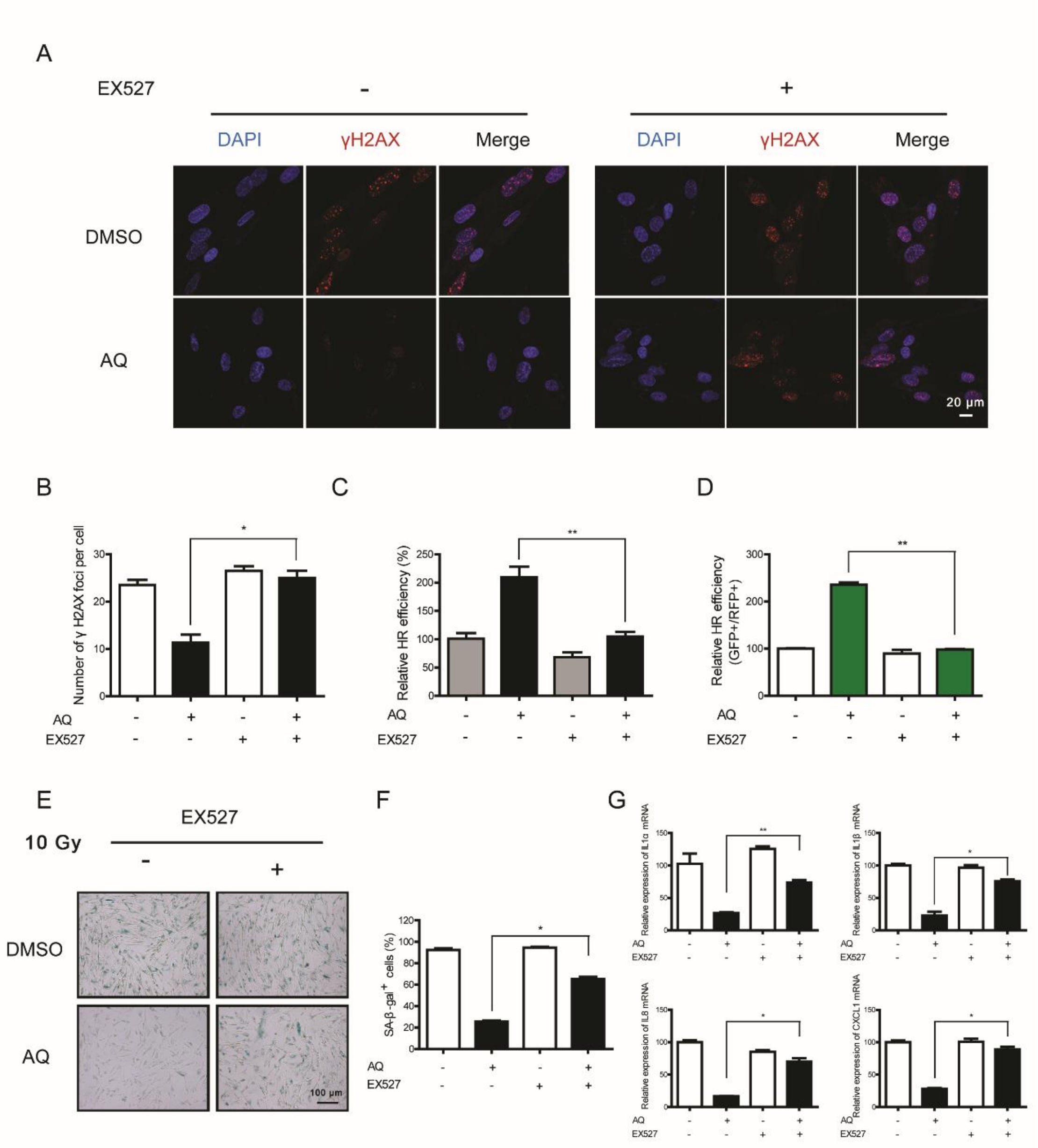

Figure S4

A, B. HFF1 cells were pretreated with AQ and EX527 alone or in combination for 24 hours, followed by irradiation with 4 Gy of X-rays. Immunofluorescent staining for

γH2AX foci (red) were performed 8 hours post-irradiation. The representative images (A) and the number of γH2AX foci per cell (B) were presented.

C, D. Treatment with EX527 abolished the AQ-mediated augmentation of HR repair. HR activity was assessed using our established detecting assay (C) and DR-GFP reporter assay (D).

E, F. HFF1 cells were pretreated with AQ and EX527 alone or in combination for 24 hours, followed by irradiation with 10 Gy X-rays. SA-β-Gal staining was performed 7 days post-irradiation.

G. Treatment with EX527 partially reversed the AQ-mediated suppression of SASP factors. Significance markers: *, $p < 0.05$; **, $p < 0.01$ compared to control; n=3.

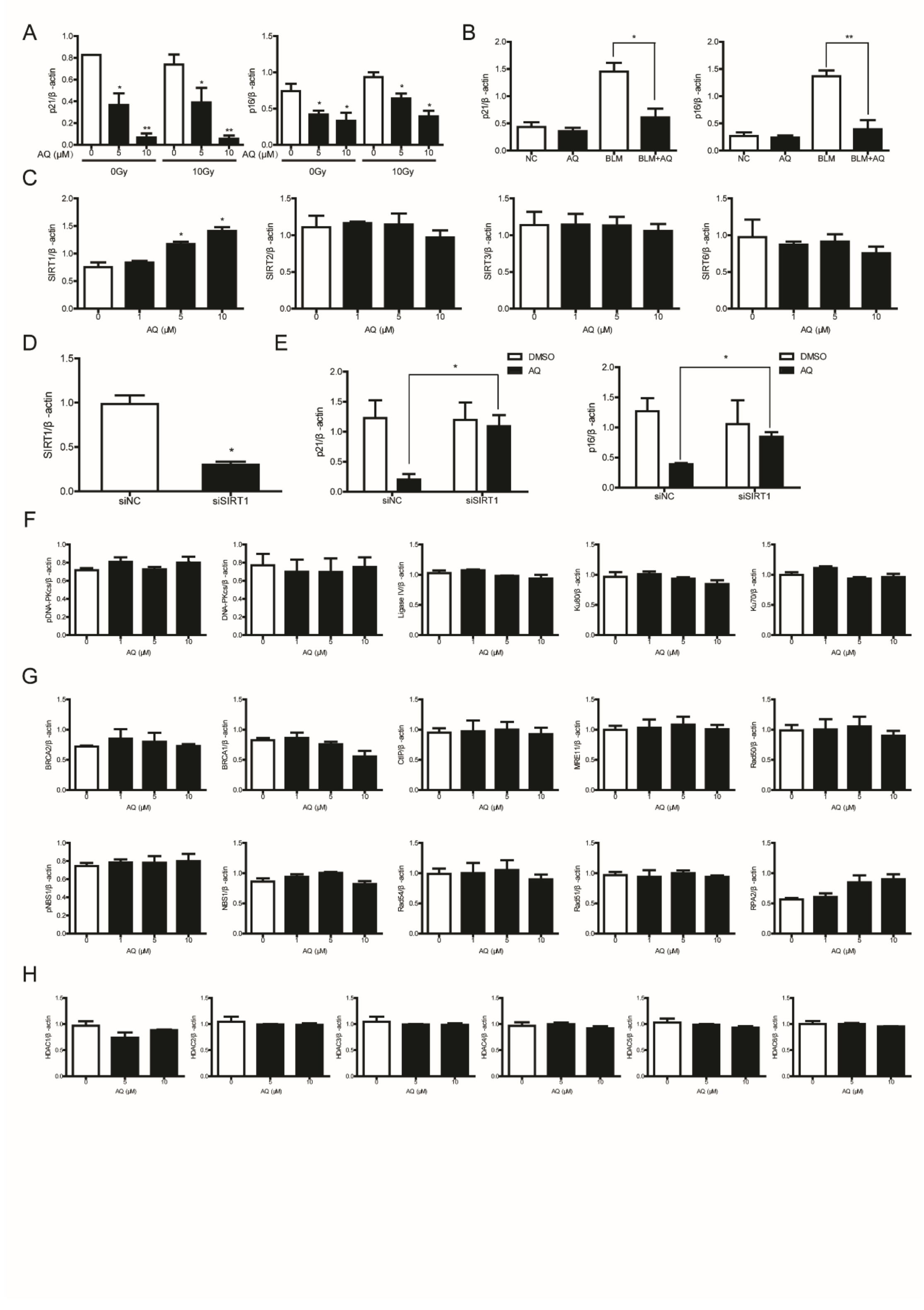

59

60 **Figure S5**

A-H. The quantification of WB results in Fig. 3E, Fig. 3H, Fig. 4A, Fig. 5A, Fig. 6C, Fig. S3A, Fig. S3B, and Fig. S3D was shown. Significance markers: *, $p < 0.05$; **, $p < 0.01$ compared to control; n=3.

Table S1. Full table of the screening results

A full list of the screening results of HR activity is shown.

Catalog Number	CAS Number	Product Name	Synonyms	Indication	HR activity(%)
S2114	141625-93-6	Dronedarone HCl	SR33589	Neurological Disease	**258.53**
S1148	114977-28-5	Docetaxel	RP56976, NSC 628503	Cytoskeletal Signaling	**247.78**
S1579	827022-33-3	Palbociclib (PD0332991) Isethionate	N/A	Cancer	**245.91**
S1386	82956-11-4	Nafamostat Mesylate	FUT-175	Cardiovascular Disease	**245.52**
S5500	69-44-3	Amodiaquine hydrochloride	N/A	Immunology	**244.25**
S1625	57-63-6	Ethinyl Estradiol	N/A	Endocrinology	**243.99**
S4120	61318-91-0	Sulconazole Nitrate	N/A	Infection	**243.01**
S4107	2030-63-9	Clofazimine	NSC-141046	Infection	**241.42**
S1096	875320-31-3	Quisinostat (JNJ-26481585) 2HCl	N/A	Cancer	**241.37**
S2741	1038915-60-4	Niraparib (MK-4827)		Tacrine, Tetrahydroaminacrine, Tetrahydroaminoacridine	**239.92**
S7646	934493-76-2	Voxtalisib (XL765,	N/A	Cancer	**238.68**
S1208	25316-40-9	Doxorubicin (Adriamycin) HCl	NSC 123127	Cancer	**236.13**
S4610	31431-39-7	Mebendazole	Vermox, Telmin, Pantelmin, Mebenvet	Infection	**234.92**
S1238	10540-29-1	Tamoxifen	ICI 46474	10540-29-1	**234.17**
S4502	496775-61-2	Eltrombopag	N/A	Others	**233.94**
S1122	726169-73-9	Mocetinostat (MGCD0103)	MG0103	Cancer	**231.46**
S4420	51773-92-3	Mefloquine HCl	N/A	Infection	**228.39**
S1040	475207-59-1	Sorafenib Tosylate	Bay 43-9006	Cancer	**227.37**
S4077	150-76-5	Mequinol	N/A	Infection	**226.34**
S1640	54965-21-8	Albendazole	SKF-62979	Infection	**222.01**
S2803	851983-85-2	Galeterone	TOK-001	Cancer/Endocrinology	**214.94**
S1735	19767-45-4	Mesna	N/A	Cancer	**211.91**
S1342	446-72-0	Genistein	N/A	Cancer	**209.97**
S1003	796967-16-3	Linifanib (ABT-869)	AL39324,RG3635	Cancer	**206.52**
S4869	4563-84-2	Sulfamethoxazole sodium	N/A	Infection	**205.07**
S4617	6700-34-1	Dextromethorphan hydrobromide hydrate	Dextromethorphan hydrobromide monohydrate	Neurological Disease/Respiratory Disease	**201.12**
S3120	1229-29-4	Doxepin HCl	N/A	Neurological Diseases	**200.70**
S4217	55-06-1	Liothyronine Sodium	N/A	Endocrinology	**198.87**
S4634	547-32-0	Sodium sulfadiazine	Sulfadiazin-natrium	Infection	**198.45**
S2480	34552-83-5	Loperamide HCl	ADL 2-1294	Neurological Disease	**198.08**
S5054	14897-39-3	Rifamycin sodium salt		Others	**196.71**
S4716	314-13-6	Evans Blue	Direct Blue 53	Neuronal Signaling	**196.05**

S4053	79559-97-0	Sertraline HCl	CP-51974-1 HCl	Inflammation	**194.21**
S2128	198480-56-7	Bazedoxifene HCl	TSE-424	PF-05208749 HCl	**192.71**
S2535	24169-02-6	Econazole nitrate	NSC 243115	Neurological Disease	**191.74**
S4632	70-30-4	Hexachlorophene	Hexachlorofen, Fostril, Nabac, pHisoHex	Infection	**190.20**
S5506	508233-74-7	Vortioxetine	N/A	Neuronal Signaling	**190.04**
S4631	84-02-6	Prochlorperazine dimaleate salt	Prochlorperazin, Compazine, Capazine, Stemetil	Neurological Disease	**189.92**
S4553	52-51-7	Bronopol	N/A	Infection	**189.91**
S2851	1187594-09-7	Baricitinib (LY3009104, INCB028050)	N/A	Immunology	**189.43**
S5440	633-66-9	Berberine Sulfate	Umbellatine Sulfate	Microbiology	**188.96**
S7077	199807-35-7	Cilengitide?trifluo roacetate	EMD 121974, NSC 707544	Cancer	**188.03**
S1014	380843-75-4	Bosutinib (SKI-606)	N/A	Cancer	**187.30**
S1727	797-63-7	Levonorgestrel	N/A	Endocrinology	**186.85**
S4096	548-73-2	Droperidol	NSC 169874	Neurological Disease	**186.68**
S5350	87239-81-4	Cefpodoxime proxetil	N/A	Microbiology	**186.37**
S1368	55079-83-9	Acitretin	Etretin, RO 10-1670	Metabolic Disease	**185.72**
S4210	94-09-7	Benzocaine	N/A	Transmembrane Transporters	**185.68**
S3005	78246-49-8	Paroxetine HCl	BRL-29060A, FG-7051	Neurological Disease	**185.47**
S1021	302962-49-8	Dasatinib	BMS-354825	Cancer	**183.64**
S4637	389574-19-0	Prasugrel Hydrochloride	Prasugrel HCl, LY 640315	Cardiovascular Disease	**183.30**
S1550	74150-27-9	Pimobendan	UD-CG 115 BS	Cardiovascular Disease	**182.92**
S3079	95233-18-4	Atovaquone	Atavaquone	Neurological Disease	**182.69**
S1830	53716-50-0	Oxfendazole	RS-8858	Infection	**182.13**
S5563	130-61-0	Thioridazine hydrochloride	Aldazine, Mellaril	Others	**182.09**
S1369	859212-16-1	Bafetinib (INNO-406)	NS-187	Cancer	**182.06**
S4854	845533-86-0	Bedaquiline fumarate	TMC-207	Infection	**182.04**
S5498	54856-23-4	Betahistine mesylate	N/A	Neuronal Signaling	**181.30**
S1704	143491-57-0	Emtricitabine	N/A	Infection	**180.86**
S1286	51333-22-3	Budesonide	N/A	Endocrinology	**180.43**
S3701	57-37-4	Benactyzine hydrochloride	N/A	Neurological Diseases	**180.34**
S2048	24729-96-2	Clindamycin Phosphate	NSC 618653	Infection	**177.97**
S1373	103060-53-3	Daptomycin	LY146032	Infection	**177.72**
S1712	201530-41-8	Deferasirox	CGP-72670, ICL-670	Endocrinology	**177.67**
S1430	61413-54-5	Rolipram	ZK-62711, SB 95952	Neurological Disease/Immunology	**177.66**

S4633	16051-77-7	Isosorbide Mononitrate	Isosorbide-5-mononitrate, Elantan, Monoket, Mononit, Imdur, Corangin	Isosorbide-5-mononitrate, Elantan, Monoket, Mononit, Imdur, Corangin	**177.42**
S1792	79902-63-9	Simvastatin	MK-0733	Cardiovascular Disease	**176.86**
S4593	302-22-7	Chlormadinone acetate	Gestafortin	Endocrinology	**176.64**
S2208	566-48-3	Formestane	CGP-32349, NSC 282175	Caner/Endocrinology	**176.63**
S1090	783355-60-2	Abexinostat (PCI-24781)	CRA-024781	Cancer	**175.79**
S1409	81403-68-1	Alfuzosin HCl	N/A	Cardiovascular Disease	**175.66**
S2755	845272-21-1	Varlitinib	ARRY334543	Cancer	**175.60**
S4216	133868-46-9	Valnemulin HCl	N/A	Infection	**175.10**
S1322	50-02-2	Dexamethasone (DHAP)	N/A	Inflammation	**175.00**
S5416	54-36-4	Metyrapone	2-methyl-1,2-di-3-pyridyl-1-propanone; 2-methyl-1,2-dipyridin-3-ylpropan-1-one;	Metabolic Disease	**174.96**
S1832	64228-81-5	Atracurium Besylate	BW 33A, 51W89	Neurological Disease	**174.61**
S1085	414864-00-9	Belinostat (PXD101)	NSC726630, PX-105684	Cancer	**174.52**
S4203	3759-92-0	Furaltadone HCl	N/A	Infection	**173.80**
S2217	136572-09-3	Irinotecan HCl Trihydrate	CPT-11 HCl Trihydrate	Neurological Disease	**173.72**
S1144	873054-44-5	Ivacaftor (VX-770)	N/A	Respiratory disease	**173.40**
S2287	485-35-8	Cytisine	Baphitoxine,Sophorine	Neuronal Signaling	**173.19**
S3664	51529-01-2	Flupenthixol dihydrochloride	N/A	Neurological Disease	**172.58**
S1194	1012054-59-9	CUDC-101	N/A	Cancer	**172.44**
S3713	113507-06-5	Moxidectin	milbemycin B	Microbiology	**171.67**
S4525	120-47-8	Ethylparaben	Ethyl parahydroxybenzoate, Ethyl 4-hydroxybenzoate	Infection	**171.00**
S4576	127-71-9	Sulfabenzamide	Sultrin, N-Sulfanilylbenzamide	Infection	**170.85**
S5295	7179-49-9	Lincomycin Hydrochloride Monohydrate	N/A	Microbiology	**170.79**
S2293	461-05-2	DL-Carnitine HCl	N/A	Cardiovascular Disease	**170.69**
S4877	56839-43-1	Eperisone hydrochloride	N/A	Others	**170.51**
S8136	127373-66-4	Sivelestat (ONO-5046)	ONO5046, LY544349, EI546	Proteases	**169.86**
S2727	1110813-31-4	Dacomitinib (PF299804, PF299)	N/A	Cancer	**169.76**
S1567	19171-19-8	Pomalidomide	CC-4047	Cancer	**169.71**

S2240	286930-03-8	Fesoterodine Fumarate	SPM 907	Immunology	**169.64**
S1042	341031-54-7	Sunitinib Malate	N/A	SU-11248	**169.20**
S7059	1152311-62-0	Tezacaftor(VX-661)	N/A	Transmembrane Transporters	**169.14**
S1878	82410-32-0	Ganciclovir	RS-21592, BW-759	Infection	**169.06**
S1153	186692-46-6	Roscovitine (Seliciclib,CYC202)	N/A	Cancer	**168.18**
S4855	864821-90-9	Eluxadoline	JNJ-27018966	enteric nervous system	**168.06**
S1356	102767-28-2	Levetiracetam	UCB-L059, SIB-S1	Neurological Disease	**167.69**
S4635	41354-29-4	Cyproheptadine hydrochloride	Periactin hydrochloride, Peritol	Inflammation	**167.56**
S4620	56238-63-2	Cefuroxime sodium	N/A	Infection	**167.37**
S4151	26864-56-2	Penfluridol	TLP-607	Neurological Disease	**166.80**
S1811	2016-88-8	Amiloride HCl	N/A	Midamor, Colectril, Amipramizide, Guanamprazine hydrochloride	**166.52**
S5042	42864-78-8	Bevantolol hydrochloride	NC-1400 hydrochloride	Neuronal Signaling	**166.43**
S1033	641571-10-0	Nilotinib (AMN-107)	N/A	Cancer	**166.38**
S1401	147127-20-6	Tenofovir	GS-1278	Infection	**166.10**
S2552	79307-93-0	Azelastine HCl	N/A	Neurological Disease	**165.55**
S3207	73334-07-3	Iopromide	N/A	Diagnosis	**165.21**
S1201	16208-51-8	Dimesna	BNP-7787	Cancer	**164.77**
S3114	50-81-7	Vitamin C	Ascorbic acid	Respiratory Disease	**164.56**
S5716	1231929-97-7	Abemaciclib	LY2835219	Cell Cycle	**163.91**
S8001	1316214-52-4	Ricolinostat (ACY-1215)	Rocilinostat	Cancer	**163.67**
S4558	1195-16-0	Citiolone	N/A	Neurological Disease	**163.23**
S3081	159811-51-5	Ulipristal	CDB-2914	Endocrinology	**162.74**
S1472	202825-46-5	Safinamide Mesylate	PNU-151774E,FCE28073	Neurological Disease	**162.19**
S3154	101827-46-7	Butenafine HCl	KP-363	Neurological Disease	**162.13**
S1723	53-86-1	Indomethacin	N/A	Inflammation	**161.80**
S1884	110871-86-8	Sparfloxacin	AT-4140,CI-978,PD 131501	Infection	**161.38**
S1390	99614-01-4	Ondansetron HCl	GR 38032F	Ondemet, Emeset, Emetron	**160.91**
S3195	37091-65-9	Azlocillin sodium salt	N/A	Neurological Disease	**160.58**
S1665	53-16-7	Estrone	N/A	Endocrinology	**160.51**
S1733	83-43-2	Methylprednisolone	NSC-19987	Immunology	**159.75**

S8432	97322-87-7	Troglitazone (CS-045)	Rezulin, Romglizone, Prelay, CS-045, CS045, CS 045, Romozin	Metabolic Disease	**159.66**
S4526	36330-85-5	Fenbufen	Lederfen, CL-82204	Inflammation	**159.49**
S4896	832720-36-2	Elagolix Sodium	NBI-56418, ABT-620	Endocrinology & Hormones	**159.19**
S2470	67-73-2	Fluocinolone Acetonide	Flucort-N	Inflammation	**158.93**
S5494	18559-94-9	Salbutamol	Albuterol; AH-3365	GPCR	**158.66**
S3147	130929-57-6	Entacapone	OR-611	Neurological Disease	**158.65**
S1048	639089-54-6	Tozasertib (VX-680, MK-0457)	N/A	Cancer	**158.49**
S1302	3778-73-2	Ifosfamide	NSC109724, Isophosphamide	Cancer	**158.15**
S4201	73231-34-2	Florfenicol	SCH-25298	Infection	**157.78**
S1629	96829-58-2	Orlistat	Ro 18-0647, Tetrahydrolipstatin	Metabolic Disease	**157.71**
S1836	54029-12-8	Albendazole Oxide	Ricobendazole	Infection	**157.41**
S5297	24356-66-9	Vidarabine monohydrate	Spongoadenosine monohydrate, Vira-A monohydrate	DNA Damage	**157.33**
S4227	873857-62-6	Fidaxomicin	OPT-80, PAR-101	Infection	**157.25**
S5234	656247-18-6	Nintedanib Ethanesulfonate Salt	Intedanib, BIBF 1120	Protein Tyrosine Kinase	**157.05**
S1784	5536-17-4	Vidarabine	N/A	Infection	**156.67**
S2541	17321-77-6	Clomipramine HCl	N/A	Neurological Disease	**156.20**
S4546	87-99-0	Xylitol	Adonitol, Ribitol, Xylite, D-Xylitol, Adonite	Others	**156.17**
S3007	139110-80-8	Zanamivir	GG167	Infection	**155.78**
S4297	12650-69-0	Mupirocin	N/A	Infection	**155.74**
S1147	722544-51-6	Barasertib (AZD1152-	INH 34	Cancer	**155.68**
S5534	85-79-0	Cinchocaine	Dibucaine	Others	**155.67**
S3054	5560-59-8	Alverine Citrate	NSC 35459	Gastroenterology	**155.65**
S1739	148-79-8	Thiabendazole	N/A	Infection	**155.59**
S3172	58579-51-4	Anagrelide HCl	BL-4162A	Endocrinology	**155.38**
S5066	104632-25-9	Pramipexole dihydrochloride	N/A	Neuronal Signaling	**154.87**
S4548	1937-19-5	Aminoguanidine hydrochloride	Pimagedine, Guanyl hydrazine, Hydrazinecarboximidamide, Imino semicarbazide, Monoaminoguanidine	Metabolic Disease	**154.76**
S4296	94749-08-3	Salmeterol Xinafoate	N/A	Respiratory Disease	**154.69**

S4589	6398-98-7	Amodiaquine dihydrochloride dihydrate	N/A	Infection/Inflammation	**154.65**
S1763	111974-72-2	Quetiapine Fumarate	ICI-204636	Neurological Disease	**154.53**
S1064	790299-79-5	Masitinib (AB1010)	N/A	Cancer/Immunology	**154.38**
S5733	57-11-4	Stearic acid		Others	**154.08**
S5256	19428-14-9	Benproperine phosphate	Blascorid, Pirexyl phosphate	Others	**154.02**
S3133	57-68-1	Sulfamethazine	N/A	Endocrinology	**153.83**
S1761	40828-46-4	Suprofen	N/A	Inflammation	**153.78**
S4846	119478-56-7	Meropenem Trihydrate	N/A	Microbiology	**153.69**
S2373	65-19-0	Yohimbine HCl	Antagonil	Others	**153.58**
S2067	78712-43-3	Ozagrel HCl	OKY-046 HCl	Metabolism	**153.41**
S1101	212141-51-0	Vatalanib (PTK787) 2HCl	ZK 222584 (cpg-79787) 2HCl	Cancer	**153.18**
S4878	130773-02-3	Neticonazole Hydrochloride	N/A	Infection	**153.14**
S2807	1195765-45-7	Dabrafenib (GSK2118436)	N/A	Cancer	**153.14**
S1802	2627-69-2	AICAR (Acadesine)	NSC105823	Cardiovascular Disease	**153.08**
S3639	206658-92-6	Tacrine hydrochloride hydrate	Tacrine, Tetrahydroaminacrine, Tetrahydroaminoacridine	Neuronal Signaling	**153.01**
S1490	943319-70-8	Ponatinib (AP24534)	N/A	Cancer	**152.80**
S8041	934660-93-2	Cobimetinib (GDC-0973, RG7420)	XL518	Cancer	**152.56**
S1540	361442-04-8	Saxagliptin	BMS-477118	Proteases	**152.50**
S5404	1216941-48-8	Paritaprevir (ABT-450)	N/A	Proteases	**152.42**
S7782	863127-77-9	Dasatinib Monohydrate	BMS-354825 Monohydrate	BMS-354825 Monohydrate	**152.13**
S1702	69655-05-6	Didanosine	N/A	Infection	**151.97**
S1764	13292-46-1	Rifampin	Rimactane	Infection	**151.79**
S3075	113775-47-6	Dexmedetomidine	N/A	Neurological Disease	**151.68**
S5650	9067-32-7	Sodium Hyaluronate		Others	**151.62**
S3021	168273-06-1	Rimonabant	SR141716	Metabolic Disease	**151.30**
S4657	236395-14-5	Eslicarbazepine Acetate	BIA 2093, Zebinix, Exalief, Stedesa, Aptiom	Neurological Disease	**151.17**
S5062	1009119-65-6	Daclatasvir Digydrochloride	N/A	Infection	**151.10**
S1760	61379-65-5	Rifapentine	MDL473	Infection	**151.06**
S7262	717824-30-1	Vidofludimus	SC12267, 4SC-101	Immunology	**151.05**
S2792	262352-17-0	Torcetrapib	CP-529414	Metabolic Disease	**150.84**
S4163	24390-14-5	Doxycycline Hyclate	N/A	Proteases	**150.74**
S1252	209216-23-9	Hydrate	N/A	Infection	**150.73**
S5730	4330-99-8	Alimemazine Tartrate		Others	**150.65**

S4709	130209-82-4	Latanoprost	Xalatan, PhXA41, PHXA-41	Immunology & Inflammation	**150.56**
S4289	7232-21-5	Metoclopramide HCl	N/A	Neurological Disease	**150.44**
S1223	56390-09-1	Epirubicin HCl	4'-epidoxorubicin HCl	Cancer	**150.41**
S3694	66-84-2	Glucosamine hydrochloride	2-Amino-2-deoxy-glucose HCl, Chitosamine HCl	Others	**150.38**
S1736	532-03-6	Methocarbamol	AHR 85	Neurological Disease	**150.29**
S7397	284461-73-0	Sorafenib	BAY 43-9006	BAY 43-9006	**149.92**
S4935	630420-16-5	Asunaprevir	BMS-650032	Proteases	**149.75**
S2534	24168-96-5	Isoconazole nitrate	N/A	Infection	**149.62**
S1573	105628-07-7	Fasudil (HA-1077) HCl	N/A	Cardiovascular Disease	**149.59**
S2787	248281-84-7	Laquinimod	ABR-215062, LAQ	Immunology	**149.42**
S1569	118292-40-3	Tazarotene	AGN190168	Inflammation	**149.09**
S4003	434-13-9	Lithocholic acid	N/A	Neurological Disease	**148.94**
S1278	645-05-6	Altretamine	Hexamethylmelamine, NSC13875, ENT50852	Cancer	**148.89**
S1465	186826-86-8	Moxifloxacin HCl	BAY12-8039 HCl	Infection	**148.85**
S2264	71963-77-4	Artemether	SM-224, CGP 56696	Cancer	**148.77**
S7645	934526-89-3	Pilaralisib (XL147)	N/A	Cancer	**148.03**
S2058	2398-96-1	Tolnaftate	N/A	Cancer	**147.95**
S4507	68-96-2	17-Hydroxyprogesterone	N/A	Endocrinology	**147.79**
S4848	171500-79-1	Dalbavancin	zeven	Infection	**147.30**
S3043	162011-90-7	Rofecoxib	MK-0966	Gastroenterology	**147.17**
S2020	43229-80-7	Formoterol Hemifumarate	Eformoterol, CGP 25827A, NSC 299587, YM 08316	Neurological Disease	**147.13**
S1030	404950-80-7	Panobinostat (LBH589)	NVP-LBH589	Epigenetics	**147.12**
S3023	2438-72-4	Bufexamac	N/A	Immunology/Inflammation	**146.97**
S1206	104344-23-2	Bisoprolol fumarate	EMD33512	Cardiovascular Disease	**146.67**
S5368	585-86-4	Lactitol	N/A	Others	**146.63**
S5028	504-24-5	4-Aminopyridine	fampridine, dalfampridine	Transmembrane Transporters	**146.47**
S1705	57-83-0	Progesterone	N/A	Endocrinology	**146.42**
S5005	66309-69-1	Cefotiam hydrochloride	Cefotiam dihydrochloride, Halospor, Pansporin, Pansporine	Infection	**146.24**
S1281	72432-10-1	Aniracetam	RO 13-5057	Neurological Disease	**146.18**

S2157	1258861-20-9	Taladegib (LY2940680)	N/A	Cancer	**146.16**
S2042	427-51-0	Cyproterone Acetate	N/A	Cancer/Endocrinology	**146.16**
S2126	57149-07-2	Naftopidil	KT-611	KT-611, BM-15275	**145.78**
S1404	13647-35-3	Trilostane	WIN 24540	Endocrinology	**145.58**
S1606	23593-75-1	Clotrimazole	BAY b 5097, FB 5097	Infection	**145.44**
S1576	599-79-1	Sulfasalazine	Azulfidine	Inflammation	**145.29**
S7852	491833-29-5	Eliglustat	GENZ-112638	Others	**145.24**
S4550	123-99-9	Azelaic acid	Nonanedioic acid, Finacea, Azelex, Anchoic acid	Infection	**145.09**
S1284	86541-74-4	Benazepril HCl	CGS 14824A HCl	Cardiovascular Disease	**144.99**
S1311	57248-88-1	Pamidronate Disodium	CGP 23339AE	Metabolic Disease	**144.88**
S1237	85622-93-1	Temozolomide	CCRG81045, NSC 362856	Cancer	**144.84**
S5623	843663-66-1	Bedaquiline	TMC207; R207910	Immunology	**144.83**
S2561	50-41-9	Clomifene citrate	NSC 35770	Endocrinology	**144.80**
S2874	59721-29-8	Camostat Mesilate	FOY-305	Inflammation	**144.77**
S4566	16485-10-2	DL-Panthenol	DL-Pantothenol, DL-Pantothenyl alcohol	Others	**144.57**
S7028	1201438-56-3	Duvelisib (IPI-145, INK1197)	N/A	Cancer	**144.39**
S2925	1186486-62-3	Evacetrapib (LY2484595)	N/A	Metabolic Disease	**144.24**
S1875	1197-18-8	Tranexamic Acid	N/A	Cardiovascular Disease	**144.21**
S3038	265121-04-8	Fosaprepitant dimeglumine salt	MK-0517	Cardiovascular Disease	**144.11**
S4680	24305-27-9	Protirelin	Thyroliberin, Lopremone, Synthetic TRH, Thyrotropin-releasing hormone, Rifathyroin	Endocrinology	**143.79**
S4986	64953-12-4	Latamoxef sodium	Moxalactam sodium salt	Microbiology	**143.75**
S4387	73-48-3	Bendroflumethiazide	Bendrofluazide	Others	**143.56**
S5508	853-23-6	Dehydroepiandrosterone acetate	DHEA acetate, androstenolone acetate	Others	**143.26**
S1505	78110-38-0	Aztreonam	SQ 26776	Infection	**143.12**
S3155	1722-62-9	Mepivacaine HCl	N/A	Neurological Disease	**143.04**
S1774	154-42-7	Thioguanine	N/A	Cancer	**142.99**
S2487	24280-93-1	Mycophenolic acid	Mycophenolate, RS-61443	Immunology	**142.98**
S5535	354812-41-2	Moxifloxacin		Infection	**142.91**
S7179	1230487-00-9	BAF312 (Siponimod)	N/A	Immunology	**142.89**
S3139	100-97-0	Methenamine	Mandelamine	Inflammation	**142.76**
S3621	163680-77-1	Pazufloxacin mesylate	T-3762, Pazucross	Infection	**142.70**

S1549	152520-56-4	Nebivolol HCl	R-65824	Cardiovascular Disease	**142.64**
S4597	132866-11-6	Lercanidipine hydrochloride	Zanidip (hydrochloride), Masnidipine (hydrochloride)	Cardiovascular Disease	**142.52**
S1825	84611-23-4	Erdosteine	KW-9144	Respiratory Disease	**142.51**
S3872	90-05-1	Guaiacol	o-methoxyphenol, 2-hydroxyanisole, O-methylcatechol	Others	**142.17**
S3746	82186-77-4	Lumefantrine	benflumetol	Infection	**142.11**
S4612	80-08-0	Dapson	Dapsone, 4,4′-Diaminodiphenyl sulfone, 4,4′-Sulfonyldianiline, 4-Aminophenyl sulfone, Bis(4-aminophenyl) sulfone, DDS	Infection	**142.00**
S2757	918633-87-1	Evofosfamide (TH-302)	Evofosfamide	Cancer	**141.91**
S5389	936539-80-9	Betrixaban maleate	N/A	Protease	**141.90**
S2504	36791-04-5	Ribavirin	NSC-163039, ICN-1229 ,RTCA, Tribavirin	Infection	**141.82**
S5097	7413-34-5	Methotrexate disodium	N/A	Metabolism	**141.73**
S4748	103639-04-9	Ondansetron Hydrochloride Dihydrate	N/A	Neurological Diseases	**141.63**
S3078	5534-09-8	Beclomethasone dipropionate	Beclometasone dipropionate	Inflammation	**141.63**
S1395	1405-20-5, 4135-11-9 (free base)	Polymyxin B sulphate	Aerosporin, PMB, Poly-RX	Infection	**141.46**
S5361	20187-55-7	Bendazac	bendazolic acid, AF-983	Immunology & Inflammation	**141.44**
S1831	72956-09-3	Carvedilol	BM-14190, SKF 105517	Cardiovascular Disease	**141.33**
S7818	1029044-16-3	Pexidartinib (PLX3397)	N/A	Cancer	**141.20**
S4815	52-89-1	L-Cysteine HCl	N/A	Others	**141.19**
S1840	13010-47-4	Lomustine	nsc79037	Cancer	**141.04**
S5245	871038-72-1	Raltegravir potassium	N/A	Microbiology	**140.97**
S1499	70356-03-5	Cefaclor	Cefachlor, Cefaclorum	Microbiology	**140.94**
S4180	23327-57-3	Nefopam HCl	N/A	Neurological Disease	**140.83**
S2131	162401-32-3	Roflumilast	APTA 2217, B9302-107, BY 217, BYK 20869	Neurological Disease	**140.80**
S1671	60-32-2	(6-) ε-?Aminocaproic acid	N/A	Cardiovascular Disease	**140.73**
S1898	105826-92-4	Tropisetron HCl	ICS 205-930	Neurological Disease	**140.72**

S4062	7681-76-7	Ronidazole	N/A	Neurological Disease	**140.59**
S2111	231277-92-2	Lapatinib	GW-572016, GSK572016	Neurological Disease	**140.52**
S1703	76584-70-8	Divalproex Sodium	N/A	Neurological Disease	**140.50**
S1204	73-31-4	Melatonin	N/A	Endocrinology	**140.39**
S8134	926037-48-1	Radotinib	IY-5511	Cancer	**140.14**
S1701	638-94-8	Desonide	N/A	Inflammation	**140.12**
S1481	167465-36-3	Zosuquidar (LY335979) 3HCl	RS 33295-198 (D06387) 3HCl	Cancer	**140.09**
S1221	4342-03-4	Dacarbazine	DTIC-Dome	Cancer	**140.02**
S3673	526-08-9	Sulfaphenazole	Depocid, Depotsulfonamide, Plisulfan, Raziosulfa	Infection	**139.93**
S4110	50-50-0	Estradiol Benzoate	N/A	Endocrinology	**139.64**
S2130	5908-99-6	Atropine sulfate monohydrate	N/A	Respiratory Disease	**139.61**
S2030	30484-77-6	Flunarizine 2HCl	KW-3149, R14950	Neurological Disease	**139.50**
S3047	26095-59-0	Otilonium Bromide	N/A	Gastroenterology	**139.43**
S5430	51384-51-1	Metoprolol	N/A	Neuronal Signaling	**139.05**
S3193	29457-07-6	Ticarcillin sodium	AB 2288, BRL 2288	Infection	**138.95**
S2479	859-18-7	Lincomycin HCl	NSC 70731	Cancer	**138.93**
S1611	62893-19-0	Cefoperazone	N/A	Infection	**138.70**
S4683	1308285-21-3	Sildenafil Mesylate	N/A	Cardiovascular Disease	**138.48**
S1914	145-13-1	Pregnenolone	N/A	Neurological Disease	**138.44**
S3996	1077-28-7	Thioctic acid	DL-α-Lipoic Acid	Others	**138.40**
S4089	66852-54-8	Halobetasol Propionate	BMY-30056, CGP-14458	Inflammation	**138.31**
S3722	241479-67-4	Isavuconazole	BAL-4815, RO-0094815	Infection	**138.08**
S1482	1009119-64-5	Daclatasvir (BMS-790052)	EBP883	Cancer	**137.91**
S1770	68-35-9	Sulfadiazine	N/A	Infection	**137.86**
S1778	70-00-8	Trifluridine	NSC 529182, NSC 75520	Infection	**137.85**
S1894	137862-53-4	Valsartan	CGP-48933	Cardiovascular Disease	**137.80**
S1949	58-27-5	Menadione	Vitamin K3	Endocrinology	**137.39**
S1260	364782-34-3	Cinacalcet HCl	AMG-073 HCl	Endocrinology	**137.19**
S2159	161715-24-8	Tebipenem Pivoxil	L-084, ME1211	Infection	**137.18**
S3647	13009-99-9	Mafenide Acetate	N/A	N-(2-amino-4-[fluorobenzylamino]-phenyl) carbamic acid (2HCl), D-23129 2HCl	**136.97**
S2374	56-69-9	5-hydroxytryptophan (5-HTP)	NSC-92523	Neurological Disease	**136.90**
S4085	116209-55-3	Levobetaxolol HCl	AL 1577A	Cardiovascular Disease	**136.89**
S3059	93106-60-6	Enrofloxacin	BAY-Vp2674, PD160788	Infection	**136.75**

S1759	147526-32-7	Pitavastatin Calcium	NK-104, P-872441, itavastatin, nisvastatin	Cardiovascular Disease	**136.62**
S4397	537-12-2	Diperodon HCl	N/A	Others	**136.59**
S1594	425386-60-3	Semagacestat (LY450139)	N/A	Neurological Disease	**136.53**
S8266	148-82-3	Melphalan	Alkeran, Sarcolysin, L-PAM	Cancer	**136.45**
S4549	28300-74-5	Antimonyl potassium tartrate trihydrate	Tartar emetic	Infection	**136.39**
S3129	738-70-5	Trimethoprim	BW 56-72, NIH 204, NSC-106568	Infection	**136.34**
S4293	58-33-3	Promethazine HCl	N/A	Inflammation/Neurological Disease	**136.25**
S1469	19356-17-3	Calcifediol	25-hydroxyvitamin D3	Metabolic Disease	**136.24**
S4044	69004-03-1	Toltrazuril	N/A	Infection	**136.13**
S1574	285983-48-4	Doramapimod (BIRB 796)	N/A	Immunology	**135.91**
S3163	132-17-2	Benztropine mesylate	N/A	Neurological Disease	**135.89**
S1750	6197-30-4	Octocrylene	N/A	Others	**135.71**
S4604	138199-71-0	Levofloxacin hydrate	Levofloxacin hemihydrate, Levaquin hydrate, Tavanic hydrate, Quixin hydrate, Iquix hydrate, Cravit hydrate	Infection	**135.50**
S4359	51-83-2	Carbachol	Carbamylcholine	Others	**135.29**
S5159	564-25-0	Doxycycline	Vibramycin, Doxytetracycline, Doxiciclina, Doxycyclinum	Microbiology	**135.19**
S1593	503612-47-3	Apixaban	BMS 562247-01	Cardiovascular Disease	**134.94**
S4887	59-26-7	Nikethamide	Corvin, Anacardone	Others	**134.74**
S5560	35457-80-8	Midecamycin	Espinomycin A, Medecamycin A1, Platenomycin B1, Rubimycin, Turimycin P3	Microbiology	**134.74**
S4312	6804-07-5	Carbadox	N/A	Others	**134.72**
S2037	145040-37-5	Candesartan Cilexetil	TCV-116	Cardiovascular Disease	**134.65**
S1847	14976-57-9	Clemastine Fumarate	N/A	Immunology	**134.60**
S3051	157212-55-0	Bosentan Hydrate	Ro 47-0203	Cardiovascular Disease/Respiratory Disease	**134.58**
S1470	252916-29-3	Orantinib (TSU-68, SU6668)	NSC 702827	Cancer	**134.47**
S4577	2451-01-6	Terpin hydrate	N/A	Respiratory Disease	**134.16**

S2797	193275-84-2	Lonafarnib	SCH66336	Infection	**134.15**
S3671	152-43-2	Quinestrol	Ethinylestradiol 3-cyclopentyl ether, EE2CPE, W-3566	Endocrinology	**134.15**
S2046	112529-15-4	Pioglitazone HCl	AD-4833, U-72107E	Metabolic Disease	**134.13**
S2011	191217-81-9	Pramipexole 2HCl	N/A	Neurological Disease	**133.95**
S4977	111011-63-3	Efonidipine	NZ-105	Transmembrane Transporters	**133.91**
S1287	28395-03-1	Bumetanide	N/A	Cardiovascular Disease	**133.82**
S2248	1009820-21-6	Silmitasertib (CX-4945)	N/A	Cancer	**133.67**
S2594	68497-62-1	Pramiracetam	CI-879	Endocrinology	**133.64**
S4283	6202-23-9	Cyclobenzaprine HCl	N/A	Neurological Disease	**133.54**
S4630	364-98-7	Diazoxide	Sch-6783, SRG-95213, Proglycem	Cardiovascular Disease	**133.49**
S2536	22916-47-8	Miconazole	N/A	Daktarin IV, MJR 1762, Monistat IV, NSC 170986, R 18134, Zimybase	**133.32**
S4195	521-74-4	Broxyquinoline	N/A	Infection	**133.06**
S4333	6968-72-5	Mepiroxol	N/A	Others	**133.03**
S5081	60925-61-3	Ceforanide	N/A	Microbiology	**133.00**
S1680	97-77-8	Disulfiram	NSC 190940	Neurological Disease	**132.92**
S4098	3093-35-4	Halcinonide	N/A	Immunology	**132.91**
S4304	117-37-3	Anisindione	N/A	Others	**132.83**
S3619	33818-15-4	Citicoline sodium	CDP-choline	Neurological Diseases	**132.79**
S2173	1137608-69-5	Telotristat Etiprate (LX 1606 Hippurate)	N/A	Gastroenterology	**132.79**
S2875	179474-81-8	Prucalopride	R-93877	Neuronal Signaling	**132.78**
S1488	143388-64-1	Naratriptan HCl	N/A	Neurological Disease	**132.50**
S5433	150-90-3	Sodium succinate	Sodium 3-carboxypropanoate, Disodium butanedioate	Others	**132.28**
S2229	496775-62-3	Eltrombopag Olamine	SB-497115-GR, SB497115	Cancer	**132.21**
S1371	122841-12-7	Cefoselis Sulfate	FK 037 Sulfate	Infection	**132.19**
S4538	138786-67-1	Pantoprazole sodium	SKF96022 sodium, BY-1023 sodium	SKF96022 sodium, BY-1023 sodium	**132.15**
S4835	89796-99-6	Aceclofenac	Preservex, Airtal	Immunology & Inflammation	**131.99**
S1715	29094-61-9	Glipizide	CP-28720	Endocrinology	**131.96**
S1638	15687-27-1	Ibuprofen	N/A	Inflammation	**131.86**
S3162	74610-55-2	Tylosin tartrate	N/A	Infection	**131.72**
S1196	107868-30-4	Exemestane	FCE24304, PNU155971	Endocrinology	**131.67**
S1225	33419-42-0	Etoposide	VP-16, VP-16213	Cancer	**131.66**

S2047	70374-39-9	Lornoxicam	Chlortenoxicam	Inflammation	**131.62**
S4816	737-31-5	Diatrizoate sodium	amidotrizoate sodium, Hypaque sodium, Triombrin	Others	**131.56**
S1224	61825-94-3	Oxaliplatin	L-OHP	Cancer	**131.51**
S4849	130018-87-0	Levocetirizine Dihydrochloride	Xyzal Dihydrochloride, (-)-Cetirizine Dihydrochloride	Neuronal Signaling	**131.48**
S2077	134523-03-8	Atorvastatin Calcium	N/A	Cardiovascular Disease	**131.22**
S1271	56180-94-0	Acarbose	BAY g 5421	Metabolic Disease	**131.19**
S2045	3094-09-5	Doxifluridine	5'-DFUR, AMC 0101	Immunology	**131.19**
S5527	89365-50-4	Salmeterol	Astmerole, GR-33343X, SN408D	Neuronal Signaling	**131.18**
S1749	56-85-9	L-Glutamine	N/A	Metabolic Disease	**131.16**
S3063	78213-16-8	Diclofenac Diethylamine	N/A	Neurological Disease	**131.03**
S4407	30718	Prochlorperazine Dimaleate	N/A	Others	**131.02**
S7930	219989-84-1	Ixabepilone (BMS-247550)	Azaepothilone B, BMS 247550, BMS 247550-1, Ixempra	Cytoskeletal Signaling	**130.92**
S1601	50-55-5	Reserpine	N/A	Cardiovascular Disease	**130.91**
S1631	17795-21-0	Allopurinol Sodium	N/A	Metabolic Disease	**130.87**
S2790	155270-99-8	Istradefylline	KW-6002	Neurological Diseases	**130.84**
S1869	129938-20-1	Dapoxetine HCl	LY-210448	Neurological Disease	**130.79**
S1028	388082-77-7	Lapatinib (GW-572016) Ditosylate	N/A	Cancer	**130.67**
S3060	86347-15-1	Medetomidine HCl	N/A	Inflammation	**130.66**
S2003	376348-65-1	Maraviroc	UK-427857	Inflammation	**130.63**
S4666	201677-61-4	Sivelestat sodium tetrahydrate	Sivelestat sodium, ONO5046-Na, Sodium sivelestat, EI546 sodium, LY544349 sodium	Respiratory Disease	**130.63**
S3212	71320-77-9	Moclobemide (Ro 111163)	N/A	Neurological Disease	**130.47**
S7579	1256388-51-8	Ledipasvir (GS5885)	N/A	Infection	**130.40**
S2549	10405-02-4	Trospium chloride	N/A	Cardiovascular Disease	**130.38**
S2251	102518-79-6	(-)-Huperzine A (HupA)	N/A	Neuronal Signaling	**130.21**
S2179	1229236-86-5	Gandotinib (LY2784544)	N/A	Cancer	**130.13**

S1222	149003-01-0	Dexrazoxane HCl (ICRF-187, ADR-529)	ICRF-187 (ADR-529) HCl	Cardiovascular Disease	**130.06**
S5398	82752-99-6	Nefazodone hydrochloride		Others	**129.83**
S4222	59703-84-3	Piperacillin Sodium	CL227193	Infection	**129.64**
S5352	75498-96-3	Cefminox Sodium	Meicelin, MT-141	Microbiology	**129.47**
S1813	111470-99-6	Amlodipine Besylate	N/A	Istin	**129.39**
S4532	60166-93-0	Iopamidol	Iopamiro, Isovue, Iopamiron, Niopam, Solutrast	Diagnosis	**129.30**
S2286	59865-13-3	Cyclosporin A	Cyclosporine A	Immunology	**129.23**
S1405	50700-72-6	Vecuronium Bromide	ORG NC45	Neurological Disease	**129.17**
S1440	375815-87-5	Varenicline Tartrate	CP 526555-18	Neurological Diseases	**129.08**
S1848	458-37-7	Curcumin	Diferuloylmethane	Cancer	**128.94**
S1397	119302-91-9	Rocuronium Bromide	ORG 9426	Neurological Disease	**128.94**
S3663	56287-74-2	Afloqualone	HQ-495	Neurological Diseases	**128.86**
S4094	5086-74-8	Tetramisole HCl	N/A	Infection	**128.78**
S1691	55268-74-1	Praziquantel	N/A	Infection	**128.76**
S4295	6385-02-0	Meclofenamate Sodium	N/A	Inflammation	**128.76**
S5353	74849-93-7	Cefpiramide sodium	SM-1652, wy-44635	Microbiology	**128.70**
S3718	74381-53-6	Leuprolide Acetate	Leuprorelin Acetate	Cancer	**128.69**
S5638	113981-44-5	Cefozopran hydrochloride		Others	**128.68**
S3032	130641-38-2	Bindarit	AF 2838	Cardiovascular Disease	**128.66**
S5648	123663-49-0	Iguratimod	T-614	Immunology & Inflammation	**128.51**
S5412	80382-23-6	Loxoprofen Sodium	N/A	Immunology	**128.51**
S1709	50-28-2	Estradiol	N/A	Endocrinology	**128.35**
S4649	104075-48-1	Atipamezole hydrochloride	Antisedan, MPV-1248 hydrochloride, MPV1248 hydrochloride, MPV 1248 hydrochloride	Neurological Disease	**128.30**
S8135	625115-55-1	Riociguat (BAY 63-2521)	N/A	Cardiovascular Disease/Respiratory Disease	**128.24**
S2076	83915-83-7	Lisinopril	N/A	Cardiovascular Disease	**128.24**
S1619	721-50-6	Prilocaine	NSC 40027	Neurological Disease	**128.11**
S2614	300-08-3	Arecoline HBr	N/A	Neurological Disease	**127.86**
S3117	1508-65-2	Oxybutynin chloride	N/A	Neurological Disease	**127.76**
S2061	75330-75-5	Lovastatin	MK-803	Respiratory Disease	**127.63**
S1689	1247-42-3	Meprednisone	NSC 527579, SCH 4358	Inflammation	**127.56**

S5281	72822-13-0	Dapiprazole Hydrochloride	Glamidolo Hydrochloride, Reversil Hydrochloride	Neuronal Signaling	**127.48**
S4339	1084-65-7	Meticrane	N/A	Others	**127.44**
S1799	95635-55-5	Ranolazine	CVT 303, RS 43285-003	Ranexa	**127.41**
S1651	3424-98-4	Telbivudine	N/A	Infection	**127.40**
S5001	540737-29-9	Tofacitinib (CP-690550) Citrate	N/A	Cancer	**127.34**
S2553	5451-09-2	5-Aminolevulinic acid HCl	N/A	Neurological Disease	**127.15**
S1340	112811-59-3	Gatifloxacin	AM-1155, CG5501, BMS-206584	Infection	**127.12**
S3669	154-93-8	Carmustine	bis-chloroethylnitrosourea, BCNU, BiCNU|	Cancer	**127.02**
S4065	23256-50-0	Guanabenz Acetate	WY-8678 Acetate	Endocrinology	**127.00**
S4636	906093-29-6	Teneligliptin hydrobromide	Teneligliptin hydrobromide anhydrous	Metabolic Disease	**126.99**
S4517	64485-93-4	Cefotaxime sodium	N/A	Infection	**126.98**
S3151	33342-05-1	Gliquidone	Glurenorm	Metabolic Disease	**126.95**
S4376	6109-70-2	Aceclidine HCl	N/A	Others	**126.87**
S1939	16595-80-5	Levamisole hydrochloride	N/A	Immunology/Infection	**126.83**
S1643	128-13-2	Ursodiol	N/A	Metabolic Disease	**126.81**
S1657	84680-54-6	Enalaprilat Dihydrate	MK-422 Dihydrate	Cardiovascular Disease	**126.81**
S2814	1217486-61-7	Alpelisib (BYL719)	N/A	Cancer	**126.78**
S4093	880149-29-1	Bismuth Subcitrate Potassium	N/A	Gastroenterology	**126.75**
S8012	686347-12-6	Otenabant (CP-945598) HCl	Otenabant	Metabolic Disease	**126.72**
S4651	202409-33-4	Etoricoxib	Arcoxia, MK-663, MK-0663, Tauxib, Algix, Nucoxia	Inflammation	**126.71**
S3181	42835-25-6	Flumequine	N/A	Infection	**126.64**
S2344	94-62-2	Piperine	1-Piperoylpiperidine	Cancer	**126.59**
S1921	83-12-5	Phenindione	Rectadione, phenylindandione	Cardiovascular Disease	**126.55**
S3199	98769-84-7	Reboxetine mesylate	PNU 155950E	Neurological Disease	**126.49**
S1339	1953-04-4	Galanthamine HBr	N/A	Neurological Disease	**126.34**
S2035	82159-09-9	Epalrestat	ONO-2235	Metabolic Disease	**126.03**
S4585	116-43-8	Succinylsulfathiazole	Succinylsulphathiazole	Infection	**126.00**
S1293	132203-70-4	Cilnidipine	FRC-8653	Cardiovascular Disease	**125.92**
S3723	132907-72-3	Ramosetron Hydrochloride	YM-060	Gastroenterology	**125.91**
S2064	127294-70-6	Balofloxacin	N/A	Infection	**125.80**

S5296	75975-70-1	Cephradine monohydrate	N/A	Microbiology	**125.64**
S4618	67227-57-0	Fenoldopam mesylate	SKF 82526 (mesylate), Fenoldopam methanesulfonate, Corlopam mesylate	Cardiovascular Disease	**125.55**
S4682	68767-14-6	Loxoprofen	Koloxo, Loxoprofene, Loxoprofeno	Inflammation	**125.54**
S2052	6153-64-6	Oxytetracycline Dihydrate	N/A	Infection	**125.52**
S4731	58-39-9	Perphenazine	Perphenazine, Perphenazin, Trilafon, Etaperazine	Neurological Diseases	**125.50**
S4975	247257-48-3	Fimasartan	Kanarb	Endocrinology & Hormones	**125.38**
S4019	330784-47-9	Avanafil	N/A	Cardiovascular Disease	**125.34**
S4662	198904-31-3	Atazanavir	Latazanavir, Zrivada, Reyataz, BMS-232632	Infection	**125.34**
S3170	69-52-3	Ampicillin sodium	N/A	Infection	**125.33**
S2613	60200-06-8	Clorsulon	MK-401	Cancer	**125.33**
S7128	1403254-99-8	Tazemetostat (EPZ-6438)	E7438	Cancer	**125.32**
S4370	65-31-6	Nicotine Ditartrate	http://www.selleckchem.com/products/nicotiN/A	Others	**125.29**
S2001	697761-98-1	Elvitegravir (GS-9137, JTK-303)	N/A	Immunology	**125.24**
S4280	3685-84-5	Meclofenoxate (Centrophenoxine) HCl	N/A	Neurological Diseases	**125.19**
S4061	27262-48-2	Levobupivacaine HCl	(S)-(-)-Bupivacaine HCl	Neurological Disease	**125.06**
S3173	60-80-0	Antipyrine	Phenazone	Infection	**125.01**
S1696	50-23-7	Hydrocortisone	N/A	Inflammation	**125.00**
S4057	135-07-9	Methyclothiazide	N/A	Cardiovascular Disease	**124.97**
S5709	6533-00-2	Norgestrel	WY-3707, SH-70850, SH-850, FH 122-A	Others	**124.91**
S2152	163253-35-8	Sitafloxacin Hydrate	DU-6859a	Cancer	**124.87**
S4152	2624-44-4	Ethamsylate	Etamsylate	Cardiovascular Disease	**124.81**
S8021	960203-27-4	Vortioxetine (Lu AA21004) HBr	N/A	Neurological Diseases	**124.78**
S4542	52-49-3	Trihexyphenidyl hydrochloride	Benzhexol hydrochloride, Artane hydrochloride	Neurological Disease	**124.73**
S5098	184475-55-6	Gefitinib hydrochloride	ZD1839 hydrochloride	Protein Tyrosine Kinase	**124.60**
S4503	137-08-6	Calcium D-Panthotenate	D-Pantothenic Acid Calcium	Others	**124.59**
S4279	64-73-3	Demeclocycline HCl	N/A	Infection	**124.57**
S3149	979-32-8	Estradiol valerate	N/A	Endocrinology	**124.57**

S1613	160970-54-7	Silodosin	KAD 3213, KMD 3213	Cardiovascular Disease	**124.45**
S4040	68-22-4	Norethindrone	Norethisterone	Neurological Disease	**124.31**
S2388	541-15-1	L-carnitine	Levocarnitine	Metabolic Disease	**124.25**
S1816	113-92-8	Chlorpheniramine Maleate	NCI-C55265	Neurological Disease	**124.24**
S2476	84625-61-6	Itraconazole	R 51211	Cancer	**124.18**
S5659	87233-61-2	Emedastine	N/A	Neuronal Signaling	**124.03**
S5006	6809-52-5	Teprenone	geranylgeranylacetone, Tetraprenylacetone	Others	**123.99**
S3179	4800-94-6	Carbenicillin disodium	BRL-2064	Infection	**123.85**
S1604	144689-63-4	Olmesartan Medoxomil	CS-866	Cardiovascular Disease	**123.82**
S1442	137234-62-9	Voriconazole	UK-109496	Infection	**123.56**
S3201	440-17-5	Trifluoperazine 2HCl	SKF5019	Neurological Disease	**123.52**
S4535	61-73-4	Methylene Blue	Basic Blue 9, Tetramethylthionine chloride, methylthioninium chloride, CI-52015	Infection	**123.50**
S1635	114-07-8	Erythromycin	E-Mycin	E-Mycin	**123.09**
S7634	1369761-01-2	Cerdulatinib (PRT062070, PRT2070)	N/A	Cancer	**122.69**
S5069	1195768-06-9	Dabrafenib Mesylate	GSK2118436 Mesylate	MAPK	**122.62**
S2118	122647-32-9	Ibutilide Fumarate	U-70226E	Cardiovascular Disease	**122.60**
S4178	38083-17-9	Climbazole	N/A	Infection	**122.60**
S5209	84294-96-2	Enoxacin Sesquihydrate	Enoxacin hydrate	Infection	**122.55**
S4388	37106-97-1	Bentiromide	N/A	Others	**122.54**
S1251	65928-58-7	Dienogest	STS 557	Endocrinology	**122.52**
S8206	1448347-49-6	Ivosidenib (AG-120)		Metabolism	**122.50**
S4208	4940-39-0	Chromocarb	N/A	Cardiovascular Disease	**122.45**
S2452	31677-93-7	Amfebutamone (Bupropion) HCl	N/A	Infection	**122.34**
S3211	67-03-8	Thiamine HCl (Vitamin B1)	N/A	Metabolic Disease	**122.31**
S3732	1192491-61-4	Avibactam sodium	AVE-1330A, NXL104	Infection	**122.24**
S4927	294-90-6	Cyclen	1,4,7,10-tetraazacyclododecane	Others	**122.12**
S1899	98-92-0	Nicotinamide (Vitamin B3)	Niacinamide, Vitamin PP, Nicotinic acid amide	Metabolic Disease	**122.10**
S7856	379270-37-8	Tenofovir Alafenamide (GS-7340)	N/A	Infection	**122.06**
S2484	78415-72-2	Milrinone	Win 47203	Cardiovascular Disease	**121.97**

S1775	52-24-4	Thiotepa	Tiofosyl, Tiofosfamid, Triethylenethiophosphoramide	Cancer	**121.86**
S1603	54-31-9	Furosemide	N/A	Cardiovascular Disease	**121.85**
S4177	66-22-8	Uracil	N/A	Cancer	**121.83**
S2603	4991-65-5	Tioxolone	N/A	Endocrinology	**121.76**
S2265	88495-63-0	Artesunate	WR-256283	Infection	**121.76**
S4326	452-35-7	Ethoxzolamide	N/A	Others	**121.75**
S4308	91-33-8	Benzthiazide	Benzothiazide	Others	**121.73**
S1777	536-33-4	Ethionamide	Bayer 5312	Infection	**121.70**
S4527	42017-89-0	Fenofibric acid	NSC 281318, Trilipix, FNF acid	Cardiovascular Disease	**121.63**
S2507	51022-70-9	Salbutamol Sulfate	Albuterol	Respiratory Disease	**121.63**
S3635	520-85-4	Medroxyprogesterone	MP	Endocrinology	**121.56**
S1502	15686-71-2	Cephalexin	Cefalexin	Infection	**121.48**
S3113	58-56-0	Pyridoxine HCl	Vitamin B6	Endocrinology	**121.46**
S1212	3543-75-7	Bendamustine HCl	SDX-105 (Cytostasane) HCl	Cancer	**121.39**
S5484	1501-84-4	Rimantadine Hydrochloride	N/A	DNA Damage	**121.38**
S1748	63675-72-9	Nisoldipine	BAY K 5552	Cardiovascular Disease	**121.37**
S1808	21829-25-4	Nifedipine	N/A	Cardiovascular Disease	**121.37**
S8517	320367-13-3	Lixisenatide	Lyxumia, Adlyxin, ZP10A peptide, AVE0010	GPCR & G Protein	**121.20**
S4932	603-00-9	Proxyphylline	Monophylline, Spasmolysin	GPCR & G Protein	**121.15**
S4377	113-52-0	Imipramine HCl	Melipramine HCl	Others	**121.15**
S1965	125-33-7	Primidone	NCI-C56360	Neurological Disease	**121.13**
S2557	78628-80-5	Terbinafine HCl	KWD 2019	Infection	**121.11**
S1767	7235-40-7	Beta Carotene	N/A	Metabolism	**121.10**
S5023	42200-33-9	Nadolol	Corgard, Solgol, Anabet	Endocrinology & Hormones	**121.08**
S1683	22254-24-6	Ipratropium Bromide	N/A	Respiratory Disease	**120.85**
S3073	179463-17-3	Caspofungin Acetate	MK-0991	Infection	**120.84**
S2517	10347-81-6	Maprotiline HCl	N/A	Neurological Disease	**120.83**
S3130	58-85-5	Biotin (Vitamin B7)	N/A	Others	**120.83**
S4980	249921-19-5	Anamorelin	ONO-7643, RC-1291, ST-1291	Others	**120.79**
S1666	2022-85-7	Flucytosine	N/A	Infection	**120.65**
S3138	56-04-2	Methylthiouracil	NSC-193526, NSC-9378	Infection	**120.61**
S1662	75695-93-1	Isradipine	PN 200-110	Neurological Disease	**120.61**
S2216	366017-09-6	Mubritinib (TAK 165)	N/A	Cancer	**120.59**
S4561	117-10-2	Danthron	chrysazin, Antrapurol	Gastroenterology	**120.55**

S2038	65-28-1	Phentolamine Mesylate	N/A	Cardiovascular Disease	**120.50**
S5074	141396-28-3	Argatroban Monohydrate	Argatroban hydrate, Argipidine	Others	**120.50**
S4393	24356-60-3	Cephapirin Sodium	Cefapirin Sodium	Others	**120.42**
S4000	66104-23-2	Pergolide Mesylate	LY127809	Neurological Disease	**120.41**
S5051	51940-44-4	Pipemidic acid	Acido pipemidico	Microbiology	**120.39**
S3052	182349-12-8	Rupatadine Fumarate	N/A	Inflammation	**120.11**
S1725	91161-71-6	Terbinafine	SF 86-327	Lamisil, Terbinex, Corbinal, Zabel	**120.09**
S5004	137071-32-0	Pimecrolimus	ASM 981	Cancer	**120.02**
S4663	751-94-0	Fusidate Sodium	Fucidin, Sodium fusidate, SQ-16360	Infection	**119.96**
S4430	747-36-4	Hydroxychloroquine Sulfate	N/A	Infection	**119.79**
S5365	850649-61-5	Alogliptin	SYR 322	Proteases	**119.78**
S4689	83-44-3	Deoxycholic acid	Deoxycholate, Desoxycholic acid, Cholerebic, Cholorebic	Others	**119.72**
S1250	915087-33-1	Enzalutamide (MDV3100)	N/A	Cancer	**119.61**
S1716	10238-21-8	Glyburide (Glibenclamide)	N/A	Endocrinology	**119.60**
S5533	79645-27-5	Tobramycin sulfate		Others	**119.57**
S2106	123040-16-4	Azasetron HCl	Y-25130 HCl	Neuronal Signaling	**119.57**
S3106	121808-62-6	Pidotimod	N/A	Immunology	**119.55**
S4253	108929-04-0	Epinastine HCl	WAL-801CL HCl	Inflammation/Neurological Disease	**119.54**
S2059	70024-40-7	Terazosin HCl Dihydrate	N/A	Neuronal Signaling	**119.49**
S4516	464-49-3	(+)-Camphor	N/A	Infection	**119.42**
S2108	42461-84-7	Meglumin	N/A	Immunology	**119.41**
S5356	80370-57-6	Ceftiofur	N/A	Microbiology	**119.39**
S5371	4360-12-7	Ajmaline	Ajmalan-17	Cardiovascular Disease	**119.35**
S4650	104054-27-5	Atipamezole	MPV-1248, MPV1248, MPV 1248, Antisedan	MPV-1248, MPV1248, MPV 1248, Antisedan	**119.32**
S2489	105816-04-4	Nateglinide	A-4166	Immunology	**119.21**
S1357	137-58-6	Lidocaine	Alphacaine	Alphacaine, Xylocaine, lignocaine	**119.19**
S1353	65277-42-1	Ketoconazole	N/A	Infection	**119.15**
S4153	673-06-3	D-Phenylalanine	N/A	Others	**119.10**

S4952	110-17-8	Fumaric acid	2-Butenedioic acid, Trans-Butenedioic acid, Allomaleic acid, Boletic acid, Donitic acid, Lichenic acid, Fumarate	Others	**118.94**
S5288	7280-37-7	Estropipate	Piperazine estrone sulfate; 3-Sulfatoxyestra-1,3,5(10)-trien-17-one piperazine Salt;	Endocrinology	**118.90**
S4734	150812-13-8	Retigabine 2HCl	N-(2-amino-4-[fluorobenzylamino]-phenyl) carbamic acid (2HCl), D-23129 2HCl	MK-8931	**118.88**
S1304	595-33-5	Megestrol Acetate	BDH1298, SC10363	Cancer/Endocrinology	**118.87**
S4143	23142-01-0	Pentoxyverine Citrate	Carbetapentane Citrate	Neuronal Signaling	**118.87**
S1282	63968-64-9	Artemisinin	Qinghaosu,Artemisinine	Immunology/Infection	**118.82**
S3659	514-36-3	Fludrocortisone acetate	9α-fluorocortisol acetate	Imflammation	**118.80**
S3031	668270-12-0	Linagliptin	BI-1356	Cancer	**118.59**
S4600	100-51-6	Benzyl alcohol	Phenylmethanol, Benzenemethanol, Phenylcarbinol, Benzoyl alcohol	Others	**118.54**
S3121	16773-42-5	Ornidazole	Ro7-0207, NSC 95075	Gastroenterology	**118.49**
S2482	89226-75-5	Manidipine 2HCl	CV-4093	Transmembrane Transporters	**118.44**
S4647	75738-58-8	Cefmenoxime hydrochloride	Cefmenoxime hemihydrochloride, SCE-1365 hemihydrochloride	Infection	**118.38**
S1396	501-36-0	Resveratrol	N/A	Inflammation	**118.33**
S1507	138402-11-6	Irbesartan	BMS-186295, SR-47436	Cardiovascular Disease	**118.20**
S1675	136790-76-6	Lubiprostone	RU 0211	Transmembrane Transporters	**118.17**
S4588	577-11-7	Docusate Sodium	Aerosol OT, Bis(2-ethylhexyl) sulfosuccinate sodium salt, Constonate	Gastroenterology	**118.14**
S1452	336113-53-2	Ispinesib (SB-715992)	CK0238273	Cancer	**118.06**
S2044	969-33-5	Cyproheptadine HCl	N/A	Neuronal Signaling	**118.04**
S5537	51322-75-9	Tizanidine	N/A	Neuronal Signaling	**117.90**

S2084	136434-34-9	Duloxetine HCl	LY-248686 HCl	Neurological Disease	**117.88**
S5485	58-28-6	Desipramine Hydrochloride	Desmethylimipramine, Norimipramine, EX-4355, G-35020, JB-8181, NSC-114901	Neuronal Signaling	**117.73**
S2664	30299-08-2	Clinofibrate	N/A	Metabolic Disease	**117.68**
S1157	847499-27-8	Delanzomib (CEP-18770)	N/A	Cancer	**117.63**
S2125	72-33-3	Mestranol	N/A	Endocrinology	**117.59**
S1456	186497-07-4	Zibotentan (ZD4054)	N/A	Cancer	**117.55**
P1034	14636-12-5	Terlipressin Acetate	Gly-Gly-Gly-Cys-Tyr-Phe-Gln-Asn-Cys-Pro-Lys-Gly-NH2(Cys4-Cys9)	Angiogenesis	**117.53**
S2296	471-53-4	Enoxolone	Glycyrrhetin	Gastroenterology	**117.51**
S1210	59-05-2	Methotrexate	NCI-C04671	Cancer	**117.49**
S4008	100299-08-9	Pemirolast potassium	BMY26517	Immunology/Inflammation	**117.48**
S4270	62613-82-5	Oxiracetam	ISF 2522	Neurological Disease	**117.45**
S3077	89786-04-9	Tazobactam	Tazobactam acid, Tazobactamum	Infection	**117.37**
S2099	110221-44-8	Temocapril HCl	CS-622 HCl	Cardiovascular Disease	**117.29**
S1827	63659-19-8	Betaxolol HCl	SL 75212 HCl	Lokren ,Kerlone	**117.11**
S2023	2078-54-8	Propofol	N/A	Transmembrane Transporters	**117.10**
S1623	616-91-1	Acetylcysteine	N/A	Respiratory Disease	**117.10**
S1669	82034-46-6	Loteprednol etabonate	N/A	Inflammation	**117.09**
S7605	1206161-97-8	Filgotinib (GLPG0634)	N/A	Immunology	**117.02**
S5257	309-29-5	Doxapram	N/A	Others	**117.01**
S3640	5588-10-3	Methoxyphenamine Hydrochloride	2-methoxy-N-methylamphetamine Hydrochloride, OMMA Hydrochloride	Respiratory Disease	**116.96**
S1195	426219-18-3	TAK-700 (Orteronel)	N/A	Cancer	**116.90**
S5573	137330-13-3	Tilmicosin phosphate	N/A	Microbiology	**116.89**
S7358	1092364-38-9	Poziotinib (HM781-36B)	NOV120101	Cancer	**116.89**
S1969	77191-36-7	Nefiracetam	DZL 221	Neurological Disease	**116.88**
S1742	129618-40-2	Nevirapine	NSC 641530	Infection	**116.85**
S3706	135159-51-2	Sarpogrelate hydrochloride	MCI-9042	Neurological Diseases	**116.85**
S5003	104987-11-3	Tacrolimus (FK506)	FR900506	Cancer	**116.83**

S5644	104376-79-6	Ceftriaxone Sodium		Others	**116.80**
S1630	315-30-0	Allopurinol	N/A	Aloprim	**116.80**
S1866	147-24-0	Diphenhydramine HCl	N/A	Immunology	**116.77**
S1305	50-44-2	Mercaptopurine (6-MP)	N/A	Cancer	**116.72**
S4931	54556-98-8	Propiverine hydrochloride	N/A	Others	**116.71**
S2575	1404-93-9	Vancomycin HCl	N/A	Infection	**116.70**
S4528	67-45-8	Furazolidone	N/A	Infection	**116.50**
S5238	242478-37-1	Solifenacin		Neurological Disease	**116.48**
S2607	35543-24-9	Buflomedil HCl	N/A	Cardiovascular Disease	**116.45**
S2494	140462-76-6	Olopatadine HCl	N/A	Neurological Disease	**116.39**
S3743	27025-41-8	Oxiglutatione	Oxidized glutathione, Glutathione disulfide, GSSG, Bi(glutathion-S-yl)	Others	**116.35**
S8219	845614-11-1	Bitopertin	RG1678, RO-4917838	Neurological Disease	**116.33**
S5206	113-98-4	Benzylpenicillin potassium	Penicillin G potassium	Infection	**116.30**
S2271	633-65-8	Berberine chloride	N/A	Microbiology	**116.28**
S4071	126-07-8	Griseofulvin	N/A	Infection	**116.28**
S4248	91714-93-1	Bromfenac Sodium	AHR 10282R	Inflammation	**116.17**
S5298	61336-70-7	Amoxicillin trihydrate	Amoxil trihydrate, Amoxipen trihydrate, Moxaline trihydrate	Microbiology	**116.16**
S2922	610798-31-7	Icotinib	BPI-2009H	Cancer	**116.16**
S5087	72797-41-2	Tianeptine	S-1574, JNJ-39823277, TPI-1062	Neuronal Signaling	**116.13**
S1047	149647-78-9	Vorinostat (SAHA, MK0683)	SAHA, MK0683, suberoylanilide hydroxamic acid	Cancer	**116.11**
S5532	3521-62-8	Erythromycin estolate		Others	**116.04**
S1053	209783-80-2	Entinostat (MS-275)	SNDX-275	Cancer	**115.93**
S4897	14383-51-8	Nortropine Hydrochloride	Nortropeno Hydrochloride	Others	**115.84**
S1257	171228-49-2	Posaconazole	SCH56592	Infection	**115.84**
S1387	57149-08-3	Naftopidil DiHCl	KT-611	Urology	**115.83**
S3731	183204-72-0	Tipiracil hydrochloride	MA-1 hydrochloride	Cancer	**115.69**
S4223	73573-88-3	Mevastatin	ML-236B	Cardiovascular Disease	**115.62**

				Di-Hydan, Dihycon, Dilabid, Diphedan, Diphenat, Diphenylan, Diphenylhydantoin, Hydantol, Lehydan, NS	
S2525	57-41-0	Phenytoin	Diphenylhydantoin		**115.62**
S5013	87771-40-2	Ioversol	N/A	Others	**115.49**
S4362	65513-72-6	Glafenine HCl	N/A	Others	**115.45**
S4536	67-20-9	Nitrofurantoin	Macrodantin, Furadantine, Furadonine	Infection	**115.43**
S5341	98418-47-4	Metoprolol succinate	Metoprolol succinate	Neuronal Signaling	**115.40**
S1331	86386-73-4	Fluconazole	UK 49858	Infection	**115.30**
S4843	127-08-2	Potassium acetate	Diuretic salt, Potassium ethanoate	Infection	**115.23**
S1578	139481-59-7	Candesartan	CV-11974	Cardiovascular Disease	**115.21**
S2538	103-84-4	Acetanilide	Antifebrin	Inflammation	**115.20**
S4826	21645-51-2	Aluminium hydroxide	Aluminic hydroxide, Aluminum trihydroxide, Aluminium(III) hydroxide	Others	**115.18**
S4391	54-30-8	Camylofin Chlorhydrate	Camylofine Chlorhydrate	Others	**115.13**
S5324	636-00-0	Oxidopamine (hydrobromide)	6-hydroxydopamine; 6-OHDA; 2,4,5-trihydroxyphenethylamine	Neuronal Signaling	**115.07**
S4839	112885-41-3	Mosapride	N/A	Neuronal Signaling	**114.95**
S1793	87333-19-5	Ramipril	N/A	Cardiovascular Disease	**114.87**
S3189	91374-20-8	Ropinirole HCl	SKF-101468A	Neurological Disease	**114.85**
S1197	98319-26-7	Finasteride	MK-906	Endocrinology	**114.81**
S1375	130693-82-2	Dorzolamide HCl	MK-507 (L-671152) HCl	Others	**114.75**
S2443	64-77-7	Tolbutamide	HLS 831	Cancer	**114.68**
S1636	1397-89-3	Amphotericin B	NSC 527017	Infection	**114.67**
S5282	81025-04-9	Lactitol monohydrate	N/A	Others	**114.54**
S1294	73963-72-1	Cilostazol	OPC-13013	Cardiovascular Disease	**114.53**
S5227	7704-67-8	Erythromycin thiocyanate	N/A	Microbiology	**114.51**
S4899	1321-14-8	Sulfogaiacol	guaiacolsulfonate	Others	**114.42**
S1855	70458-95-6	Pefloxacin Mesylate	N/A	Infection	**114.39**
S3002	366789-02-8	Rivaroxaban	BAY 59-7939	Metabolic Disease	**114.32**
S3036	81131-70-6	Pravastatin sodium	CS-514 Sodium	Metabolic Disease	**114.27**
S3083	753498-25-8	Indacaterol Maleate	QAB149	Respiratory Disease	**114.25**
S2221	1218779-75-9	Apatinib?mesylate	YN968D1	Cancer	**114.25**

S4146	1405-87-4	Bacitracin	N/A	Infection	**114.24**
S5717	968-81-0	Acetohexamide	N/A	Others	**114.23**
S1345	107007-99-8	Granisetron HCl	N/A	Neurological Disease	**114.20**
S2527	3963-95-9	Methacycline HCl	Rondomycin	Cancer	**114.16**
S1917	548-62-9	Crystal Violet	N/A	Infection	**113.99**
S4127	23031-32-5	Terbutaline Sulfate	N/A	Respiratory Disease	**113.89**
S2206	1025687-58-4	R788 (Fostamatinib) Disodium	Tamatinib Fosdium	Immunology	**113.60**
S2658	1086062-66-9	Omipalisib (GSK2126458, GSK458)	N/A	Cancer/Respiratory Disease	**113.55**
S1005	319460-85-0	Axitinib	AG 013736	Cancer	**113.54**
S1029	191732-72-6	Lenalidomide (CC-5013)	N/A	Cardiovascular Disease	**113.50**
S3019	41621-49-2	Ciclopirox ethanolamine	N/A	Infection	**113.48**
S4402	52432-72-1	Oxeladin Citrate	N/A	Others	**113.47**
S2602	53164-05-9	Acemetacin	K-708	Inflammation	**113.44**
S2566	51-30-9	Isoprenaline HCl	NCI-c55630	Neurological Disease	**113.41**
S1326	89-25-8	Edaravone	MCI-186	Cardiovascular Disease	**113.40**
S1333	56296-78-7	Fluoxetine HCl	Lilly110140	Neurological Disease	**113.39**
S2315	25389-94-0	Kanamycin sulfate	Kanamycin monosulfate, Ophtalmokalixan	Infection	**113.34**
S7513	865759-25-7	Trelagliptin	SYR-472	Metabolic Disease	**113.31**
S2830	18323-44-9	Clindamycin	N/A	Others	**113.27**
S4258	187164-19-8	Luliconazole	N/A	Infection	**113.25**
S2500	34183-22-7	Propafenone HCl	N/A	Cardiovascular Disease	**113.23**
S4291	32780-64-6	Labetalol HCl	N/A	Cardiovascular Disease	**113.08**
S1754	5633-20-5	Oxybutynin	N/A	Ditropan, Lyrinel XL, Oxytrol	**113.00**
S4750	6209-17-2	Sulfacetamide sodium salt hydrate	N/A	Infection	**113.00**
S4605	59-30-3	Folic acid	Folacin, Vitamin B9, Vitamin M, Pteroylglutamic acid, Folate	Others	**112.91**
S1776	89778-27-8	Toremifene Citrate	NSC 613680	Endocrinology	**112.90**
S1676	78613-38-4	Amorolfine HCl	N/A	Infection	**112.90**
S1424	19237-84-4	Prazosin HCl	cp-12299-1	Cardiovascular Disease	**112.88**
S3717	315-37-7	Testosterone Enanthate	Testosterone heptanoate, NSC-17591	Endocrinology	**112.78**
S4828	497-19-8	Sodium carbonate	Disodium carbonate, Soda Ash, Carbonic acid disodium salt	Others	**112.74**
S3015	26787-78-0	Amoxicillin	Amoxycillin	Infection	**112.71**
S1517	7681-93-8	Natamycin	Pimaricin	Infection	**112.70**

S4623	3166-62-9	Methylbenactyzine Bromide	Gastrimade, Noinarin, Paragone, Semulgin	Neuronal Signaling	**112.66**
S3716	167933-07-5	Flibanserin	BIMT-17, BIMT-17-BS	Endocrinology	**112.65**
S8294	1353550-13-6	Olmutinib (HM61713, BI 1482694)	BI 1482694, HM61713, HM71224	Cancer	**112.51**
S3997	33996-33-7	Oxaceprol	N-acetyl-L-hydroxyproline	Immunology & Inflammation	**112.48**
S4831	51-03-6	Piperonyl butoxide	Butacide, Ethanol butoxide, Pyrenone 606	Others	**112.43**
S4733	150812-12-7	Retigabine	N-(2-amino-4-[fluorobenzylamino]-phenyl) carbamic acid|D-23129	Neurological Diseases	**112.37**
S3698	894-71-3	Nortriptyline hydrochloride	Desitriptyline HCl, ELF-101 hydrochloride, EN-7048 hydrochloride, Desmethylamitriptyline hydrochloride	Neurological Diseases	**112.35**
S5203	3286-46-2	Sulbutiamine		Neurological Disease	**112.28**
S4581	102-76-1	Triacetin	Glycerol triacetate, Glyceryl triacetate, Glycerin triacetate, 1,2,3-Triacetoxypropane	Infection	**112.26**
S5504	84057-95-4	Ropivacaine		Neurological Disease	**112.19**
S5647	87233-62-3	Emedastine Difumarate	Rapimine, Emedastine fumarate	Neuronal Signaling	**112.15**
S5017	2277-92-1	Oxyclozanide	Oxiclozanidum, Zanil, Oxyclozanid, Zanilox	Infection	**112.10**
S7377	9087-70-1	Aprotinin	N/A	Cardiovascular Disease	**112.03**
S3196	115-46-8	Azacyclonol	MER 17, MDL 4829	Neurological Disease	**111.98**
S7854	869886-67-9	Ulixertinib (BVD-523, VRT752271)	N/A	Cancer	**111.98**
S1738	144701-48-4	Telmisartan	BIBR 277	Cardiovascular Disease	**111.94**
S2673	871700-17-3	Trametinib (GSK1120212)	JTP-74057	Cancer	**111.93**
S2794	1190307-88-0	Sofosbuvir (PSI-7977, GS-7977)	N/A	Infection	**111.91**
S3033	274901-16-5	Vildagliptin (LAF-237)	N/A	Metabolic Disease	**111.82**

S4695	81-13-0	D panthenol	Dexpanthenol, D-Panthenol, Pantothenol, Ilopan, D-Pantothenyl alcohol	Others	**111.65**
S1649	139264-17-8	Zolmitriptan	N/A	Neurological Disease	**111.61**
S4567	36589-58-9	Eprodisate disodium	NC-503	Metabolic Disease	**111.61**
S3057	863031-21-4	Azilsartan Medoxomil	TAK-491	Cardiovascular Disease	**111.60**
S3739	112965-21-6	Calcipotriene	Calcipotriol	Metabolic Disease	**111.53**
S3174	1119-34-2	L-Arginine HCl (L-Arg)	N/A	Cardiovascular Disease/Immunology	**111.51**
S3066	357-08-4	Naloxone HCl	N/A	Neuronal Signaling	**111.48**
S4694	122852-69-1	Alosetron Hydrochloride	GR 68755C, GR 68755, GR 68755X, Lotronex, GR-68755, GR68755	Neuronal Signaling	**111.42**
S4030	1405-41-0	Gentamicin Sulfate	NSC-82261, SCH9724	Infection	**111.39**
S1211	99011-02-6	Imiquimod	R-837, S-26308	Cancer/immunology	**111.35**
S2054	4682-36-4	Orphenadrine Citrate	N/A	Neurological Disease	**111.32**
S4330	7279-75-6	Isoetharine Mesylate	N/A	Others	**111.22**
S5243	1092939-17-7	Ruxolitinib Phosphate	INCB018424, INC424	JAK/STAT	**111.20**
S5499	768-94-5	Amantadine	1-Adamantanamine, 1-Adamantylamine, 1-Aminoadamantane	Microbiology	**111.19**
S4284	3858-89-7	Chloroprocaine HCl	N/A	Neurological Diseases	**111.11**
S3027	71720-56-4	Fenoprofen calcium hydrate	N/A	Inflammation	**111.11**
S6003	775304-57-9	Ataluren (PTC124)	N/A	Others	**111.08**
S2031	73151-29-8	Fenticonazole Nitrate	N/A	Infection	**111.07**
P1017	79517-01-4	Octreotide Acetate	0	Others	**111.06**
S1381	96036-03-2	Meropenem	SM 7338	Infection	**111.03**
S1008	606143-52-6	Selumetinib (AZD6244)	ARRY-142886	Cancer	**111.01**
S1400	202138-50-9	Tenofovir Disoproxil Fumarate	GS-1278 Disoproxil Fumarate	Infection	**110.91**
S1801	66357-59-3	Ranitidine Hydrochloride	AH19065	Gastroenterology	**110.83**
S1859	56-53-1	Diethylstilbestrol	Stilbestrol	Cancer	**110.81**
S4552	97-18-7	Bithionol	Actamer	Infection	**110.80**
S5208	86393-32-0	Ciprofloxacin hydrochloride hydrate	Ciloxan, Ceprimax, Oftacilox	Infection	**110.70**

S1117	35943-35-2	Triciribine	NSC 154020, VD-0002, vqd-002	Cancer	**110.70**
S2573	136-47-0	Tetracaine HCl	Amethocalne HCl	Endocrinology	**110.69**
S4833	33564-30-6	Cefoxitin sodium	N/A	Microbiology	**110.55**
S7155	130370-60-4	Batimastat (BB-94)	N/A	Cancer	**110.52**
S4259	163521-08-2	Vilazodone HCl	N/A	Neurological Diseases	**110.47**
S2456	69-09-0	Chlorpromazine HCl	Sonazine	Neurological Disease	**110.44**
S3727	503070-58-4	Vilanterol Trifenate	GW642444M Trifenate	Respiratory Disease	**110.40**
S2789	477600-75-2	Tofacitinib (CP-690550,Tasocitinib)	N/A	Immunology	**110.18**
S1116	827022-32-2	Palbociclib (PD-0332991) HCl	N/A	Cell Cycle	**110.07**
S4070	50-01-1	Guanidine HCl	Aminoformamidine HCl	Neurological Disease	**110.03**
S1688	5593-20-4	Betamethasone Dipropionate	SCH 11460	Diprospan, Diprolene, Diprosone, Diprolene AF	**109.97**
S2471	65-29-2	Gallamine Triethiodide	N/A	Inflammation	**109.97**
S4102	144143-96-4	Eprosartan Mesylate	N/A	Cardiovascular Disease	**109.97**
S4991	2430-27-5	Valpromide	Depamide, Dipropylacetamide, 2-propylpentanamide	Others	**109.96**
S4155	95-25-0	Chlorzoxazone	N/A	Metabolic Disease	**109.96**
S2040	51803-78-2	Nimesulide	N/A	Cardiovascular Disease	**109.92**
S4390	652154-10-4	Brucine sulfate salt hydrate	N/A	Others	**109.90**
S5305	496-67-3	Bromisoval	bromovalerylurea, Isobromyl, Bromaral, BRN 1773255, 2-Bromo-N-carbamoyl-3-methylbutanamide	Others	**109.89**
S4594	58-71-9	Cephalothin	Cefalotin	Infection	**109.86**
S4522	520-45-6	Dehydroacetic acid	Biocide 470F, Methylacetopyronone	Infection	**109.84**
S4162	121-54-0	Benzethonium Chloride	N/A	Neurological Disease	**109.79**
S3146	154-69-8	Tripelennamine HCl	Pyribenzamine HCl	Neurological Disease	**109.77**
S5030	7327-87-9	Dihydralazine sulphate	Nepresol; Depressan; 1,4-Dihydrazinophthalazine sulfate; Hydralazine sulfate	Others	**109.77**
S1762	98-96-4	Pyrazinamide	Pyrazinoic Acid Amide	Infection	**109.71**

S2503	81110-73-8	Racecadotril	Acetorphan	Infection	**109.70**
S5073	120014-06-4	Donepezil	Aricept, Donepezilo	Neuronal Signaling	**109.66**
S5388	330942-05-7	Betrixaban	PRT054021	Protease	**109.65**
S1191	129453-61-8	Fulvestrant	ICI-182780, ZD 9238	Cancer	**109.63**
S2572	3810-74-0	Streptomycin sulfate	N/A	Infection	**109.63**
S1677	56-75-7	Chloramphenicol	Chloromycetin	Infection	**109.61**
S3850	29031-19-4	Glucosamine sulfate	D-Glucosaminesulfate, D-Glucosamine sulphate	Others	**109.60**
S1207	475108-18-0	Tivozanib (AV-951)	KRN-951	Cancer	**109.57**
S1267	918504-65-1	Vemurafenib (PLX4032, RG7204)	RO5185426	Cancer	**109.56**
S4202	152-11-4	Verapamil HCl	N/A	Cardiovascular Disease	**109.55**
S3105	124858-35-1	Nadifloxacin	OPC-7251	Neurological Disease	**109.52**
S4629	569-57-3	Chlorotrianisene	tri-p-anisylchloroethylene, tris(p-methoxyphenyl)chloroethylene, TACE, CTA	Endocrinology	**109.51**
S3008	74711-43-6	Zaltoprofen	N/A	Inflammation	**109.40**
S4219	1649-18-9	Azaperone	NSC 170976	Neurological Disease	**109.38**
S4625	147084-10-4	Alcaftadine	Lastacaft, R89674	Inflammation	**109.31**
S4336	33402-03-8	Metaraminol Bitartrate	Metaradrine Bitartrate	Others	**109.29**
S5254	854001-07-3	Dasatinib hydrochloride	BMS 354825 hydrochloride	Kinase	**109.26**
S7787	148408-66-6	Docetaxel Trihydrate	RP56976 (NSC 628503) Trihydrate	Cancer	**109.23**
S4836	63612-50-0	Nilutamide	RU23908	Endocrinology & Hormones	**109.18**
S4415	59122-46-2	Misoprostol	N/A	Others	**109.14**
S4819	81-07-2	Saccharin	Benzoic sulfimide, O-Sulfobenzimide, O-Benzoic sulfimide, Saccharimide	Others	**109.09**
S7678	936623-90-4	Sacubitril/valsartan (LCZ696)	Sacubitril, Valsartan	Cardiovascular Disease	**109.04**
S7858	16980-89-5	Dibutyryl-cAMP (Bucladesine)	dbcAMP	Others	**109.03**
S5672	3168-01-2	Hydroxyhexamide		Cardiovascular Disease	**109.01**
S1605	91832-40-5	Cefdinir	FK 482, PD 134393, CI-983	Infection	**108.99**
S1399	61036-62-2	Teicoplanin	Teichomycin	Infection	**108.97**
S4504	6112-76-1	6-Mercaptopurine (6-MP) Monohydrate	N/A	Cancer/Immunology	**108.95**
S2233	161796-78-7	Esomeprazole sodium	N/A	Cancer	**108.95**
S5250	206361-99-1	Darunavir	TMC114	Proteases	**108.94**

S3737	163706-36-3	Cangrelor Tetrasodium	AR-C69931MX	Cardiovascular Disease	**108.89**
S5002	162359-56-0	Fingolimod (FTY720) HCl	N/A	Neurological Disease	**108.89**
S1626	26159-34-2	Sodium	RS-3650	Inflammation	**108.86**
S4699	54350-48-0	Etretinate	Tegison, Ethyl etrinoate, Retinoid, Etretinato	Others	**108.80**
S1698	56211-40-6	Torsemide	AC-4464, JDL-464	Cardiovascular Disease	**108.73**
S2736	936091-26-8	Fedratinib (SAR302503, TG101348)	N/A	Cancer	**108.71**
S1336	61718-82-9	Fluvoxamine maleate	MK-264	Neurological Disease	**108.70**
S2036	22839-47-0	Aspartame	SC-18862	Inflammation	**108.66**
S5592	127-47-9	Vitamin A		Others	**108.49**
S4002	654671-77-9	Sitagliptin phosphate monohydrate	MK-0431	Metabolic Disease	**108.47**
S1607	145202-66-0	Rizatriptan Benzoate	MK-462 Benzoate	Neurological Disease	**108.42**
S1380	192725-17-0	Lopinavir	ABT-378	Infection	**108.41**
S5483	554-92-7	Tribenzagan Hydrochloride	Tribenzagan Hydrochloride	Others	**108.28**
S4656	198470-84-7	Parecoxib	SC-69124, Valus-P, Vorth-P	Inflammation	**108.25**
S2562	304-20-1	Hydralazine HCl	N/A	Cardiovascular Disease	**108.24**
S1376	60282-87-3	Gestodene	SH B 331	Endocrinology	**108.10**
S2087	129101-54-8	Rivastigmine Tartrate	ENA 713	Neuronal Signaling	**108.09**
S5360	2557-49-5	Diflorasone	N/A	Immunology & Inflammation	**107.99**
S3013	155148-31-5	Plerixafor 8HCl (AMD3100 8HCl)	JM 3100 8HCl	Cancer	**107.97**
S4221	3562-84-3	Benzbromarone	N/A	Cardiovascular Disease	**107.96**
S1620	635728-49-3	Darunavir Ethanolate	TMC-114, UIC 94017	Infection	**107.84**
S2451	665-66-7	Amantadine HCl	1-adamantanamine HCl	Cardiovascular Disease	**107.82**
P1049	158861-67-7	GHRP-2	0	Others	**107.82**
S2585	980-71-2	Brompheniramine hydrogen	N/A	Inflammation	**107.80**
S1642	555-30-6	Methyldopa	N/A	Cardiovascular Disease/Neurological Disease	**107.73**
S1082	879085-55-9	Vismodegib (GDC-0449)	N/A	Cancer	**107.69**
S5261	67330-25-0	Ufenamate	Butyl flufenamate, Flufenamic acid butyl ester, fenazol	Immunology & Inflammation	**107.69**
S4343	126-27-2	Oxethazaine	Oxethacaine	Others	**107.68**
S3161	99592-39-9	Sertaconazole nitrate	FI-7045	Infection	**107.67**
S1230	146426-40-6	Flavopiridol (Alvocidib)	NSC 649890 HCl,HMR-1275	Alvocidib	**107.67**
S2512	59804-37-4	Tenoxicam	N/A	Inflammation	**107.61**

S4123	26921-17-5	Timolol Maleate	MK-950	Cardiovascular Disease/Neurological Disease	**107.61**
S4928	357336-20-0	Brivaracetam	UCB34714	Others	**107.59**
S2823	865854-05-3	Tideglusib	NP031112, NP-12	Neurological Disease	**107.53**
S4073	6018-19-5	Sodium 4-Aminosalicylate	N/A	Neurological Disease	**107.51**
S5481	15622-65-8	Molindone hydrochloride	N/A	Neuronal Signaling	**107.49**
S4667	73-78-9	Lidocaine hydrochloride	Lidocaine HCL, Lidothesin, Lignocaine hydrochloride, Xyloneural	Cardiovascular Disease	**107.48**
S4389	3818-50-6	Bephenium Hydroxynaphthoate	N/A	Others	**107.43**
S1615	106266-06-2	Risperidone	R-64766	Neurological Disease	**107.42**
S4540	53179-09-2	Sisomicin sulfate	N/A	Infection	**107.32**
S1692	55-98-1	Busulfan	N/A	Cardiovascular Disease	**107.31**
S7765	915769-50-5	Dovitinib (TKI258) Lactate	N/A	Cancer	**107.28**
S2665	52214-84-3	Ciprofibrate	Win-35833	Metabolic Disease	**107.28**
S3045	26328-04-1	Cinepazide maleate	N/A	Cardiovascular Disease	**107.25**
S2113	96946-42-8	Cisatracurium Besylate	N/A	Neurological Disease	**107.22**
S1480	1026785-59-0	Lomibuvir (VX-222, VCH-222)	N/A	Infection	**107.19**
S4136	53716-49-7	Carprofen	N/A	Inflammation	**107.17**
S1512	171596-29-5	Tadalafil	IC351	Cardiovascular Disease	**107.10**
S1786	129497-78-5	Verteporfin	CL 318952	Others	**107.00**
S5033	67915-31-5	Terconazole	Terazol 3, Terazol 7, Triaconazole, Gyno-Terazol	Infection	**106.98**
S1740	93-14-1	Guaifenesin	N/A	Respiratory Disease	**106.94**
S5531	148016-81-3	Doripenem		Infection	**106.92**
S1098	459868-92-9	Rucaparib (AG-014699,PF-01367338) phosphate	N/A	Cancer	**106.91**
S5413	1210344-57-2	Ertugliflozin	PF-04971729	Ion-Channel	**106.87**
S1358	79794-75-5	Loratadine	SCH29851	Inflammation	**106.84**
S4361	28657-80-9	Cinoxacin	N/A	Others	**106.76**
S1756	74011-58-8	Enoxacin	AT-2266, CI919, Pd107779, NSC 629661	Infection	**106.75**
S5627	54301-15-4	Amsacrine hydrochloride		Others	**106.74**
S4288	305-03-3	Chloroambucil	Chlorambucil	Cancer	**106.67**
S2510	21736-83-4	Spectinomycin 2HCl	Actinospectacin	Infection	**106.66**
S4014	101-31-5	Hyoscyamine	Daturine	Gastroenterology	**106.63**
S5228	83701-22-8	sulphate	U-58838	Others	**106.60**

S2576	1218-35-5	Xylometazoline HCl	N/A	Infection	**106.56**
S1044	162635-04-3	Temsirolimus (CCI-779, NSC 683864)	N/A	Cancer	**106.53**
S1385	112885-42-4	Mosapride Citrate	AS-4370	Neurological Disease	**106.49**
S5229	87-33-2	Isosorbide dinitrate	Sorbide nitrate, Sorbidnitrate, Isordil, Nitrosorbide	Others	**106.46**
S3719	577778-58-6	Topiroxostat	N/A	Metabolic Disease	**106.40**
S4751	260779-88-2	Cisapride hydrate	Propulsid, Alimix, Propulsin, Enteropride, Kinestase	Cardiovascular Disease	**106.38**
S1714	95058-81-4	Gemcitabine	LY-188011, NSC 613327	Gemzar	**106.36**
S1719	7481-89-2	Zalcitabine	NSC 606170, Ro 24-2027/000	Infection	**106.35**
S4213	62013-04-1	Dirithromycin	LY-237216, ASE 136	Infection	**106.33**
S3741	22994-85-0	Benznidazole	Radanil	Infection	**106.28**
S1658	115256-11-6	Dofetilide	UK-68798	Cardiovascular Disease	**106.27**
S4541	3380-34-5	Triclosan	Irgasan, Cloxifenolum	Infection	**106.23**
S1896	127-07-1	Hydroxyurea	nci-c04831, nsc32065	Cancer	**106.21**
S4628	57-96-5	(+/-)-Sulfinpyrazone	G-28315, NSC 75925	Others	**106.15**
S1707	107724-20-9	Eplerenone	CGP 30083, SC-66110	Cardiovascular Disease	**106.14**
S4264	31637-97-5	Etofibrate	N/A	Metabolic Disease	**106.12**
S4058	132112-35-7	Ropivacaine HCl	LEA-103 HCl	Neurological Disease	**106.08**
S3144	133099-07-7	Darifenacin HBr	UK-88525	Neurological Disease	**106.03**
S5540	144481-98-1	Landiolol hydrochloride	N/A	others	**105.98**
S8558	1201913-82-7	Tofogliflozin(CSG 452)	N/A	Metabolic Disease	**105.94**
S3180	177834-92-3	Eletriptan HBr	UK-116044	Neurological Disease	**105.92**
S2505	155141-29-0	Rosiglitazone maleate	BRL 49653	Metabolism	**105.88**
S4619	122892-31-3	Itopride hydrochloride	Ganaton, HSR803	Gastroenterology/Neurological Disease	**105.87**
S4368	7421-40-1	Carbenoxolone Sodium	N/A	Others	**105.85**
S8594	150915-40-5	Tirofiban Hydrochloride	MK-383 Hydrochloride	Cardiovascular Disease	**105.85**
S2511	2447-57-6	Sulfadoxine	Sulphadoxine	Infection	**105.85**
S4228	3801-06-7	Fluorometholone Acetate	NSC 47438	Inflammation	**105.82**
S1622	53-03-2	Prednisone	N/A	Immunology	**105.76**
S2615	108341-18-0	Noradrenaline bitartrate monohydrate	Levophed	Neurological Disease	**105.69**
S5205	923288-95-3	Nilotinib hydrochloride	AMN-107 HCl	Angiogenesis	**105.64**

S4565	83-73-8	Diiodohydroxyquinoline	Iodoquinol	Infection	**105.64**
S4100	81161-17-3	Esmolol HCl	ASL8052	Cardiovascular Disease	**105.62**
S4246	1986-47-6	Tranylcypromine (2-PCPA) HCl	SKF-385 HCl	Neurological Disease	**105.61**
S1324	77883-43-3	Doxazosin Mesylate	N/A	Cardiovascular Disease	**105.58**
S4506	59-66-5	Acetazolamide	Diamox	Neurological Disease	**105.52**
S3721	202189-78-4	Bilastine	N/A	Neuronal Signaling	**105.49**
S4311	17140-60-2	Calcium Gluceptate	N/A	Others	**105.46**
S1693	298-46-4	Carbamazepine	NSC 169864	Neurological Disease	**105.45**
S3065	39831-55-5	disulfate	BB-K8	Infection	**105.44**
S3183	549-18-8	Amitriptyline HCl	N/A	Neurological Disease	**105.41**
S7440	1211441-98-3	Ribociclib (LEE011)	N/A	Cancer	**105.39**
S2509	959-24-0	Sotalol HCl	N/A	Neurological Disease	**105.37**
S4218	14028-44-5	Amoxapine	CL 67772	Neurological Disease	**105.34**
S1609	60-56-0	Methimazole	Tapazole, Thiamazole	Endocrinology	**105.24**
S1768	117467-28-4	Cefditoren Pivoxil	ME-1207	Infection	**105.21**
S5492	27220-47-9	Econazole	N/A	Microbiology	**105.19**
S2041	536-43-6	Dyclonine HCl	N/A	Inflammation	**105.12**
S4157	50-63-5	Chloroquine Phosphate	N/A	Immunology/Infection	**105.08**
S2559	50-04-4	Cortisone acetate	NSC 49420	Cancer	**105.05**
S5253	81098-60-4	Cisapride	Kaudalit, Kinestase, Prepulsid, Presid, Pridesia, Propulsid	Neuronal Signaling	**104.94**
S1164	417716-92-8	Lenvatinib (E7080)	N/A	Cancer	**104.91**
S1628	76-25-5	Triamcinolone Acetonide	N/A	Inflammation	**104.87**
S5728	83435-67-0	Delapril Hydrochloride		Others	**104.87**
S4170	91-64-5	Coumarin	N/A	Cardiovascular Disease	**104.85**
S7635	182431-12-5	Lomitapide	N/A	Metabolic Disease	**104.84**
S4171	67-48-1	Choline Chloride	N/A	Neuronal Signaling	**104.75**
S4299	66-76-2	Dicoumarol	Dicumarol	Cardiovascular Disease	**104.71**
S1929	57381-26-7	Irsogladine	N/A	Gastroenterology	**104.68**
S1291	83881-52-1	Cetirizine DiHCl	UCB P071	Inflammation	**104.63**
S1491	21679-14-1	Fludarabine	FaraA, Fludarabinum	F-ara-A, NSC 118218	**104.60**
S3137	54-21-7	Sodium salicylate	N/A	Inflammation	**104.56**
S7156	154039-60-8	Marimastat (BB-2516)		Cancer/Immunology	**104.50**
S3697	138-37-4	Mafenide hydrochloride	4-Aminomethylbenzenesulfonamide hydrochloride	Infection	**104.48**

S1685	63-74-1	Sulfanilamide	N/A	Infection	**104.41**
S3736	114870-03-0	Fondaparinux Sodium	Natural heparin pentasaccharide Sodium	Cardiovascular Disease	**104.39**
S2457	21462-39-5	Clindamycin HCl	N/A	Neurological Disease	**104.38**
S1329	33125-97-2	Etomidate	R16659	Neurological Disease	**104.37**
S1378	941678-49-5	Ruxolitinib (INCB018424)	N/A	Cancer	**104.35**
S4335	5874-97-5	Metaproterenol Sulfate	Orciprenaline Sulfate	Others	**104.30**
S1209	51-21-8	Fluorouracil (5-Fluoracil, 5-FU)	NSC 19893	Cancer	**104.26**
S2497	15500-66-0	Pancuronium dibromide	N/A	Cardiovascular Disease	**104.25**
S1962	122-11-2	Sulphadimethoxine	N/A	Infection	**104.24**
S4121	6101-15-1	Succinylcholine Chloride Dihydrate	Suxamethonium Chloride Dihydrate	Neurological Diseases	**104.16**
S5428	53-60-1	Promazine hydrochloride	Romtiazin hydrochloride, Sinophenin hydrochloride	Neuronal Signaling	**104.14**
S4830	69-79-4	Maltose	maltobiose, malt sugar, Beta-maltose, D-Maltose	Others	**104.14**
S4242	57-09-0	Cetrimonium Bromide (CTAB)	N/A	Infection	**104.09**
S8067	618385-01-6	Vorapaxar	SCH 530348, MK-5348	Cardiovascular Disease/Respiratory Disease	**104.08**
S4574	110-85-0	Piperazine	1,4-Diazacyclohexane, Diethylenediamine, exahydropyrazine, Piperazidine	Infection	**104.06**
S3648	68302-57-8	Amlexanox	AA-673, CHX-3673	Imflammation	**104.05**
S2104	23672-07-3	Levosulpiride	N/A	Neurological Disease	**104.02**
S5009	69304-47-8	Brivudine	BVDU	Infection	**104.00**
S2460	104632-26-0	Pramipexole	SND 919	Mirapexin, Sifrol	**103.97**
S1006	379231-04-6	Saracatinib (AZD0530)	N/A	Cancer	**103.94**
S2290	71939-50-9	Dihydroartemisinin (DHA)	N/A	Infection	**103.94**
S1548	461432-26-8	Dapagliflozin	BMS-512148	Metabolic Disease	**103.93**
S4559	130-16-5	Cloxiquine	5-Chloro-8-quinolinol, Dermofungin|	Infection	**103.87**
S1655	163222-33-1	Ezetimibe	SCH-58235	Cardiovascular Disease	**103.87**
S3655	123171-59-5	Cefepime Dihydrochloride Monohydrate	N/A	Infection	**103.78**
S4556	138-41-0	Carzenide	4-Sulfamoylbenzoic acid	Others	**103.77**

S5507	39133-31-8	Trimebutine maleate	3,4,5-Trimethoxybenzoic Acid 2-(Dimethylamino)-2-phenylbutyl Ester Maleate Salt;	Others	**103.73**
S4673	54048-10-1	Etonogestrel	Implanon, Nexplanon, 3-Oxodesogestrel, 3-keto-Desogestrel	Endocrinology	**103.67**
S4575	51-15-0	Pralidoxime chloride	2-PAM (chloride)	Others	**103.66**
S4554	1083-57-4	Bucetin	N/A	Inflammation	**103.61**
P1087	89213-87-6	Carperitide Acetate		Others	**103.61**
S7684	162520-00-5	Salirasib	Farnesylthiosalicylic acid, FTS	Cancer/Diagnosis	**103.50**
S7205	1229705-06-9	Idasanutlin (RG-7388)	N/A	Cancer	**103.49**
S1511	4618-18-2	Lactulose	N/A	Gastroenterology	**103.48**
S3058	119478-55-6	Danofloxacin Mesylate	CP-76136-27	Infection	**103.46**
S5411	76497-13-7	Sultamicillin	N/A	Microbiology	**103.41**
S4238	481-49-2	Cepharanthine	NSC-623442	Apoptosis	**103.40**
S1743	161973-10-0	NEXIUM (esomeprazole magnesium)	N/A	Gastroenterology	**103.40**
S3167	850-52-2	Altrenogest	A35957, RU2267	Neurological Disease	**103.38**
S1351	70288-86-7	Ivermectin	MK933	Transmembrane Transporters	**103.28**
S7864	64224-21-1	Oltipraz	N/A	Metabolic Disease	**103.21**
S2079	82586-52-5	Moexipril HCl	RS-10085	Gastroenterology	**103.20**
S2328	389-08-2	Nalidixic acid	NSC-82174	Infection	**103.19**
S2028	101831-37-2	Diclazuril	N/A	Infection	**103.15**
S2528	29342-05-0	Ciclopirox	N/A	Batrafen, Loprox, Mycoster, Stieprox, HOE 296b	**103.14**
S2579	30516-87-1	Zidovudine	Azidothymidine, NSC 602670	Cardiovascular Disease	**103.12**
S1729	25812-30-0	Gemfibrozil	CI-719	Cardiovascular Disease	**103.09**
S1881	14222-60-7	Protionamide	Prothionamide, 1321-TH	Infection	**103.08**
S3116	72-14-0	Sulfathiazole	N/A	Infection	**102.99**
S4349	1508-76-5	Procyclidine HCl	N/A	Others	**102.90**
S3178	138890-62-7	Brinzolamide	AL-4862	Neurological Disease	**102.79**
S1888	14484-47-0	Deflazacort	MDL 458	Endocrinology	**102.79**
S4342	51-12-7	Nialamide	N/A	Others	**102.75**
S1734	71125-38-7	Meloxicam	N/A	Inflammation	**102.73**
S4257	59338-87-3	Alizapride HCl	N/A	Neurological Diseases	**102.69**
S1018	405169-16-6	Dovitinib (TKI-258, CHIR-258)	N/A	CHIR-258	**102.68**

S4043	522-48-5	Tetrahydrozoline HCl	N/A	Inflammation	**102.65**
S1789	58-46-8	Tetrabenazine (Xenazine)	N/A	Neurological Disease	**102.63**
S2606	84371-65-3	Mifepristone	RU486, C-1073	Endocrinology	**102.57**
S1359	124750-99-8	Losartan Potassium (DuP 753)	MK 954	Cardiovascular Disease	**102.44**
S2336	65-86-1	Orotic acid (6-Carboxyuracil)	N/A	Metabolic Disease	**102.27**
S1845	51481-61-9	Cimetidine	SKF-92334	Inflammation	**102.17**
S2092	90038-01-0	Detomidine HCl	N/A	Cardiovascular Disease	**102.15**
S5067	114798-26-4	Losartan		Others	**102.10**
S4564	1642-54-2	Diethylcarbamazine citrate	N/A	Infection	**102.03**
S1960	52549-17-4	Pranoprofen	Pyranoprofen	Inflammation	**102.03**
S8116	1420477-60-6	Acalabrutinib (ACP-196)	N/A	Cancer	**102.01**
S2496	82571-53-7	Ozagrel	OKY-046	Others	**101.96**
S2584	25122-46-7	Clobetasol propionate	CGP9555, CCl 4725	Neurological Disease	**101.95**
S4385	78964-85-9	Fosfomycin Tromethamine	Phosphomycin Tromethamine, Phosphonomycin Tromethamine	Others	**101.91**
S4226	13614-98-7	Minocycline HCl	N/A	Infection	**101.85**
S4160	69-57-8	Penicillin G Sodium	Benzylpenicillin Sodium	Infection	**101.70**
S4690	6805-41-0	Escin	Aescin	Inflammation	**101.68**
S3132	127-79-7	Sulfamerazine	RP 2632	Infection	**101.66**
S1436	30123-17-2	Tianeptine sodium	N/A	Neurological Disease	**101.65**
S4166	94-20-2	Chlorpropamide	N/A	Metabolic Disease	**101.65**
S2590	111025-46-8	Pioglitazone	N/A	Metabolism	**101.64**
S1256	106308-44-5	Rufinamide	CGP 33101	Neurological Disease	**101.54**
S1806	51037-30-0	Acipimox	Olbemox	Cardiovascular Disease/Metabolic Disease	**101.53**
S3650	132-98-9	Penicillin V potassium salt	Phenoxymethylpenicillin potassium salt	Infection	**101.48**
S1614	1744-22-5	Riluzole	RP-54274, PK 26124	Neurological Disease	**101.46**
S2567	71-58-9	Medroxyprogesterone acetate	NSC-26386	Medroxyprogesterone 17-acetate, MPA	**101.46**
S2502	6119-47-7	Quinine HCl Dihydrate	N/A	Inflammation	**101.45**
S4675	115103-54-3	Tiagabine	Gabitril, NO050328, NO328, TGB	Gabitril, NO050328, NO328, TGB	**101.44**
S4234	1405-37-4	Capreomycin Sulfate	N/A	Infection	**101.43**
S1111	849217-64-7	Foretinib (GSK1363089)	EXEL-2880,XL-880	Cancer	**101.39**
S2473	84-16-2	Hexestrol	Bibenzyl	Cancer	**101.37**
S1828	5875-06-9	Proparacaine HCl	N/A	Neurological Disease	**101.34**
S3140	101152-94-7	Milnacipran HCl	N/A	Endocrinology	**101.30**

S7625	1038915-73-9	Niraparib (MK-4827) tosylate	Niraparib tosylate, MK 4827 tosylate	Cancer	**101.19**
S3012	444731-52-6	Pazopanib	GW786034	Protein Tyrosine Kinase	**101.16**
S3649	72558-82-8	Ceftazidime	N/A	Infection	**101.14**
S1690	2152-44-5	Betamethasone Valerate	N/A	Inflammation	**101.10**
S4661	145821-59-6	Tiagabine hydrochloride	Gabitril hydrochloride, NO050328 hydrochloride, NO328 hydrochloride, TGB hydrochloride	Gabitril hydrochloride, NO050328 hydrochloride, NO328 hydrochloride, TGB hydrochloride	**101.10**
S4601	130-26-7	Clioquinol	Clioquinol, Iodochlorhydroxyquin, Chinoform	Infection	**101.10**
S4749	59729-32-7	Citalopram HBr	Nitalapram HBr, Prepram HBr, Bonitrile HBr, Lu 10-171 HBr	Neurological Diseases	**101.04**
S1654	50-33-9	Phenylbutazone	Butazolidine	Cancer	**101.03**
S7161	926927-61-9	Motolimod (VTX-2337)	N/A	Cancer	**101.01**
S4017	109-57-9	Allylthiourea	N/A	Metabolic Disease	**101.01**
S4111	343-55-5	Dicloxacillin Sodium	Veracillin, BRL1702	Infection	**100.97**
S1975	129722-12-9	Aripiprazole	OPC-14597	Neurological Disease	**100.93**
S5325	104206-65-7	Nitisinone	N/A	Others	**100.87**
S3018	4394-00-7	Niflumic acid	N/A	Infection	**100.85**
S1379	4759-48-2	Isotretinoin	13-cis retinoic acid	Metabolic Disease	**100.83**
S1972	54965-24-1	Tamoxifen Citrate	ICI 46474 Citrate	Endocrinology	**100.79**
S4117	51-74-1	Histamine Phosphate	N/A	Respiratory Disease	**100.77**
S4074	7632-00-0	Sodium Nitrite	N/A	Neurological Disease	**100.73**
S4282	159989-65-8	Nelfinavir Mesylate	Viracept, AG1343	Infection	**100.62**
S5621	82009-34-5	Cilastatin	N/A	Others	**100.54**
S3681	58-95-7	Vitamin E Acetate	Tocopherol acetate	Others	**100.54**
S2519	550-99-2	Naphazoline HCl	N/A	Neurological Disease	**100.43**
S2446	141505-33-1	Levosimendan	N/A	Cardiovascular Disease	**100.33**
S1893	5959-95-5	D-glutamine	N/A	Others	**100.32**
S4696	3505-38-2	CarbinoxaMine Maleate	N/A	Inflammation	**100.29**
S2199	173334-58-2	Aliskiren Hemifumarate	N/A	Cardiovascular Disease	**100.19**
S1673	317-34-0	Aminophylline	Phyllocontin	Respiratory Disease	**100.14**
S1415	120202-66-6	Clopidogrel	SR-25990C	Cardiovascular Disease	**100.08**

S4669	23239-88-5	Benzocaine hydrochloride	Ethyl 4-aminobenzoate hydrochloride, Benzocaine HCl, Ethyl p-aminobenzoate hydrochloride	Neurological Disease	**100.07**
S1504	479-18-5	Dyphylline	Diprophylline	Respiratory Disease	**100.06**
S1538	402957-28-2	Telaprevir (VX-950)	LY-570310, MP-424	Infection	**100.05**
S4341	58895-64-0	Nalmefene HCl	N/A	Others	**100.05**
S1843	474-25-9	Chenodeoxycholic Acid	Chenodiol	Gastroenterology	**100.02**
S1523	1349796-36-6	Voxtalisib (SAR245409, XL765) Analogue	N/A	Cancer	**99.96**
S2560	17440-83-4	Amiloride HCl dihydrate	N/A	Cardiovascular Disease	**99.90**
S1087	160003-66-7	Iniparib (BSI-201)	NSC-746045, IND-71677	Cancer	**99.88**
S1627	55981-09-4	Nitazoxanide	N/A	Infection	**99.87**
S5015	923604-59-5	Simeprevir	TMC-435, TMC-435350	Proteases	**99.83**
S4088	2135-17-3	Flumethasone	RS-2177, NSC-54702	Endocrinology	**99.65**
S3024	84057-84-1	Lamotrigine	BW-430C	Cancer	**99.58**
S7786	183321-74-6	Erlotinib	CP358774, NSC 718781	Cancer	**99.57**
S8195	1640292-55-2	Oclacitinib?maleate	PF-03394197	Immunology	**99.46**
S4639	913611-97-9	Brexpiprazole	OPC-34712	Neurological Disease	**99.46**
S1984	53885-35-1	Ticlopidine HCl	N/A	Cardiovascular Disease	**99.42**
S4373	67-92-5	Dicyclomine HCl	Dicycloverine HCl	Others	**99.27**
S5070	115074-43-6	Mupirocin calcium	pseudomonic acid A	Infection	**99.26**
S1865	33286-22-5	Diltiazem HCl	RG 83606 HCl	Cardiovascular Disease	**99.11**
S1667	133-67-5	Trichlormethiazide	N/A	Cardiovascular Disease	**99.03**
S7007	606143-89-9	Binimetinib (MEK162, ARRY-162, ARRY-438162)	N/A	Cancer	**99.02**
S4106	57808-65-8	Closantel	R-31520	Infection	**98.91**
S2564	7081-44-9	Cloxacillin Sodium	N/A	Cardiovascular Disease	**98.81**
S1641	58-94-6	Chlorothiazide	N/A	Cardiovascular Disease	**98.73**
S1213	121032-29-9	Nelarabine	506U78	Cancer	**98.73**
S4181	54527-84-3	Nicardipine HCl	RS-69216	Neurological Disease	**98.72**
S4139	5897-18-7	Cyclizine 2HCl	N/A	Inflammation/Neurological Disease	**98.71**
S1988	51-52-5	Propylthiouracil	NSC 6498, NSC 70461	Endocrinology	**98.70**
S2569	61-76-7	Phenylephrine HCl	NCI-c55641	Endocrinology	**98.70**
S1438	97240-79-4	Topiramate	MCN 4853, RWJ 17021	Neurological Disease	**98.63**
S3876	482-89-3	Indigo	Indigotin	Others	**98.62**
S1468	41294-56-8	Alfacalcidol	N/A	Endocrinology	**98.58**

S4048	22232-54-8	Carbimazole	N/A	Infection	**98.57**
S1168	1069-66-5	Valproic acid sodium salt (Sodium valproate)	N/A	Cardiovascular Disease	**98.55**
S2017	91599-74-5	Benidipine HCl	KW-3049	Cardiovascular Disease	**98.54**
S2006	58-14-0	Pyrimethamine	N/A	Immunology	**98.52**
S2900	1004316-88-4	Cobicistat (GS-9350)	N/A	Cancer	**98.51**
S2029	58-96-8	Uridine	NSC 20256	Infection	**98.45**
S2043	41100-52-1	Memantine HCl	N/A	Neurological Disease	**98.42**
S4404	2066-89-9	Pasiniazid	N/A	Others	**98.34**
S4539	69-72-7	Salicylic acid	2-Hydroxybenzoic acid	Cardiovascular Disease/Inflammation	**98.27**
S1672	125-84-8	Aminoglutethimide	BA-16038, NSC-330915	Endocrinology	**98.23**
S1367	69408-81-7	Amonafide	NSC308847,AS1413	Cancer	**98.20**
S3122	1257517-67-1	Amikacin hydrate	N/A	Infection	**98.19**
S4245	134-03-2	Sodium ascorbate	N/A	Endocrinology	**98.01**
S2069	74863-84-6	Argatroban	MCI-9038	Cardiovascular Disease	**97.97**
S3662	29868-97-1	Pirenzepine dihydrochloride	N/A	Gastroenterology	**97.97**
S5385	170105-16-5	Imidafenacin		Neurological Disease	**97.95**
S1370	120410-24-4	Biapenem	L-627, LJC10627	Infection	**97.92**
S4052	56391-57-2	Netilmicin Sulfate	N/A	Infection	**97.90**
S4172	123-03-5	Cetylpyridinium Chloride	N/A	Infection	**97.90**
S1916	127-69-5	Sulfisoxazole	NU-445	Infection	**97.83**
S1950	1115-70-4	Metformin HCl	N/A	Metabolic Disease	**97.69**
S3730	1665-48-1	Metaxalone	Skelaxin, Methaxalonum, Zorane	Neurological Diseases	**97.68**
S2062	1953-02-2	Tiopronin	N/A	Cardiovascular Disease	**97.61**
S3050	135729-62-3	Palonosetron HCl	RS 25259, RS 25259 197	Neurological Disease	**97.61**
S5655	93413-69-5	Venlafaxine	Wy 45030	Neuronal Signaling	**97.57**
S3742	81-25-4	Cholic acid	N/A	Metabolic Disease	**97.56**
S1645	22071-15-4	Ketoprofen	RP-19583	Inflammation	**97.52**
S1229	75607-67-9	Fludarabine Phosphate	F-ara-A (NSC 312887) Phosphate	Cancer	**97.52**
S1445	68291-97-4	Zonisamide	CI-912	Neurological Disease	**97.49**
S4599	120-51-4	Benzyl benzoate	Ascabiol, Novoscabin, Scabitox, Benzoic acid benzyl ester	Infection	**97.46**
S3070	7491-74-9	Piracetam	UCB 6215	Neurological Disease	**97.43**
S4251	92-84-2	Phenothiazine	ENT 38	Neuronal Signaling	**97.37**

S1794	49562-28-9	Fenofibrate	N/A	Cardiovascular Disease	**97.36**
S5539	106730-54-5	Olprinone		Cardiovascular Disease	**97.35**
S1439	53902-12-8	Tranilast	SB 252218	Respiratory Disease	**97.30**
S1959	13710-19-5	Tolfenamic Acid	N/A	Inflammation	**97.26**
S2550	124937-52-6	Tolterodine tartrate	PNU-200583E	Neurological Disease	**97.16**
S1398	3056-17-5	Stavudine (d4T)	BMY-27857, Sanilvudine, NSC 163661	Infection	**97.15**
S4011	99464-64-9	Ampiroxicam	CP 65703	Cardiovascular Disease	**97.14**
S4930	282526-98-1	Cetilistat	ATL962	Metabolism	**97.05**
S1647	58-61-7	Adenosine	N/A	Cardiovascular Disease	**96.92**
S5007	681492-22-8	Delamanid	OPC-67683	Infection	**96.91**
S2601	21187-98-4	Gliclazide	N/A	Neurological Disease	**96.81**
S3973	3211-76-5	L-SelenoMethionin	N/A	Immunology & Inflammation	**96.80**
S4294	614-39-1	Procainamide HCl	N/A	Cardiovascular Disease	**96.79**
S7550	55224-05-0	Erythromycin Cyclocarbonate	N/A	Infection	**96.79**
S4646	126544-47-6	Ciclesonide	Ciclesonide, Alvesco, Omnaris, RPR251526, Zetonna	Inflammation/Neurological Disease	**96.77**
S1509	70458-96-7	Norfloxacin	MK-0366	Infection	**96.76**
S1235	112809-51-5	Letrozole	CGS 20267	Endocrinology	**96.74**
S4648	24868-20-0	Dantrolene sodium hemiheptahydrate	Dantrolene sodium salt, Dantrium, Sodium dantrolene	Neurological Disease	**96.48**
S5280	36304-84-4	Dimemorfan phosphate		Others	**96.45**
S1790	80621-81-4	Rifaximin	N/A	Infection	**96.45**
S4267	13739-02-1	Diacerein	N/A	Inflammation	**96.43**
S1940	100986-85-4	Levofloxacin	Fluoroquinolone	Levaquin, Tavanic	**96.40**
S5414	59198-70-8	Diflucortolone valerate	Afusona, Diflucortolone 21-valerate	Others	**96.38**
S1883	54-42-2	Idoxuridine	NSC 39661, SKF 14287	Infection	**96.36**
S2625	901119-35-5	Fostamatinib (R788)	N/A	Immunology	**96.33**
S4543	13171-25-0	Trimetazidine dihydrochloride	Yoshimilon, Kyurinett, Vastarel F	Cardiovascular Disease	**96.31**
S3150	23964-57-0	Articaine HCl	Ultracaine	Neurological Disease	**96.21**
S4174	527-07-1	Sodium Gluconate	527-07-1	Others	**96.21**
S2098	153559-49-0	Bexarotene	LGD1069	Cardiovascular Disease	**96.17**
S1992	80474-14-2	Fluticasone propionate	CCI-187881	Inflammation	**96.16**
S2522	51-43-4	L-Adrenaline	Epinephrine	Cardiovascular Disease	**96.16**
S2542	834-28-6	Phenformin HCl	N/A	Metabolic Disease	**96.14**

S1880	93793-83-0	Roxatidine Acetate HCl	N/A	Gastroenterology	**96.08**
S1610	17560-51-9	Metolazone	SR 720-22	Cardiovascular Disease	**96.07**
S4256	33386-08-2	Buspirone HCl	N/A	Neurological Disease	**96.00**
S5729	6064-83-1	Fosfosal		Others	**95.98**
S5626	63449-41-2	Benzalkonium chloride	N/A	Immunology	**95.97**
S2515	330808-88-3	Vardenafil HCl Trihydrate	BAY38-9456	Others	**95.86**
S2091	63659-18-7	Betaxolol	SL 75212	Neurological Disease	**95.84**
S1907	443-48-1	Metronidazole	N/A	Infection	**95.78**
S4660	596-51-0	Glycopyrrolate	Glycopyrrolate bromide, Glycopyrronium bromide, Robinul, Gastrodyn	Neurological Disease	**95.77**
S2032	90098-04-7	Rebamipide	OPC-12759	Gastroenterology	**95.73**
S5581	144689-63-4	Olmesartan		Cardiovascular Disease	**95.70**
S4653	70-26-8	L-Ornithine	(S)-2,5-Diaminopentanoic acid	Others	**95.68**
S5322	6223-35-4	Sodium gualenate	Guaiazulenesulfonate sodium	Immunology & Inflammation	**95.67**
S1391	28721-07-5	Oxcarbazepine	GP47680	Neurological Disease	**95.66**
S1007	808118-40-3	Roxadustat (FG-4592)	ASP1517	Others	**95.64**
S3080	269055-15-4	Etravirine (TMC125)	R-165335	Neurological Disease	**95.56**
S1732	53-19-0	Mitotane	NCI-C04933	Cancer	**95.55**
S4827	7773-01-5	Manganese chloride	N/A	Others	**95.54**
S2568	1405-10-3	Neomycin sulfate	N/A	Infection	**95.44**
S3748	77337-73-6	Acamprosate Calcium	N-Acetylhomotaurine Calcium, calcium acetylhomotaurinate	Neurological Diseases	**95.43**
S3711	5749-67-7	Carbasalate Calcium	Iromin, Alcacyl, Omegin, Rheomin, Solupsan	Inflammmation	**95.42**
S1839	773-76-2	Chloroxine	N/A	Infection	**95.39**
S7492	1047634-65-0	Uprosertib (GSK2141795)	GSK795	Cancer	**95.34**
S1346	9041-08-1	Heparin sodium	N/A	Cardiovascular Disease	**95.30**
S1244	850879-09-3	Amuvatinib (MP-470)	HPK 56	Cancer	**95.25**
S2124	25990-60-7	Xylose	N/A	Metabolic Disease	**95.16**
S4727	298-57-7	Cinnarizine	Stugeron|Dimitronal|Stutgin|Cinarizine	Neurological Disease	**95.15**
S5025	164650-44-6	Efinaconazole	KP-103	Infection	**95.15**
S1713	36322-90-4	Piroxicam	CP 16171	Inflammation	**95.05**
S4056	224452-66-8	Retapamulin	SB-275833	Neurological Disease	**95.02**

S3067	3697-42-5	Chlorhexidine?2 HCl	NSC-185	Infection	**94.89**
S1494	862507-23-1	Ralimetinib (LY2228820)	N/A	Cancer/Inflammation	**94.87**
S4010	34381-68-5	Acebutolol HCl	N/A	Neurological Disease	**94.78**
S4169	108605-62-5	Teriflunomide	A77 1726, HMR-1726	Immunology	**94.77**
S4976	152-47-6	Sulfalene(SMPZ)	Butadiene sulfone, 3-Sulfolene	Others	**94.71**
S5232	1256589-74-8	Alectinib hydrochloride	AF802 hydrochloride	Protein Tyrosine Kinase	**94.71**
S1200	2353-33-5	Decitabine	Deoxycytidine	Cardiovascular Disease	**94.66**
S1990	404-86-4	Capsaicin(Vanilloid)	Qutenza, Vanilloid	Others	**94.66**
S1706	134678-17-4	Lamivudine	GR109714X	Infection	**94.62**
S4067	30652-11-0	Deferiprone	CP20	Others	**94.59**
S4518	88-04-0	Chloroxylenol	N/A	Infection	**94.58**
S5083	37339-90-5	Lentinan	A823605, Bromoduline	Immunology & Inflammation	**94.51**
S1258	150322-43-3	Prasugrel	CS-747, LY640315	Effient, Efient, Prasita	**94.50**
S3037	190786-44-8	Bepotastine Besilate	TAU 284	Cancer	**94.46**
S4416	521-78-8	Trimipramine Maleate	N/A	Others	**94.45**
S4523	5490-27-7	Dihydrostreptomycin sulfate	N/A	Infection	**94.41**
S5240	857890-39-2	lenvatinib Mesylate	N/A	Protein Tyrosine Kinase	**94.33**
S1971	65141-46-0	Nicorandil	SG-75	Cardiovascular Disease	**94.23**
S2230	700874-72-2	Galunisertib (LY2157299)	N/A	Cancer	**94.23**
S1497	146464-95-1	Pralatrexate	N/A	Metabolic Disease	**94.10**
S1958	68373-14-8	Sulbactam	N/A	Infection	**94.10**
S1483	133454-47-4	Iloperidone	HP873	Neurological Disease	**94.08**
S1909	93957-55-2	Fluvastatin Sodium	XU-62-320	Cardiovascular Disease	**94.07**
S5486	426-13-1	Fluorometholone	Oxylone	Endocrinology & Hormones	**94.05**
S1137	59937-28-9	Malotilate	NKK105	Metabolic Disease	**93.96**
S2902	941685-37-6	S-Ruxolitinib (INCB018424)	N/A	Cancer	**93.96**
S4204	652-67-5	Isosorbide	N/A	Cardiovascular Disease	**93.92**
S1330	25451-15-4	Felbamate	ADD-03055	Neurological Disease	**93.87**

S5487	62893-20-3	Cefoperazone sodium	sodium;(6R,7R)-7-[[(2R)-2-[(4-ethyl-2,3-dioxopiperazine-1-carbonyl)amino]-2-(4-hydroxyphenyl)acetyl]amino]-3-[(1-methyltetrazol-5-yl)sulfanylmethyl]-8-oxo-5-thia-1-azabicyclo[4.2.0]oct-2-ene-2-carboxylate.	Others	**93.87**
S2524	630-93-3	Phenytoin Sodium	Diphenylhydantoin Sodium	Neurological Disease	**93.86**
S3728	1350514-68-9	Grazoprevir	MK5172	Infection	**93.84**
S4721	142-47-2	L-Glutamic acid monosodium salt	Monosodium glutamate|MSG	Others	**93.77**
S3755	107-43-7	Betaine	trimethylglycine, glycine betaine, lycine, oxyneurine	Others	**93.74**
S4196	6402-23-9	Ethacridine lactate monohydrate	N/A	Infection	**93.74**
S3061	55-31-2	Epinephrine HCl	Adrenaline	Neuronal Signaling	**93.67**
S4080	396-01-0	Triamterene	SKF8542	Inflammation	**93.63**
S5039	18699-02-0	Actarit	4-acetylaminophenylacetic acid	Immunology & Inflammation	**93.61**
S1247	75706-12-6	Leflunomide	HWA486	Inflammation	**93.59**
S1751	122795-43-1	Gadodiamide Hydrate	N/A	Diagnosis	**93.58**
S5266	49763-96-4	Stiripentol	BCX2600	Others	**93.50**
S7280	1229194-11-9	Edoxaban	DU-176b	Cardiovascular Disease	**93.40**
S4851	209860-87-7	Tafluprost	AFP-168	Others	**93.37**
S4182	965-52-6	Nifuroxazide	N/A	Infection	**93.36**
S2499	63-92-3	Phenoxybenzamine HCl	NSC 37448, NCI-c01661	Endocrinology	**93.35**
S2680	936563-96-1	Ibrutinib (PCI-32765)	N/A	Neurological Disease	**93.30**
S7204	168555-66-6	Fosbretabulin (Combretastatin A4 Phosphate (CA4P)) Disodium	N/A	Cancer	**93.19**
S5157	89-83-8	Thymol	2-isopropyl-5-methylphenol, IPMP	Immunology & Inflammation	**93.17**
P1085	4474-91-3	Angiotensin II human Acetate		Cardiovascular Disease	**93.13**
S4144	137-88-2	Amprolium HCl	N/A	Metabolic Disease	**93.12**

S3935	2444-46-4	Nonivamide	Pseudocapsaicin; Pelargonic acid vanillylamide; Nonanoic acid vanillylamide	Metabolism	**93.08**
S4285	128607-22-7	Ospemifene	N/A	Others	**93.07**
S4398	34552-84-6	Isoxicam	N/A	Others	**93.07**
S1017	288383-20-0	Cediranib (AZD2171)	NSC-732208	Cancer	**93.02**
S7775	254750-02-2	Emricasan	IDN-6556, PF 03491390, PF-03491390	Inflammation	**92.96**
S2475	152459-95-5	Imatinib (STI571)	CGP057148B, ST-1571	Neurological Disease	**92.81**
S4051	42924-53-8	Nabumetone	BRL-14777	Inflammation	**92.72**
S1957	144-82-1	Sulfamethizole	N/A	Infection	**92.70**
S1408	165800-03-3	Linezolid	PNU-100766	Infection	**92.70**
S1721	446-86-6	Azathioprine	N/A	Immunology	**92.69**
S3055	405165-61-9	Besifloxacin HCl	N/A	Infection	**92.65**
S4351	90274-24-1	Ractopamine HCl	N/A	Others	**92.63**
S2667	1051375-16-6	Dolutegravir (GSK1349572)	S/GSK1349572	Infection	**92.63**
S8057	937272-79-2	Pacritinib (SB1518)	N/A	Cancer	**92.61**
S1952	298-81-7	Methoxsalen	Xanthotoxin, NCI-C55903	Inflammation	**92.53**
S8138	1154028-82-6	Molidustat (BAY 85-3934)	N/A	Others	**92.48**
S3724	1377049-84-7	Velpatasvir	GS-5816	Infection	**92.44**
S4334	32672-69-8	Mesoridazine Besylate	N/A	Others	**92.40**
S1904	70356-09-1	Avobenzone	Butyl methoxydibenzoyl methane, BF2AVB	Others	**92.36**
S3747	55-03-8	Levothyroxine sodium	L-T4 sodium, L-Thyroxine sodium, LT4 sodium	Endocrinology	**92.26**
S1046	443913-73-3	Vandetanib (ZD6474)	N/A	Cancer	**92.20**
S1467	54573-75-0	Doxercalciferol	1α-hydroxyvitamin D2	Endocrinology	**92.20**
S5217	200815-49-2	Arformoterol Tartrate	(R,R)-Formoterol	Others	**92.16**
S5710	18683-91-5	Ambroxol		Others	**92.03**
S8048	1257044-40-8	Venetoclax (ABT-199, GDC-0199)	N/A	Cancer	**91.95**
S4817	29122-68-7	Atenolol	Tenormin, Normiten, Blokium	Neuronal Signaling	**91.95**
S4392	2181-04-6	Potassium Canrenoate	Canrenoic Acid Potassium Salt	Others	**91.86**
S4012	100643-71-8	Desloratadine	SCH-34117, NSC 675447	Cardiovascular Disease	**91.85**
S1441	99300-78-4	Venlafaxine HCl	N/A	Neurological Disease	**91.80**
S5703	610309-89-2	Carvedilol Phosphate		Others	**91.79**
S1150	33069-62-4	Paclitaxel	NSC 125973	Cancer	**91.74**
S4626	77-67-8	Ethosuximide	N/A	Neurological Disease	**91.72**

S5497	96-83-3	Iopanoic acid	Acidum iopanoicum, Iodopanoic acid	Others	**91.68**
S3148	138926-19-9	Ibandronate sodium	BM-21.0955	Metabolic Disease	**91.66**
S4078	61-68-7	Mefenamic Acid	CI 473，CN-35355	Cardiovascular Disease	**91.63**
S7781	557795-19-4	Sunitinib	SU11248	Cancer	**91.60**
S2506	80214-83-1	Roxithromycin	Roxl-150	Infection	**91.58**
S1823	34597-40-5	Fenoprofen Calcium	N/A	Nalfon	**91.47**
S2537	3366-95-8	Secnidazole	PM 185184, RP 14539	Infection	**91.45**
S5359	64872-76-0	Butoconazole	N/A	Microbiology	**91.39**
S1718	142340-99-6	Adefovir Dipivoxil	N/A	Infection	**91.37**
S1995	366-70-1	Procarbazine HCl	NSC-77213 HCl	Cancer	**91.31**
S1741	72559-06-9	Rifabutin	LM427, Ansamycin	Infection	**91.29**
S5714	367514-87-2	lurasidone	SM-13496	Neuronal Signaling	**91.28**
S3750	532-32-1	Sodium benzoate	Benzoic acid sodium salt	Neurological Diseases	**91.19**
S4579	108-46-3	Resorcinol	m-dihydroxybenzene, 3-hydroxyphenol	Infection	**91.17**
S4640	878672-00-5	Lesinurad	RDEA 594, RDEA-594, RDEA594	Metabolic Disease	**91.16**
S2516	23076-35-9	Xylazine HCl	N/A	Cardiovascular Disease	**91.15**
S5215	136470-78-5	Abacavir	N/A	Microbiology	**91.12**
S1977	91296-87-6	Sarafloxacin HCl	A-56620 HCl	Infection	**91.12**
S3751	6591-63-5	Quinidine sulfate	Chinidin Sodium, Pitayine Sodium, β-quinine Sodium, (+)-quinidine Sodium	Cardiovascular Disease	**91.09**
S4183	1263-89-4	Paromomycin Sulfate	N/A	Infection	**91.08**
S1289	61422-45-5	Carmofur	HCFU	Cancer	**91.08**
S5032	59-47-2	Mephenesin	Decontractyl, Cresoxydiol, Memphenesin, Mephedan	Neuronal Signaling	**91.05**
S3891	1617-90-9	Vincamine	Angiopac, Devincan, Equipur, Minorin, Novicet, Oxybral, Perval, Sostenil, Tripervan	Others	**90.93**
S1681	89-57-6	Mesalamine	5-Aminosalicylic acid	Inflammation	**90.89**
S4408	23249-97-0	Procodazole	N/A	Others	**90.88**

S4845	117976-89-3	Rabeprazole	N/A	Transmembrane Transporters	**90.87**
S1983	2922-28-3	Adenine HCl	N/A	Others	**90.76**
S8183	706782-28-7	Pimavanserin	ACP-103	Neurological Disease	**90.75**
S4658	1435-55-8	Hydroquinidine	Dihydroquinidine, Hydroconchinine, Hydroconquinine, Dihydroquinine	Cardiovascular Disease	**90.72**
S4211	151767-02-1	Montelukast Sodium	MK-0476	Respiratory Disease	**90.64**
S3637	98753-19-6	Cefpirome sulfate	N/A	Infection	**90.52**
S1243	138112-76-2	Agomelatine	S20098	Neurological Disease	**90.51**
S2491	39562-70-4	Nitrendipine	Bayotensin	Neurological Disease	**90.48**
S1908	13311-84-7	Flutamide	SCH-13521	Cancer	**90.46**
S4358	2062-78-4	Pimozide	N/A	Others	**90.45**
S1501	128794-94-5	Mycophenolate Mofetil	RS61443	Immunology	**90.36**
S3951	1401-55-4	Tannic acid	Gallotannic acid	Immunology & Inflammation	**90.28**
S2501	22204-24-6	Pyrantel Pamoate	Pyrantel Embonate	Infection	**90.25**
S4562	81-23-2	Dehydrocholic acid	Decholin, Dehystolin, Felacrinos, Sanocholen	Gastroenterology	**90.21**
S8241	854107-55-4	Ponesimod	ACT-128800	Immunology	**90.10**
P1063	151126-32-8	Pramlintide Acetate	N/A		**90.09**
S2791	425637-18-9	Sotrastaurin	AEB071	Cancer	**89.99**
S4638	54024-22-5	Desogestrel	Cerazette, Desogen, Desogestrelum, Org-2969	Endocrinology	**89.98**
S5293	55661-38-6	Nimustine Hydrochloride	Nidran hydrochloride	DNA Damage	**89.98**
S4652	4065-45-6	Sulisobenzone	Benzophenone-4, Sungard	Others	**89.95**
S1857	2809-21-4	Etidronate	N/A	Metabolic Disease	**89.93**
S4697	945667-22-1	Saxagliptin hydrate	BMS-477118 hydrate, Onglyza hydrate, BMS 477118 hydrate, BMS477118 hydrate	Metabolic Disease	**89.92**
S1903	15307-79-6	Diclofenac Sodium	GP 45840	Voltaren, Solaraze, Ecofenac	**89.88**
S2074	15574-49-9	Mecarbinate	Dimecarbin	Cardiovascular Disease	**89.86**
S4624	695-53-4	5,5-Dimethyloxazolidine-2,4-dione	Dimethadione, Dimethyloxazolidinedione, Dimethadion	Neurological Disease	**89.84**
S3714	1025967-78-5	Lifitegrast	SAR1118	Others	**89.82**

S4156	64-72-2	Chlortetracycline HCl	N/A	Infection	**89.76**
S4714	2216-51-5	(-)-Menthol	L-Menthol|Levomenthol|Menthomenthol|Menthacamphor	Respiratory Disease	**89.75**
S1982	321-30-2	Adenine sulfate	N/A	DNA Damage	**89.70**
S5210	1981-58-4	Sulfamethazine Sodium Salt	Sulfadimethyldiazine Sodium Salt	Infection	**89.64**
S3104	119637-67-1	Moguisteine	N/A	Respiratory Disease	**89.61**
S4382	59-33-6	Pyrilamine Maleate	Mepyramine Maleate	Others	**89.52**
S5392	96-88-8	Mepivacaine		Neurological Disease	**89.42**
S1833	64872-77-1	Butoconazole nitrate	RS-35887	Infection	**89.42**
S5447	6138-79-0	Triprolidine Hydrochloride	N/A	Neuronal Signaling	**89.38**
S2086	148849-67-6	Ivabradine HCl	S 16257-2	Neurological Disease	**89.21**
S3756	119-36-8	Methyl salicylate	2-Carbomethoxyphenol, Betula oil, Gaultheria oil, Methyl 2-hydroxybenzoate, Natural wintergreen oil	Transmembrane Transporters	**89.20**
S5309	479-92-5	Propyphenazone (4-Isopropylantipyrine)	Isopropylantipyrine, Isopropyrine	Others	**89.19**
S4685	154598-52-4	Efavirenz	Sustiva, Stocrin, DMP-266, DMP 266	Infection	**89.18**
S4249	2295-58-1	Flopropione	N/A	Neurological Diseases	**89.17**
S1644	59-87-0	Nitrofural	Nitrofurazone	Infection	**89.14**
S2078	76824-35-6	Famotidine	MK208	Cardiovascular Disease	**89.11**
S1344	93479-97-1	Glimepiride	N/A	Metabolic Disease	**89.11**
S5397	55-56-1	Chlorhexidine		Infection	**89.11**
S7975	259793-96-9	Favipiravir (T-705)	N/A	Infection	**88.88**
S5241	388082-78-8	Lapatinib ditosylate monohydrate	Tykerb Ditosylate	Others	**88.87**
S3176	5579-84-0	Betahistine 2HCl	PT-9	Neurological Disease	**88.86**
S4837	50847-11-5	Ibudilast	KC-404, AV411, MN166	Metabolism	**88.81**
P1023	79561-22-1	Alarelin Acetate	Glp-His-Trp-Ser-Tyr-D-Ala-Leu-Arg-Pro-NHEt	Angiogenesis	**88.69**
S4578	25301-02-4	Tyloxapol	Triton WR1339	Respiratory Disease	**88.67**
S1437	64461-82-1	Tizanidine HCl	DS 103-282	Neurological Disease	**88.57**
S5657	153773-82-1	Ertapenem sodium	MK826	Microbiology	**88.56**

S2154	211915-06-9	Dabigatran Etexilate	BIBR-1048	Cardiovascular Disease	**88.38**
S4022	57-66-9	Probenecid	Benemid	Metabolic Disease	**88.32**
S4832	26171-23-3	Tolmetin	Tolectin	Immunology & Inflammation	**88.31**
S1156	154361-50-9	Capecitabine	RO 09-1978	Cancer	**88.18**
S1897	7681-11-0	Potassium Iodide	N/A	Endocrinology	**88.16**
S4247	179474-85-2	Prucalopride Succinate	N/A	Gastroenterology	**88.15**
S4384	91296-86-5	Difloxacin HCl	N/A	Others	**88.15**
S2721	75530-68-6	Nilvadipine	ARC029, FR34235	Cardiovascular Disease	**88.14**
S1159	888216-25-9	Ganetespib (STA-9090)	N/A	Cancer	**88.11**
S2555	81103-11-9	Clarithromycin	A-56268	Neurological Disease	**88.10**
S4035	50-14-6	Vitamin D2	Ergocalciferol	Endocrinology	**88.09**
S7284	1374640-70-6	Rociletinib (CO-1686, AVL-301)	CNX-419	Cancer/Immunology	**88.05**
S8030	110078-46-1	Plerixafor (AMD3100)	JM 3100	GPCR & G Protein	**87.97**
S5048	26016-99-9	Fosfomycin Disodium	Phosphonemycin Disodium	Microbiology	**87.93**
S1856	56392-17-7	Metoprolol Tartrate	CGP 2175E	Cardiovascular Disease	**87.89**
S1646	74103-07-4	Ketorolac	N/A	Neurological Disease	**87.87**
S2693	864814-88-0	Resminostat	RAS2410	Cancer	**87.81**
S1826	95734-82-0	Nedaplatin	N/A	Cancer	**87.78**
S2109	89396-94-1	Imidapril HCl	N/A	Cardiovascular Disease	**87.72**
S4122	108050-54-0	Tilmicosin	EL 870	Infection	**87.55**
S4063	67-97-0	cholecalciferol (Vitamin D3)	Cholecalciferol	Others	**87.53**
S4068	19387-91-8	Tinidazole	CP12574	Infection	**87.51**
S2129	13189-98-5	Fudosteine	N/A	Respiratory Disease	**87.49**
S8146	50-07-7	Mitomycin C	N/A	Infection	**87.49**
S3672	61270-78-8	Cefonicid sodium	Monocid sodium	Infection	**87.48**
S3124	1177-87-3	Dexamethasone Acetate	NSC 39471	Inflammation	**87.43**
S1065	957054-30-7	Pictilisib (GDC-0941)	RG7321	Cancer	**87.42**
S1283	85650-56-2	Asenapine maleate	Org 5222	Neurological Disease	**87.38**
S7303	500287-72-9	Rilpivirine	R278474, TMC278	Infection	**87.36**
S3735	869113-09-7	Umeclidinium bromide	N/A	Respiratory Disease	**87.31**
S3963	6899-04-3	DL-Glutamine	Glutamin, 2-amino-4-carbamoylbutanoic acid	Others	**87.21**
S3945	339-72-0	L-Cycloserine	Levcycloserine, Levcicloserina, Levcycloserinum, (-)-Cycloserine, (S)-Cycloserine	Metabolism	**87.12**
S4812	68401-81-0	Ceftizoxime	N/A	Microbiology	**87.12**

S4025	51-56-9	Homatropine Bromide	N/A	Neurological Disease	**87.07**
S1193	50-35-1	Thalidomide	N/A	Immunology	**87.02**
S1178	755037-03-7	Regorafenib (BAY 73-4506)	Fluoro-Sorafenib	Cancer	**87.01**
S1328	41340-25-4	Etodolac	N/A	Inflammation	**86.98**
S5450	64506-49-6	Sofalcone		Infection Inflammation/Immunology	**86.98**
S1299	50-91-9	Floxuridine	Deoxyfluorouridine, FDUR, NSC 27640	Cancer	**86.93**
S1730	26807-65-8	Indapamide	N/A	Cardiovascular Disease	**86.87**
S2453	14919-77-8	Benserazide HCl	Ro-4-4602	Neurological Disease	**86.80**
S3053	123524-52-7	Azelnidipine	UR-12592	Neurological Disease	**86.79**
S2065	118288-08-7	Lafutidine	FRG-8813	Infection	**86.71**
S8016	1260141-27-2	Vonoprazan Fumarate (TAK-438)	N/A	Gastroenterology	**86.70**
S1986	1104-22-9	Meclizine 2HCl	NSC 28728	Neurological Disease	**86.64**
S1202	164656-23-9	Dutasteride	GI198745, GG-745	Endocrinology	**86.63**
S3667	74431-23-5	Imipenem	N/A	Infection	**86.52**
S3046	147403-03-0	Azilsartan	TAK-536	Neurological Disease	**86.49**
S1515	929016-96-6	Pracinostat (SB939)	N/A	Cancer	**86.45**
S2581	82586-55-8	Quinapril HCl	CI-906, PD-109452-2	Cardiovascular Disease	**86.44**
S3074	77-36-1	Chlorthalidone	Chlortalidone	Others	**86.41**
S5136	1492-18-8	Calcium folinate	Leucovorin calcium, Folinic acid calcium salt, Wellcovorin	Others	**86.34**
S2574	64-75-5	Tetracycline HCl	NCI-c55561	Infection	**86.33**
S9327	35700-23-3	Carboprost		Others	**86.30**
S4641	856867-55-5	Tedizolid Phosphate	TR-701FA	Infection	**86.30**
S1506	107133-36-8	Perindopril Erbumine	S9490-3	Cardiovascular Disease	**86.27**
S1633	107753-78-6	Zafirlukast	ICI-204219	Inflammation	**86.22**
S5515	6130-64-9	Penicillin G Procaine	Procaine benzylpenicillin	Microbiology	**86.14**
S1913	1508-75-4	Tropicamide	N/A	Neurological Disease	**86.08**
S1608	101-26-8	Pyridostigmine Bromide	N/A	Cardiovascular Disease	**86.06**
S5477	51-77-4	Gefarnate		Inflammation/Immunology	**86.02**
S2563	7240-38-2	Oxacillin sodium monohydrate	Methicillin	Infection	**85.98**
S4034	62-97-5	Diphemanil Methylsulfate	N/A	Neurological Disease	**85.95**
S1807	59277-89-3	Aciclovir	Acyclovir	Infection	**85.90**
S1737	50-24-8	Prednisolone	N/A	Decaprednil, Predonine	**85.84**
S8565	1226781-44-7	Omarigliptin (MK-3102)	N/A	Metabolic Disease	**85.75**
S4240	562-10-7	Doxylamine Succinate	N/A	Neurological Disease	**85.69**

S1010	656247-17-5	Nintedanib (BIBF 1120)	Intedanib	Respiratory disease	**85.66**
S5079	486460-32-6	Sitagliptin	MK-0431	Proteases	**85.66**
S7218	848141-11-7	Alvelestat (AZD9668)	Avelestat	Metabolic Disease	**85.63**
S2024	34580-14-8	Ketotifen Fumarate	N/A	Neurological Disease	**85.62**
S1280	71675-85-9	Amisulpride	DAN-2163	Neurological Disease	**85.61**
S1891	28860-95-9	Carbidopa	N/A	Neurological Disease	**85.60**
S2051	62571-86-2	Captopril	SQ 14225	Metabolic Disease	**85.59**
S7785	357166-30-4	Pemetrexed Disodium Hydrate	LY-231514 Disodium Hydrate	Cancer	**85.52**
S5637	95789-30-3	Cefotiam Hexetil Hydrochloride		Others	**85.47**
S4381	62-68-0	Proadifen HCl	SKF 525-A HCl	Others	**85.43**
P1015	50-57-7	[illegible] Acetate	0	Others	**85.39**
S2150	698387-09-6	Neratinib (HKI-272)	N/A	Cancer	**85.39**
S1035	635702-64-6	Pazopanib HCl (GW786034 HCl)	N/A	Cancer	**85.37**
S1837	31430-15-6	Flubendazole	Flumoxanal, NSC 313680	Infection	**85.36**
S2593	150683-30-0	Tolvaptan	OPC-41061	Cardiovascular Disease	**85.33**
S4147	117772-70-0	Azithromycin Dihydrate	N/A	Infection	**85.33**
S4718	2260-50-6	Acetylcholine iodide	Acetylcolina	Neurological Diseases	**85.32**
S2596	25507-04-4	Clindamycin palmitate HCl	N/A	Infection	**85.31**
S1920	52-86-8	Haloperidol	N/A	Neurological Disease	**85.29**
S4148	7177-48-2	Ampicillin Trihydrate	NCI-C56086	Infection	**85.25**
S4671	38821-53-3	Cefradine	Cephradine, Sefril, Anspor, Cephradin, Velosef	Infection	**85.24**
S2416	9007-28-7	Chondroitin sulfate	N/A	Others	**85.15**
S4686	59-02-9	Vitamin E	Alpha-Tocopherol, D-alpha-Tocopherol, 5,7,8-Trimethyltocol|(+)-alpha-Tocopherol	Others	**85.11**
S4124	59-97-2	Tolazoline HCl	N/A	Cardiovascular Disease	**85.04**
S4821	56-40-6	Glycine	2-Aminoacetic acid, Aminoacetic acid, Glycocoll	Others	**84.88**
S4225	5370-01-4	Mexiletine HCl	KO1173	Cardiovascular Disease	**84.84**
S5027	2753-45-9	Mebeverine Hydrochloride	Duspatalin Hydrochloride, Duspatal Hydrochloride, Colofac Hydrochloride	Others	**84.83**

S5653	860-22-0	Indigo carmine		Others	**84.75**
S3753	61-90-5	L-Leucine	(S)-Leucine, Leu	Others	**84.68**
S5661	33005-95-7	Tiaprofenic acid	Tiaprofensaeure, Acido tiaprofenico	Immunology & Inflammation	**84.66**
S5538	89565-68-4	Tropisetron	ICS 205-930	Neuronal Signaling	**84.54**
S4090	5053-08-7	Fenspiride HCl	N/A	Respiratory Disease	**84.44**
S2016	85650-52-8	Mirtazapine	Org3770	Immunology	**84.43**
S4137	10402-53-6	Eprazinone 2HCl	NSC 317935	Respiratory Disease	**84.41**
S4595	27164-46-1	Cefazolin Sodium	cefazoline sodium, cephazolin sodium, Ancef	Infection	**84.41**
S5014	906673-24-3	Crisaborole (AN2728)	N/A	Metabolism	**84.26**
S4850	1847-24-1	Flucloxacillin sodium	floxacillin sodium	Microbiology	**83.97**
S4271	67814-76-0	Isepamicin Sulphate	Isepamicine (Isepamycin, sch21420) Sulphate	Infection	**83.95**
S3188	770-05-8	(+,-)-Octopamine HCl	N/A	Immunology	**83.93**
S2055	103766-25-2	Gimeracil	N/A	Neurological Disease	**83.92**
S4209	59-50-7	Chlorocresol	N/A	Infection	**83.83**
S4703	87-67-2	Choline bitartrate	2-hydroxyethyl(trimethyl)azanium;2,3,4-trihydroxy-4-oxobutanoate	Others	**83.79**
S1829	103177-37-3	Pranlukast	ONO-1078	Inflammation/Respiratory Disease	**83.77**
S5651	24584-09-6	Dexrazoxane	ICRF-187	Others	**83.71**
S1055	170364-57-5	Enzastaurin (LY317615)	N/A	Cancer	**83.69**
S2080	167221-71-8	Clevidipine Butyrate	N/A	Cardiovascular Disease	**83.63**
S2608	356-12-7	Fluocinonide	N/A	Endocrinology	**83.48**
S3048	242478-38-2	Solifenacin succinate	YM905	Cardiovascular Disease	**83.45**
S4583	94-25-7	Butamben	Butyl 4-aminobenzoate	Neurological Disease	**83.41**
S4268	530-78-9	Flufenamic acid	N/A	Inflammation	**83.40**
S5490	3737-09-5	Disopyramide	N/A	Transmembrane Transporters	**83.33**
S2455	590-63-6	Bethanechol chloride	N/A	Neurological Disease	**83.23**
S1135	150399-23-8	Pemetrexed	LY-231514	LY-231514	**83.23**
S1455	153259-65-5	Cilomilast	SB-207499	Respiratory Disease	**83.13**
S4365	131-69-1	Phthalylsulfacetamide	N/A	Others	**83.06**
S2025	64887-14-5	Urapidil HCl	N/A	Cardiovascular Disease	**83.06**
S7521	1047644-62-1	Afuresertib (GSK2110183)	N/A	Cancer	**82.94**

S5708	1229194-11-9	Edoxaban tosylate Monohydrate	DU-176b tosylate Monohydrate	Metabolism	**82.90**
S4852	86050-77-3	Gadopentetate Dimeglumine	Gadopentetic acid dimeglumine, Gd-DTPA, Meglumine gadopentetate	Others	**82.84**
S5056	38562-01-5	Dinoprost tromethamine	Dinolytic, PGF2-alpha tham, Zinoprost, Prostin F2 alpha, Dinoprost Trometamol	Others	**82.82**
P1084	16679-58-6	Desmopressin Acetate	N/A	Others	**82.79**
S4570	151-67-7	Halothane	Narcotane	Neurological Disease	**82.62**
S4357	1684-40-8	Tacrine HCl	N/A	Others	**82.54**
S4101	83480-29-9	Voglibose	AO 128	Metabolic Disease	**82.53**
S1652	103-16-2	Monobenzone	N/A	Others	**82.46**
S1190	90357-06-5	Bicalutamide	ICI-176334	Endocrinology	**82.46**
S4141	148-01-6	Dinitolmide	Zoalene	Infection	**82.30**
S1327	476-66-4	Ellagic acid	Elagostasine, Gallogen	Cancer/Cardiovascular Disease	**82.26**
S2508	114-49-8	Scopolamine HBr	N/A	Respiratory Disease	**82.24**
S2583	15318-45-3	Thiamphenicol	Thiophenicol	Cardiovascular Disease	**82.21**
S3003	363-24-6	Prostaglandin E2 (PGE2)	N/A	Others	**82.20**
S4674	630-56-8	Hydroxyprogesterone caproate	17α-Hydroxyprogesterone hexanoate, 17α-Hydroxyprogesterone caproate	Endocrinology	**82.16**
S2466	50-27-1	Estriol	NSC-12169	Neurological Disease	**82.15**
S5649	10246-75-0	Hydroxyzine pamoate		Others	**82.14**
S4530	87-89-8	i-Inositol	myo-inositol	Others	**82.14**
S4607	131-53-3	2,2′-Dihydroxy-4-methoxybenzophenone	Dioxybenzone, Dioxybenzon, Advastab 47, Cyasorb UV 24, Benzophenone-8	Others	**82.12**
S4046	313-06-4	Estradiol Cypionate	N/A	Endocrinology	**82.05**
S4655	15676-16-1	Sulpiride	Dolmatil, Dobren, Sulpyrid, Aiglonyl, Dogmatil	Neurological Disease	**82.00**
S3738	157283-68-6	Travoprost	Travoprostum	Others	**81.91**

S3684	62-51-1	Methacholine chloride	Mecholyl chloride, Acetyl-β-methylcholine chloride	Respiratory Disease	**81.89**
S4045	132-20-7	Pheniramine Maleate	N/A	Neurological Disease	**81.78**
S2393	50-70-4	Sorbitol	Glucitol	Gastroenterology	**81.75**
S1618	651-06-9	Sulfameter	N/A	Infection	**81.75**
S1508	745-65-3	Alprostadil	Prostaglandin-E1	Endocrinology	**81.69**
S4654	290297-26-6	Netupitant	CID6451149, CID 6451149, CID-6451149	Others	**81.48**
S2292	520-27-4	Diosmin	N/A	Others	**81.41**
S1876	124832-27-5	Valaciclovir HCl	N/A	Infection	**81.40**
S7995	887375-67-9	Ripasudil (K-115) hydrochloride dihydrate	N/A	Others	**81.37**
S1444	122883-93-6	Ziprasidone HCl	CP-88059	Neurological Disease	**81.29**
S4678	25655-41-8	Povidone iodine	Betadine, PVP iodine, PVP-I, Isodine	Infection	**81.22**
S1771	113-59-7	Chlorprothixene	N/A	Neurological Disease	**81.08**
S4207	882-09-7	Clofibric Acid	N/A	Metabolic Disease	**81.03**
S4698	84-80-0	Vitamin K1	N/A	Metabolism system	**81.01**
S4603	149-91-7	Gallic acid	3,4,5-Trihydroxybenzoic acid, Gallate, Benzoic acid	Cancer/Infection	**81.00**
S1443	111406-87-2	Zileuton	A-64077	Respiratory Disease	**80.89**
S1255	78281-72-8	Nepafenac	AHR 9434, AL 6515	Inflammation	**80.84**
S4360	64490-92-2	Tolmetin Sodium	N/A	Others	**80.67**
S1231	119413-54-6	Topotecan HCl	NSC609699, Nogitecan HCl, SKFS 104864A	Cancer	**80.66**
S4375	76-90-4	Mepenzolate Bromide	N/A	Others	**80.65**
S7784	23110-15-8	Fumagillin	N/A	Infection	**80.61**
S1374	364622-82-2	Doripenem Hydrate	S-4661	Infection	**80.60**
S1854	60628-96-8	Bifonazole	Bay h 4502	Infection	**80.60**
S2105	102625-70-7	Pantoprazole	N/A	Gastroenterology	**80.60**
S1475	1236699-92-5	Pimasertib (AS-703026)	MSC1936369B, SAR 245509	Cancer	**80.59**
S2378	149-64-4	Butylscopolamine Bromide	Scopolamine Butylbromide	Neurological Diseases	**80.58**
S2116	168626-94-6	Conivaptan HCl	N/A	Cardiovascular Disease	**80.54**
S1724	144598-75-4	Paliperidone	N/A	Neurological Disease	**80.40**
S7158	1231930-82-7	abemaciclib (LY2835219)	N/A	Cancer	**80.30**
S2133	60142-96-3	Gabapentin	N/A	Fanatrex, Gabarone, Neurontin	**80.24**
S4042	7177-50-6	Nafcillin Sodium	CL 8491	Endocrinology	**80.14**

S4687	123441-03-2	Rivastigmine	Rivastigmine, SDZ-ENA 713, Exelon	Neurological Disease	**80.10**
S4838	773092-05-0	Acotiamide hydrochloride	YM-443 HCl, Z-338 HCl	Neuronal Signaling	**79.98**
S1236	6035-45-6	Leucovorin Calcium Pentahydrate	Folinic acid	Cancer	**79.96**
S1262	1146699-66-2	Avagacestat (BMS-708163)	N/A	Neurological Disease	**79.80**
S4086	27833-64-3	Loxapine Succinate	N/A	Neurological Disease	**79.78**
S7215	585543-15-3	Losmapimod (GW856553X)	GW856553, GSK-AHAB	Respiratory Disease	**79.70**
S4569	146-56-5	Fluphenazine dihydrochloride	Prolixin	Neurological Disease	**79.65**
S4118	56-92-8	Histamine 2HCl	N/A	Neuronal Signaling	**79.57**
S1974	6284-40-8	Meglumine	N/A	Diagnosis	**79.50**
S1885	72509-76-3	Felodipine	CGH-869	Cardiovascular Disease	**79.48**
S2539	98079-52-8	Lomefloxacin HCl	N/A	Infection	**79.46**
S3200	322-79-2	Triflusal	UR1501	Infection	**79.44**
S2082	50-42-0	Adiphenine HCl	N/A	Cardiovascular Disease	**79.43**
S2226	870281-82-6	Idelalisib (CAL-101, GS-1101)	N/A	Cancer	**79.34**
S1516	113852-37-2	Cidofovir	HPMPC	DNA Damage	**79.29**
S1199	4291-63-8	Cladribine	2-CdA, 2-chlorodeoxyadenosine	Cancer	**79.25**
S1377	67392-87-4	Drospirenone	ZK 3059	Endocrinology	**79.20**
S3690	306-07-0	Pargyline hydrochloride	Pargylamine hydrochloride	Cardiovascular Disease	**79.20**
S4529	20098-14-0	Idramantone	Kemantane, 5-Hydroxy-2-adamantanone	Immunology	**79.19**
S4303	90-45-9	9-Aminoacridine	Aminacrine	Others	**79.08**
S2007	38194-50-2	Sulindac	N/A	Cancer	**79.02**
S4847	122547-49-3	Faropenem Sodium	N/A	Infection	**78.94**
S2381	69-65-8	D-Mannitol	Osmitrol	Cardiovascular Disease	**78.86**
P1061	76932-56-4	Nafarelin Acetate	0	Others	**78.84**
S1902	68-19-9	Vitamin B12	Cobalamin, Cyanocobalamin	Metabolic Disease	**78.83**
S1457	229975-97-7	Atazanavir Sulfate	BMS-232632	BMS-232632-05, Reyataz	**78.67**
S2556	122320-73-4	Rosiglitazone	BRL 49653	Avandia, BRL-49653	**78.58**
S5496	645-43-2	Guanethidine Monosulfate	N/A	Others	**78.57**
S4112	386750-22-7	Desvenlafaxine Succinate	WY 45233 Succinate	Neurological Disease	**78.52**
S5326	115956-12-2	Dolasetron	MDL-73147	GPCR	**78.50**
S3000	51460-26-5	Carbazochrome sodium sulfonate (AC-17)	N/A	Cancer	**78.47**
S2458	4205-91-8	Clonidine HCl	N/A	Cardiovascular Disease	**78.46**
S5712	81938-43-4	Zofenopril calcium	SQ-26991	Endocrinology & Hormones	**78.33**
S4004	1070-11-7	Ethambutol 2HCl	CL40881	Neurological Disease	**78.31**

S1425	95635-56-6	Ranolazine 2HCl	RS-43285	Cardiovascular Disease	**78.26**
S4164	69975-86-6	Doxofylline	Doxophylline	Respiratory Disease	**78.24**
S1189	170729-80-3	Aprepitant	MK-0869, L-754030	Neurological Disease	**78.12**
S3062	15307-81-0	Diclofenac Potassium	CGP-45840B	Inflammation	**78.07**
S2610	50264-69-2	Lonidamine	AF 1890, Diclondazolic Acid	Cancer	**78.02**
S5415	679809-58-6	Enoxaparin sodium	N/A	Others	**78.01**
S2110	42971-09-5	Vinpocetine	RGH-4405	Neurological Disease	**77.96**
S4590	1143-38-0	Dithranol	Anthralin; cignoline; 1,8,9-trihydroxyanthracene	Others	**77.91**
S1964	13392-28-4	Rimantadine	N/A	Infection	**77.91**
S3072	69308-37-8	(R)-baclofen	STX 209	Neurological Disease	**77.79**
S3638	42540-40-9	Cefamandole nafate	N/A	Infection	**77.77**
S5351	56796-39-5	Cefmetazole sodium	Sodium cefmetazole	Immunology	**77.74**
S2085	39133-31-8	Trimebutine	Mebutin	Gastroenterology	**77.65**
S1541	49843-98-3	Selisistat (EX 527)	SEN0014196	Neurological Disease	**77.61**
S3745	150399-21-6	Balsalazide disodium	N/A	Inflammation	**77.49**
S2547	139404-48-1	Tiotropium Bromide hydrate	BA 679BR	Neurological Disease	**77.37**
S1389	73590-58-6	Omeprazole	N/A	Metabolic Disease	**77.27**
S1068	877399-52-5	Crizotinib (PF-02341066)	N/A	Cancer	**77.12**
S3076	63585-09-1	Foscarnet Sodium	Phosphonoformate	Infection	**77.06**
S4231	54-71-7	Pilocarpine HCl	NSC 5746 HCl	Others	**77.03**
S5075	185106-16-5	Acotiamide	Acofide	Neuronal Signaling	**76.99**
S4021	134308-13-7	Tolcapone	Ro 40-7592	Metabolic Disease	**76.95**
S2514	32986-56-4	Tobramycin	NSC 180514	Infection	**76.94**
S2350	153-18-4	Rutin	Rutoside	Cardiovascular Disease	**76.89**
S2582	25332-39-2	Trazodone HCl	AF-1161, KB-831	Neurological Disease	**76.85**
S5652	1370468-36-2	Elbasvir	MK8742	Proteases	**76.73**
S2490	114-80-7	Neostigmine Bromide	N/A	Neurological Diseases	**76.72**
S7326	519055-62-0	Tasisulam	LY573636	Cancer	**76.70**
S3022	183133-96-2	Cabazitaxel	RPR-116258A, XRP6258, TXD 258	Neurological Disease	**76.63**
S4321	132-18-3	Diphenylpyraline HCl	N/A	Others	**76.62**
S4322	22059-60-5	Disopyramide Phosphate	N/A	Others	**76.61**
S5052	109889-09-0	Granisetron	Sancuso, Kevatril, Granisetronum, Sustol	Neuronal Signaling	**76.58**

P1011	148031-34-9	Eptifibatide Acetate	Mpr-Har-Gly-Asp-Trp-Pro-Cys-NH2	Angiogenesis	**76.53**
S5218	92339-11-2	Iodixanol	N/A	Others	**76.47**
S2760	842133-18-0	Canagliflozin	TA 7284, JNJ 28431754	Metabolic Disease	**76.35**
S4596	79350-37-1	Cefixime	Cefixima, Cefiximum, Cephoral, Cefspan, FR-17027, FK-027, CL-284635	Infection	**76.27**
S5662	66357-35-5	Ranitidine		Metabolic Disease	**76.19**
S4083	127-47-9	Vitamin A Acetate	Retinyl (Retinol) Acetate	Others	**75.96**
S4813	64544-07-6	Cefuroxime axetil	N/A	Microbiology	**75.89**
S4519	6080-58-6	Citric acid trilithium salt tetrahydrate	Trilithium citrate tetrahydrate, Lithium citrate tribasic tetrahydrate	Others	**75.85**
S4200	3644-61-9	Tolperisone HCl	N/A	Neurological Disease	**75.82**
S5077	1019206-88-2	Regorafenib Monohydrate	N/A	Protein Tyrosine Kinase	**75.76**
S2060	611-75-6	Bromhexine HCl	N/A	Respiratory Disease	**75.76**
S4125	1716-12-7	Sodium Phenylbutyrate	N/A	Cancer	**75.67**
S2103	16676-29-2	Naltrexone HCl	N/A	Neurological Disease	**75.66**
S4138	17692-31-8	Dropropizine	UCB-1967	Inflammation	**75.52**
S5246	142217-69-4	Entecavir	N/A	Microbiology	**75.20**
P1029	50-56-6	Oxytocin (Syntocinon)	0	Others	**75.20**
S2008	107-35-7	Taurine	N/A	Others	**75.16**
P1025	90779-69-4	Atosiban Acetate	0	Others	**75.14**
S4521	134-62-3	DEET	N,N-Diethyl-meta-toluamide, diethyltoluamide	Others	**75.04**
S4059	13755-38-9	Sodium Nitroprusside Dihydrate	N/A	Cardiovascular Disease	**74.88**
S1895	58-32-2	Dipyridamole	NSC-515776	Cardiovascular Disease	**74.86**
S1617	144-83-2	Sulfapyridine	N/A	Infection	**74.82**
S4158	104376-79-6	Ceftriaxone Sodium Trihydrate	N/A	Infection	**74.81**
S1382	21535-47-7	Mianserin HCl	ORG GB-94 HCl	Neurological Disease	**74.78**
S1004	912444-00-9	Veliparib (ABT-888)	NSC 737664	Cancer	**74.67**
S4252	55-86-7	Mechlorethamine HCl	N/A	Cancer	**74.64**
S2599	51-48-9	L-Thyroxine	NSC 36397	Neurological Disease	**74.53**
S4037	7081-53-0	Doxapram HCl	N/A	Neurological Disease	**74.52**

S2551	69388-84-7	Sulbactam sodium	CP-45899-2	Infection	**74.33**
S4076	318-98-9	Propranolol HCl	AY-64043, ICI-45520, NCS-91523	Cardiovascular Disease	**74.30**
S2529	62-31-7	Dopamine HCl	N/A	Neurological Disease	**74.29**
S4665	117976-90-6	Rebeprazole sodium	Aciphex Sodium, Dexrabeprazole Sodium, Habeprazole Sodium	Gastroenterology	**74.24**
S1915	723-46-6	e	N/A	Infection	**74.22**
S3733	394730-60-0	Boceprevir	N/A	Infection	**74.22**
S1744	59-67-6	Nicotinic Acid	N/A	Metabolic Disease	**74.08**
S2096	181183-52-8	Almotriptan Malate	LAS 31416	Cardiovascular Disease	**74.03**
S9500	1639208-54-0	Valbenazine tosylate		Others	**73.94**
S4082	8025-81-8	Spiramycin	Formacidine	Infection	**73.94**
S5286	116649-85-5	Ramatroban	BAY u 3405	Others	**73.93**
S3726	475086-01-2	Selexipag	NS-304, ACT-293987	Cardiovascular Disease/Respiratory Disease	**73.76**
S4286	166663-25-8	Anidulafungin (LY303366)	N/A	Infection	**73.46**
S4348	19562-30-2	Piromidic Acid	N/A	Others	**73.41**
S7523	1229208-44-9	Entospletinib (GS-9973)	N/A	Immunology	**73.40**
S3968	51-45-6	Histamine	N/A	Immunology & Inflammation	**73.40**
S1431	171599-83-0	Sildenafil Citrate	N/A	Viagra, Revatio	**73.36**
S1215	41575-94-4	Carboplatin	JM-8, CBDCA, NSC 241240	Cancer	**73.20**
S2459	5786-21-0	Clozapine	HF 1854, LX 100-129	Cardiovascular Disease	**73.14**
S5255	55985-32-5	Nicardipine	YC-93	Others	**72.97**
S4235	136-40-3	Phenazopyridine HCl	N/A	Cardiovascular Disease	**72.90**
S4278	51781-21-6	Carteolol HCl	N/A	Cardiovascular Disease	**72.88**
S4091	23210-58-4	Ifenprodil Tartrate	N/A	Cardiovascular Disease	**72.77**
S5619	5638-76-6	Betahistine		Neurological Disease	**72.67**
S1624	121268-17-5	Alendronate sodium trihydrate	G-704650, MK-217	Metabolic Disease	**72.61**
S2586	624-49-7	Dimethyl Fumarate	N/A	Inflammation	**72.58**
S1120	159351-69-6	Everolimus (RAD001)	N/A	Cancer	**72.57**
S4159	41859-67-0	Bezafibrate	BM 15075	Metabolic Disease	**72.47**
S4298	112901-68-5	Amifostine	N/A	Others	**72.47**
S4113	93413-62-8	Desvenlafaxine	WY 45233 Succinate	O-desmethylvenlafaxine	**72.44**
S1905	88150-42-9	Amlodipine	UK-48340	Cardiovascular Disease	**72.43**
S3687	57-13-6	E-Cardamoni	Carbamide, Carbonyldiamide, Ureophil	Others	**72.43**
S5247	220119-17-5	Selamectin	N/A	others	**72.33**

S4314	78439-06-2	Ceftazidime Pentahydrate	O.O.O.O.O.[H][C@]12SCC(C[N+]3=CC=CC=C3)=C(N1C(=O)[C@H]2NC(=O)C(=N\OC(C)(C)C(O)=O)\C1=CSC(N)=N1)C([O-])=O |r,c:8,10,12,36,t:6,32|	Others	**72.29**
S4007	140-64-7	Pentamidine isethionate	N/A	Infection	**72.23**
S4206	156-57-0	Cysteamine HCl	N/A	Metabolic Disease	**72.21**
S4114	68786-66-3	Triclabendazole	CGA-89317	Infection	**72.18**
S2589	72432-03-2	Miglitol	BAY-M-1099	Neurological Disease	**72.10**
S4262	90729-43-4	Ebastine	N/A	Inflammation	**71.95**
S1354	103577-45-3	Lansoprazole	A-65006, AG-1749	Gastroenterology	**71.92**
S1384	50924-49-7	Mizoribine	Bredinin, NSC 289637	Immunology	**71.90**
S4084	101477-54-7	Lomerizine 2HCl	KB-2796	Neurological Disease	**71.79**
S2151	956697-53-3	Sonidegib (Erismodegib, NVP-LDE225)		Cancer	**71.69**
S2577	62-44-2	Phenacetin	Acetophenetidin	Inflammation	**71.68**
S4176	77-86-1	Trometamol	N/A	Inflammation	**71.65**
S4029	1264-72-8	Colistin Sulfate	Polymixin E Sulfate	Infection	**71.63**
S7660	459789-99-2	Obeticholic Acid	INT-747, 6-ECDCA	Metabolic Disease	**71.55**
S3208	153439-40-8	Fexofenadine HCl	MDL 16455A	Neurological Disease	**71.45**
S5478	24868-20-0	Dantrolene sodium	sodium;3-[(E)-[5-(4-nitrophenyl)furan-2-yl]methylideneamino]-5-oxo-4H-imidazol-2-olate DANTROLENE SODIUM Dantrolene sodium salt Dantrium Sodium dantrolene 14663-23-1 Dantrolene (sodium) Dantrolene sodium anhydrous UNII-28F0G1E0VF Dantamacrin Dantrix Dantr	Others	**71.41**

S4691	131-57-7	Oxybenzone	Oxybenzone, Eusolex 4360, Escalol 567, KAHSCREEN BZ-3, Benzophenone 3	Others	**71.36**
S4544	51-79-6	Urethane	Carbamic acid ethyl ester, Ethyl carbamate, Ethylurethane	Neurological Disease	**71.28**
S2169	147098-20-2	Rosuvastatin Calcium	ZD4522	Cardiovascular Disease/Metabolic Disease	**71.27**
S5010	1330-43-4	Indometacin Sodium		Others	**71.26**
S4898	102-65-8	Sulfalozine sodium	sulfaclozine sodium, sulfachlopryrazine sodium	Infection	**71.23**
S4693	29110-48-3	Guanfacine Hydrochloride	Guanfacine Hydrochloride, Guanfacine, Tenex, Intuniv	Cardiovascular Disease	**71.19**
S1933	124-94-7	Triamcinolone	N/A	Inflammation	**71.11**
S1228	57852-57-0	Idarubicin HCl	4-demethoxydaunorubicin (NSC256439, 4-DMDR) HCl	DNA Damage	**70.97**
S8103	1018899-04-1	Sotagliflozin (LX4211)	N/A	Metabolic Disease	**70.85**
S2481	89226-50-6	Manidipine	N/A	Cardiovascular Disease	**70.82**
S3186	3978-86-7	Azatadine dimaleate	SCH10649	Inflammation	**70.82**
S1338	60142-95-2	Gabapentin HCl	N/A	Neurological Disease	**70.59**
S4736	15421-84-8	Trapidil	Rocornal, Trapymin, Avantrin, Trapymine	Cardiovascular Disease	**70.54**
S7952	1306760-87-1	Ozanimod (RPC1063)	N/A	Immunology	**70.49**
S8051	441798-33-0	Macitentan	ACT 064992	Cardiovascular Disease/Respiratory Disease	**70.43**
S4602	546-88-3	Acetohydroxamic acid	N-Hydroxyacetamide, Methylhydroxamic acid, Acetic acid|oxime, Lithostat	Infection	**70.33**
S4049	181695-72-7	Valdecoxib	N/A	Neurological Disease	**70.31**
S2232	74050-98-9	Ketanserin	R41468	Neuronal Signaling	**70.20**
S1300	17902-23-7	Tegafur (FT-207, NSC 148958)	Fluorafur	Cancer	**70.15**
S4514	99-15-0	Acetylleucine	N-acetyl-L-leucine	Neurological Disease	**70.13**
S5252	189224-26-8	Ozagrel sodium	KCT-0809, Cataclot, Xanbo	Others	**70.09**

S5287	14176-50-2	Tiletamine Hydrochloride		Others	**70.03**
S4020	10040-45-6	Sodium Picosulfate	N/A	Metabolic Disease	**70.01**
S2462	120011-70-3	Donepezil HCl	N/A	Neurological Disease	**69.91**
S1679	5104-49-4	Flurbiprofen	N/A	Inflammation	**69.91**
S4198	96-50-4	Aminothiazole	N/A	Infection	**69.80**
S4060	1264-62-6	Erythromycin Ethylsuccinate	N/A	Infection	**69.75**
S1934	62997-67-5	Nystatin (Fungicidin)	N/A	Infection	**69.70**
S4031	320345-99-1	Aclidinium Bromide	LAS 34273, LAS-W 330	Neurological Disease	**69.53**
S5399	6469-93-8	Chlorprothixene hydrochloride	chlorprothixene HCl	Antipsychotic	**69.49**
S3175	82248-59-7	Atomoxetine HCl	LY 139603 HCl	Neurological Disease	**69.47**
S3160	297-76-7	Ethynodiol diacetate	8080 CB	Endocrinology	**69.45**
S4095	23674-86-4	Difluprednate	CM 9155	Endocrinology	**69.45**
S1979	19774-82-4	Amiodarone HCl	N/A	Cardiovascular Disease	**69.43**
S4024	80-49-9	Homatropine Methylbromide	N/A	Gastroenterology	**69.43**
S2066	75438-57-2	Moxonidine	BDF5895	Cardiovascular Disease	**69.35**
S1835	83905-01-5	Azithromycin	CP-62993, XZ-450	CP-62993, XZ-450	**69.16**
S7157	142880-36-2	Ilomastat (GM6001, Galardin)	N/A	Cancer	**69.10**
S4940	118-71-8	Maltol	Larixinic acid, Palatone, Veltol	Others	**69.07**
S4301	56715-13-0	(R)-(+)-Atenolol HCl	N/A	Others	**69.04**
S1192	112887-68-0	Raltitrexed	ZD-1694	Cancer	**69.02**
S4555	2037-95-8	Carsalam	Carbonylsalicylamide	Inflammation	**69.02**
S1773	79-57-2	Oxytetracycline (Terramycin)	N/A	Infection	**68.98**
S1188	120511-73-1	Anastrozole	ZD-1033	Endocrinology	**68.94**
S1185	155213-67-5	Ritonavir	ABT-538, A 84538	Infection	**68.90**
S2565	34642-77-8	Amoxicillin Sodium	Clavulanate	Infection	**68.68**
S2422	35212-22-7	Ipriflavone (Osteofix)	N/A	Metabolic Disease	**68.43**
S3165	188062-50-2	Abacavir sulfate	1592U89	Infection	**68.29**
S5488	2321-07-5	Fluorescein		Others	**68.27**
S2486	3160-91-6	Moroxydine HCl	N/A	Infection	**68.24**
S1639	161814-49-9	Amprenavir	141W94, VX-478, KVX-478	Infection	**68.15**
S2868	850649-62-6	Alogliptin（SYK-322）benzoate	SYR-322	Immunology/Metabolic Disease	**68.09**
S2907	53179-13-8	Pirfenidone	S-7701,AMR-69	Inflammation/Respiratory Disease	**68.07**
S1987	83919-23-7	Mometasone furoate	SCH-32088	Inflammation	**68.05**
S1039	53123-88-9	Rapamycin (Sirolimus)	AY 22989，NSC-2260804	Immunology	**67.96**
S4401	2139-47-1	Nifenazone	N/A	Others	**67.92**

S4684	139755-83-2	Sildenafil	Revatio, UK-92480, Viagra	Cardiovascular Disease/Respiratory Disease	**67.76**
S3064	23828-92-4	Ambroxol HCl	N/A	Respiratory Disease	**67.74**
S5100	94-63-3	Pralidoxime Iodide	2-PAM	Neuronal Signaling	**67.66**
S4050	175865-59-5	HCl	N/A	Endocrinology	**67.65**
S5311	54-47-7	Pyridoxal phosphate	pyridoxal 5'-phosphate, PAL-P, PLP, Vitamin B6 phosphate	Metabolism	**67.59**
S4054	52-01-7	Spironolactone	N/A	Endocrinology	**67.47**
S4027	3717-88-2	Flavoxate HCl	NSC-114649	Neurological Disease	**67.31**
S2119	23288-49-5	Probucol	DH-581	Cardiovascular Disease	**67.26**
S1259	196597-26-9	Ramelteon	TAK-375	Neurological Disease	**67.25**
S1407	155206-00-1	Bimatoprost	N/A	Others	**67.24**
S1332	78755-81-4	Flumazenil	RO 15-1788	Neurological Disease	**67.19**
S5063	1029877-94-8	Trelagliptin succinate	N/A	Proteases	**66.99**
S4179	42057-22-7	Mezlocillin Sodium	N/A	Infection	**66.70**
S4185	51-24-1	Tiratricol	N/A	Endocrinology	**66.52**
S4266	70359-46-5	Brimonidine Tartrate	N/A	Others	**66.21**
S2057	6055-19-2	Cyclophosphamide Monohydrate	N/A	Cancer	**66.11**
S7810	850140-73-7	Afatinib (BIBW2992) Dimaleate	N/A	Cancer	**65.95**
S2090	145108-58-3	Dexmedetomidine HCl	N/A	Neuronal Signaling	**65.92**
S1937	54-85-3	Isoniazid	N/A	Infection	**65.90**
S5582	69-74-9	Cytarabine hydrochloride	N/A	DNA Damage	**65.86**
S3944	99-66-1	Valproic acid	2-Propylvaleric Acid, Sodium valproate	Epigenetics	**65.84**
S3204	59263-76-2	Meptazinol HCl	N/A	Neurological Disease	**65.82**
S4512	2490-97-3	Aceglutamide	α-N-Acetyl-L-glutamine, N2-Acetylglutamine	Neurological Disease	**65.77**
S4023	51-05-8	Procaine HCl	Novocaine HCl	Neurological Disease	**65.75**
S5393	2624-43-3	Cyclofenil	F-6066; H-3452	Metabolism	**65.67**
S4038	61-12-1	Dibucaine HCl	Cinchocaine HCl	Endocrinology	**65.62**
S4131	99291-25-5	Levodropropizine	N/A	Respiratory Disease	**65.47**
S2521	51-42-3	Epinephrine bitartrate	Adrenalinium	Cancer	**65.42**
S4834	50-34-0	Propantheline bromide	Pro-Banthine, Neometantyl, Neopepulsan	Neuronal Signaling	**65.41**
S4079	274693-27-5	Ticagrelor	AZD 6140	Cardiovascular Disease	**65.36**

S3980	65-23-6	Pyridoxine	Pyridoxol, Vitamin B6, Gravidox	Metabolism	**65.31**
P1056	16941-32-5	Glucagon HCl	H-His-Ser-Gln-Gly-Thr-Phe-Thr-Ser-Asp-Tyr-Ser-Lys-Tyr-Leu-Asp-Ser-Arg-Arg-Ala-Gln-Asp-Phe-Val-Gln-Trp-Leu-Met-Asn-Thr-OH	Angiogenesis	**65.31**
S4081	127-56-0	Sulfacetamide Sodium	N/A	Immunology & Inflammation	**65.26**
S4047	603-50-9	Bisacodyl	N/A	Cardiovascular Disease	**65.07**
S2101	56974-61-9	Gabexate Mesylate	N/A	Cancer/Cardiovascular Disease/Immunology	**65.04**
S4099	138530-94-6	Dexlansoprazole	T 168390，TAK 390	Cardiovascular Disease	**64.93**
S4584	94-26-8	Butylparaben	Butyl parahydroxybenzoate, Butyl paraben, Butyl 4-hydroxybenzoate	Infection	**64.89**
S4729	106463-17-6	Tamsulosin hydrochloride	Tamsulosina hydrochloride, Tamsulosinum hydrochloride	Urology	**64.85**
S2376	1407-03-0	Ammonium Glycyrrhizinate	AMGZ, Glycyram	Inflammation	**64.63**
S4009	223673-61-8	Mirabegron	YM 178	Cancer	**64.35**
S4290	20830-75-5	Digoxin	N/A	Cardiovascular Disease	**64.17**
S4664	25717-80-0	Molsidomine	SIN-10, Corvaton, Morsydomine	Cardiovascular Disease	**63.92**
S4069	55-97-0	Hexamethonium? Dibromide	N/A	Cardiovascular Disease	**63.86**
S4167	66215-27-8	Cyromazine	N/A	Infection	**63.85**
S1025	184475-35-2	(ZD1839)	N/A	Cancer	**63.74**
S3636	66592-87-8	hydrate	N/A	Infection	**63.74**
S1091	867160-71-2	Linsitinib (OSI-906)	N/A	Cancer	**63.67**
S2123	50-99-7	Dextrose	D-glucose	Others	**63.64**
S1994	103890-78-4	Lacidipine	GX-1048,GR-43659X,SN-305	Cardiovascular Disease	**63.59**
S1890	76963-41-2	Nizatidine	N/A	Gastroenterology	**63.52**
S4591	4008-48-4	Nitroxoline	8-Hydroxy-5-nitroquinoline, 5-nitroquinolin-8-ol, 5-Nitro-8-quinolinol|5-Nitro-8-hydroxyquinoline	Infection	**63.48**
S4142	2971-90-6	Clopidol	WR 61112	Infection	**63.46**

S4287	208538-73-2	Micafungin Sodium	FK463 Sodium, Mycamine Sodium	Infection	**63.32**
S1023	183319-69-9	Erlotinib HCl (OSI-744)	(CP358774, NSC 718781) HCl	(CP358774, NSC 718781) HCl	**63.13**
S1227	82640-04-8	Raloxifene HCl	LY156758 (Keoxifene) HCl	Endocrinology	**63.02**
S4026	2192-20-3	Hydroxyzine 2HCl	N/A	Neurological Disease	**63.01**
S4184	39809-25-1	Penciclovir	BRL-39123, VSA 671	Infection	**62.91**
S1526	950769-58-1	Quizartinib (AC220)	N/A	Cancer	**62.85**
S4292	3254-89-5	Diphenidol HCl	N/A	Neurological Disease	**62.77**
S5480	3485-62-9	Clidinium Bromide	N/A	Neuronal Signaling	**62.74**
S2467	104227-87-4	Famciclovir	BRL-42810	Cancer	**62.62**
S2075	302543-62-0	Rosiglitazone HCl	BRL-49653 HCl	Cardiovascular Disease	**62.60**
S1747	66085-59-4	Nimodipine	BAY E 9736	Cardiovascular Disease	**62.55**
S4250	80-35-3	Sulfamethoxypyridazine	CL 13494	Infection	**62.43**
S1978	155-41-9	Methscopolamine	N/A	Neurological Disease	**62.41**
S4580	123-31-9	Hydroquinone	Quinol, 1,4-Benzenediol, 1,4-Dihydroxybenzene, HQ	Others	**62.41**
S4243	56-47-3	Deoxycorticosterone acetate	N/A	Endocrinology	**62.36**
S1941	76095-16-4	Enalapril Maleate	MK-421 Maleate	Cardiovascular Disease	**62.34**
S4237	63-45-6	Primaquine Diphosphate	N/A	Infection	**62.26**
S8205	1446502-11-9	Enasidenib (AG-221)	N/A	Cancer	**62.16**
S2908	31282-04-9	Hygromycin B	N/A	Infection	**62.11**
S1998	68-41-7	D-Cycloserine	RO-1-9213	Infection	**62.00**
S2102	161735-79-1	Rasagiline Mesylate	TVP-1012	Cardiovascular Disease	**61.97**
S2369	7085-55-4	Troxerutin	N/A	Cardiovascular Disease	**61.86**
S7772	143664-11-3	Elacridar (GF120918)	GW120918	Cancer	**61.78**
S5482	1786-81-8	Prilocaine hydrochloride	N/A	Ion-Channel	**61.66**
S8034	608141-41-9	Apremilast (CC-10004)	N/A	Immunology	**61.64**
S3001	163252-36-6	Clevudine	N/A	Infection	**61.39**
S5664	341-69-5	Orphenadrine Hydrochloride		Others	**61.18**
S4414	94-16-6	Sodium 4-aminohippurate Hydrate	N/A	Others	**61.13**
S1130	781661-94-7	YM155 (Sepantronium Bromide)	N/A	Cancer	**60.99**
S2461	57808-66-9	Domperidone	N/A	Neurological Disease	**60.96**
S5702	583-03-9	Fenipentol		Others	**60.94**

S4606	70-18-8	Glutathione	Isethion, Glutathion, Tathion	Inflammation	**60.83**
S3953	657-27-2	L-Lysine hydrochloride	N/A	Others	**60.75**
S1653	302-79-4	Tretinoin	All-trans Retinoic Acid	Cancer	**60.72**
S4192	72-80-0	Chlorquinaldol	N/A	Infection	**60.63**
S5084	69-81-8	Carbazochrome	Adedolon, Adchnon, Adrenostazin, Cromadrenal, Adona, AC-17	Others	**60.60**
S1426	135062-02-1	Repaglinide	AG-EE 623 ZW	Endocrinology	**60.58**
S4105	61438-64-0	Closantel Sodium	N/A	Infection	**60.53**
S4609	22494-42-4	Diflunisal	Dolobid, Dolobis, Flovacil, Fluniget	Inflammation	**60.29**
S4064	219861-08-2	Escitalopram Oxalate	N/A	Neurological Disease	**60.28**
S1928	26750-81-2	Alibendol	EB 1856	Neurological Disease	**60.27**
S7015	1260251-31-7	Birinapant	TL32711	Cancer	**60.18**
S1518	57469-77-9	Ibuprofen Lysine	N/A	Inflammation	**60.13**
S2533	23239-51-2	Ritodrine HCl	NSC 291565	Others	**60.05**
S4345	6493-05-6	Pentoxifylline	N/A	Others	**60.01**
S5567	127-48-0	Trimethadione	N/A	Others	**59.96**
S1060	763113-22-0	Olaparib (AZD2281, Ku-0059436)	N/A	Cancer	**59.93**
S3056	58066-85-6	Miltefosine	Hexadecylphosphocholine	Infection	**59.89**
S4135	6933-90-0	Clorprenaline HCl	NSC 334693	Respiratory Disease	**59.88**
S4844	147816-24-8	Cefcapene Pivoxil	N/A	Infection	**59.78**
S1276	106685-40-9	Adapalene	CD-271	Inflammation	**59.64**
S1514	79217-60-0	Cyclosporine	N/A	Immunology	**59.61**
S4328	60-02-6	Guanethidine Sulfate	N/A	Others	**59.34**
S5491	98079-51-7	Lomefloxacin		Infection	**59.31**
S7799	945614-12-0	Pexmetinib (ARRY-614)	N/A	Cancer	**59.26**
S3752	73-32-5	isoleucine	Ile	Others	**59.09**
S3729	8047-67-4	Iron sucrose	Iron saccharate, Sucroferric oxyhydroxide	Others	**58.99**
S3185	62-13-5	Adrenalone HCl	N/A	Cardiovascular Disease	**58.76**
S4028	55203-24-2	Dexamethasone Sodium Phosphate	N/A	Immunology & Inflammation	**58.33**
S2021	80-77-3	Chlormezanone	N/A	Respiratory Disease	**58.15**
S5566	960404-48-2	Dapagliflozin propanediol monohydrate	N/A	GPCR & G Protein	**58.01**
S1853	52-67-5	Penicillamine	Dimethyl Cysteine	Infection	**57.94**
S4119	149676-40-4	Pefloxacin Mesylate Dihydrate	1589 RB	Infection	**57.86**

S7910	1204669-58-8	Epacadostat (INCB024360)	N/A	Cancer	**57.84**
S1565	936727-05-8	VX-809 (Lumacaftor)	VRT 826809	Gastroenterology/Respiratory Disease	**57.83**
S4001	1140909-48-3	Cabozantinib malate (XL184)	N/A	Cancer	**57.77**
S5501	50-03-3	Hydrocortisone acetate	Hydrocortisone 21-acetate	Nervous system	**57.51**
S1432	103628-48-4	Sumatriptan Succinate	GR 43175	Neurological Disease	**57.33**
S1394	5189-11-7	Pizotifen Malate	BC-105	Inflammation	**57.12**
S1334	75507-68-5	Flupirtine maleate	N/A	Neurological Disease	**56.99**
S4274	99755-59-6	Rotigotine	N/A	Neurological Disease	**56.81**
S4394	37693-01-9	Clofoctol	N/A	Others	**56.76**
S4700	56-12-2	4-Aminobutyric acid	4-Aminobutanoic acid, GABA, Gamma-aminobutyric acid, Piperidic acid	Neurological Diseases	**56.65**
S2127	85416-73-5	S- (+)-Rolipram	N/A	Immunology/Neurological Disease	**56.47**
S7108	1269440-17-6	Encorafenib (LGX818)	N/A	MAPK	**56.29**
S4305	80-50-2	Anisotropine Methylbromide	N/A	Others	**56.27**
S4244	153-98-0	Serotonin HCl	5-HT HCl	Neurological Disease	**56.21**
S2485	70476-82-3	Mitoxantrone 2HCl	NSC-301739	Cardiovascular Disease	**56.17**
S1956	22832-87-7	Miconazole Nitrate	NSC 169434 Nitrate	Infection	**56.11**
S4668	59-46-1	Procaine	Procaine, Novocaine, Vitamin H3, Duracaine, Spinocaine	Procaine, Novocaine, Vitamin H3, Duracaine, Spinocaine	**55.75**
S8022	864070-44-0	Empagliflozin (BI 10773)	N/A	Metabolic Disease	**55.60**
S4233	61-80-3	Zoxazolamine	NSC 24995, Contrazole	Neurological Disease	**55.48**
S1547	144060-53-7	Febuxostat	TMX 67, TEI-6720	Inflammation	**55.33**
P1030	47931-85-1	Salmon Calcitonin	0	Others	**54.92**
S2543	103980-44-5	Ceftiofur HCl	U-67279A	Infection	**54.85**
S7829	21919-05-1	CB1954	Tretazicar	Cancer	**54.52**
S5372	79416-27-6	Methyl Aminolevulinate Hydrochloride	Methyl 5-amino-4-oxopentanoate hydrochloride; Methyl 5-Aminolevulinate Hydrochloride; 5-Amino-4-oxopentanoic Acid Methyl Ester Hydrochloride	Others	**54.38**
S4230	21256-18-8	Oxaprozin	WY-21743	Inflammation	**54.38**

S5278	856867-55-5	Tedizolid		Infection	**54.27**
S2357	22888-70-6	Silibinin	Silybin	Cancer	**54.18**
S2605	58186-27-9	Idebenone	CV-2619	Inflammation	**53.69**
S4161	65-85-0	Benzoic Acid	FEMA 2131	Neurological Disease	**53.68**
S2097	177036-94-1	Ambrisentan	LU-208075,BSF-208075	Neurological Disease	**53.28**
S1403	220620-09-7	Tigecycline	GAR-936	Infection	**53.15**
S4072	541-22-0	Decamethonium Bromide	N/A	Neurological Disease	**52.62**
S2493	132539-06-1	Olanzapine	LY170053	Neurological Disease	**52.54**
S4820	9000-92-4	Diastase	N/A	Others	**52.39**
S4041	6054-98-4	Olsalazine Sodium	N/A	Inflammation	**52.02**
S4197	64-65-3	Bemegride	N/A	Neurological Disease	**51.65**
S2005	518048-05-0	Raltegravir (MK-0518)	N/A	Immunology	**51.41**
S4149	61618-27-7	Amfenac Sodium Monohydrate	N/A	Inflammation	**51.37**
S4128	30751-05-4	Troxipide	N/A	Gastroenterology	**51.26**
S4175	57-67-0	Sulfaguanidine	N/A	Infection	**51.04**
S1500	378-44-9	Betamethasone	NSC-39470, SCH-4831	Celestone, Betadexamethasone, Flubenisolone, Sch-4831, NCS-39470	**50.89**
S4191	3440-28-6	Betamipron	CS-443	Others	**50.52**
S4582	96020-91-6	Eflornithine hydrochloride hydrate	Difluoromethylornithine hydrochloride hydrate	Others	**50.26**
S1787	29767-20-2	Teniposide	NSC 122819, VM-26	Cancer	**50.17**
S4229	5987-82-6	Oxybuprocaine HCl	N/A	Neurological Disease	**50.17**
S4679	63074-08-8	Terazosin HCl	Terazosin, Hytrin, Zayasel, Terazosine, Flumarc, Fosfomic, Blavin	Cardiovascular Disease	**50.02**
S1026	220127-57-1	Imatinib Mesylate (STI571)	CGP-57148B, ST-1571 Mesylate	Glivec, CGP-57148B, STI-571	**49.45**
S1717	7554-65-6	Fomepizole	4-methylpyrazole, Antizol, Antizol-Vet	Metabolic Disease	**48.17**
S4165	132-69-4	HCl	AF864	Inflammation	**47.61**
P1004	128270-60-0	Bivalirudin Trifluoroacetate	0	Others	**46.86**
S2492	1476-53-5	Novobiocin Sodium	N/A	Cardiovascular Disease	**45.61**
S4039	554-57-4	Methazolamide	CL 8490	Neurological Disease	**45.24**
S1312	18883-66-4	Streptozotocin (STZ)	NSC-85998	Cancer	**42.29**
S1149	122111-03-9	Gemcitabine HCl	LY188011	Cancer	**38.07**
S2570	52-21-1	Prednisolone Acetate	Omnipred	Immunology	**37.35**

S1648	147-94-4	Cytarabine	N/A	Cancer	**31.56**
S1119	849217-68-1	Cabozantinib (XL184, BMS-907351)	N/A	BMS-907351	**31.47**
S1218	123318-82-1	Clofarabine	N/A	Cancer	**29.06**
S3646	54-64-8	Thimerosal	Thiomersalate, Mercurothiolate	Infection	**27.37**
S1214	9041-93-4	Bleomycin sulfate	Blenoxane		**22.48**
